# Genetic susceptibility to infectious diseases: big is beautiful, but will bigger be even better?

**DOI:** 10.1016/S1473-3099(06)70601-6

**Published:** 2006-10

**Authors:** David Burgner, Sarra E Jamieson, Jenefer M Blackwell

**Affiliations:** a School of Paediatrics and Child Health, University of Western Australia, Princess Margaret Hospital for Children, Perth, WA, Australia; b Cambridge Institute for Medical Research, University of Cambridge School of Clinical Medicine, Addenbrooke's Hospital, Cambridge, UK

## Abstract

Genetic epidemiology, including twin studies, provides robust evidence that genetic variation in human populations contributes to susceptibility to infectious disease. One of the major limitations of studies that attempt to identify the genes and mechanisms that underlie this susceptibility has been lack of power caused by small sample size. With the development of novel technologies, burgeoning information on the human genome, the HapMap project, and human genetic diversity, we are at the beginning of a new era in the study of the genetics of complex diseases. This review looks afresh at the epidemiological evidence that supports a role for genetics in susceptibility to infectious disease, examines the somewhat limited achievements to date, and discusses current advances in methodology and technology that will potentially lead to translational data in the future.

## Introduction

Infection is one of the leading causes of human mortality and morbidity, with much of the burden falling on children.^1^ Infectious diseases are a major selective pressure,^2^, ^3^, ^4^, ^5^ and the genes involved in the immune response are the most numerous and diverse in the human genome,^6^ indicating the evolutionary advantages of a varied immunological response to a wide range of infectious pathogens.^7^ This is most obvious at the HLA loci,^8^ the prototypical candidate genetic region for infectious disease susceptibility. For example, individuals in whom all class II HLA alleles are heterozygous are more likely to clear hepatitis B infection,^9^ and those with heterozygous class I alleles progress from HIV to an AIDS-defining illness more slowly and have lower mortality.^10^ The converse scenario, increased HLA homozygosity, may contribute to the increased susceptibility to infection in genetically isolated populations.^11^

There is huge variation in the individual outcomes that follow exposure to potentially life-threatening pathogens, and this differential susceptibility partly shows the functional genetic diversity of the immune response. Here we provide an overview of human genetic susceptibility in infectious diseases, review the supporting epidemiological evidence, discuss current methodological and technological issues, and highlight the somewhat limited achievements to date, especially those that have increased our understanding of outcome and treatment response for specific infectious diseases.

## One gene…or many?

Susceptibility to infection and many other human diseases (including diabetes and ischaemic heart disease) arises from the complex interaction of environmental and host genetic factors. In general, many genetic loci make modest contributions to human disease susceptibility (ie, they are genetically complex), and most of the focus in the field has been on identifying these loci and their effects in infection and in other conditions. However, there are many single-gene (Mendelian) disorders affecting immune function, with over 300 primary immunodeficiencies reported. Although often rare, these profound immunodeficiencies stem from major functional aberrations at single genes and can be highly informative about immunological mechanisms and protection against specific infections.^12^, ^13^ There are also important examples of more common single-gene disorders that markedly influence infection-specific susceptibility; the best known is the protection afforded by sickle cell heterozygosity against falciparum malaria.^14^ A variant in the Duffy antigen gene promoter, which results in lack of erythrocyte surface expression, prevents binding by *Plasmodium vivax*, and is therefore protective.^15^, ^16^ More recently, a genetic variant that determines blood group secretor status has been shown to mediate susceptibility to Norwalk virus,^17^ and also to affect HIV progression independently of other chemokine variants.^18^

## Hype and hyperbole

There has been substantial expectation that an increased understanding of the genetic determinants of differential susceptibility and outcome would be rapidly translated into novel therapies and preventative interventions. Much of this expectation has yet to be realised, partly because of the methodological limitations of earlier studies and a tendency to examine individual genes in isolation and without consideration of their genetic and environmental milieu. Thus many reported genetic associations have not been replicated in subsequent studies.^19^ Understandably, some scepticism exists as to how much a genetic epidemiological approach can actually reveal about the genetic basis of complex diseases.^20^ However, there have been many recent advances in technology and—more latterly—its application; such studies have identified important protective and pathogenic pathways, and have led to individually targeted anti-infective therapies. Hopefully, we are entering an era in which well designed studies will begin to deliver robust genetic data and many more opportunities for translation into better treatments.

## Epidemiological evidence

The idea that human genetics may contribute to our understanding of the susceptibility to infectious diseases is not new. As early as the 18th century, differential susceptibility to infection was suggested to be a characteristic of the host, and diseases such as tuberculosis and leprosy were believed to be inherited defects.^21^ Infectious diseases, like other phenotypes, may exhibit familial aggregation: a greater frequency of the disease in relatives of infected individuals compared with relatives of those without disease. Studies of familial aggregation can therefore investigate the importance of shared determinants, both environmental and genetic, in disease susceptibility. They usually use a form of the recurrence risk ratio, essentially the ratio of prevalence in the relatives of the index case to the prevalence in the general population. It is important in such studies that the disease phenotype is carefully defined, particularly in the context of infectious diseases. Interpretation of recurrence risk is more straightforward if the phenotype is a binary or dichotomous trait (eg, the presence or absence of meningococcal septicaemia) than a qualitative range of clinical phenotypes, which may be less consistent (eg, measures of severity of infection determined by treatment interventions). Familial aggregation measures make no supposition about the cause of such aggregation, and recurrence risks within the same generation, such as sibling risk ratios, will identify both shared environmental and genetic effects. In infectious diseases, transmission of pathogens between family members may increase the risk of disease and contribute to the observed phenotypic aggregation. For example, in a study of sibling recurrence risk ratio for meningococcal disease, there was a 30 times increased risk of infection in siblings of cases compared with the general population risk, but the risk decreased with a longer time interval between the index case and the sibling case.^22^ The recurrence risk for meningococcal disease in siblings was 11·9 times the population risk when the index and sibling cases occurred more than a week apart, falling to a risk of 8·2 if a year had elapsed between cases.^22^ These data indicate significant genetic determinants of meningococcal susceptibility, and also the importance of common acquisition of virulent strains within families, which accounts for the more immediate increased risk in household contacts.^23^

### Twin studies

Studies of twins can also provide an estimate of the relative contributions of shared genes and environment to disease phenotypes, by comparing the risk in genetically identical monozygotic twin pairs to that in dizygotic twins, who share on average only half their genes. Some twin studies of infectious diseases are historical,^24^, ^25^, ^26^ and determinations of zygosity and disease phenotype may be inaccurate.^25^ Notwithstanding these reservations, susceptibility to some infections show markedly increased concordance in monozygotic compared with dizygotic twins.^27^ Examples include tuberculosis,^24^, ^25^, ^26^ sinusitis,^28^ *Helicobacter pylori*-specific antibody titres,^29^ and leprosy.^30^ More recent twin and triplet data indicate that susceptibility to both acute and chronic otitis media is largely genetically determined.^31^, ^32^ The relative contributions of infection, atopy, and cranio-facial anatomy, all of which are determined in part by genetic factors, remain unclear.

Twin studies may also provide useful insights into the determinants of disease outcome. Carrier status for hepatitis B virus^33^ and the febrile response to (but not acquisition of) *Plasmodium falciparum*^34^ both indicate increased concordance in monozygotic twin pairs. A study of the severity of common childhood infections, which used hospital admission as the phenotype, indicated that distinct genetic factors were likely to be involved in different infections, since the risk of admission in the second twin was only increased if the twins both had the same infectious diagnosis (Burgner D, unpublished). The concordance rates for common otolaryngological procedures such as myringotomy and adeno-tonsillectomy, which are surrogates for disease severity, also show greatly increased concordance in monozygotic twin pairs (Burgner D, unpublished). Twin studies of vaccine responses, in which the antigenic stimulus is unambiguous, indicate significant genetic influences on both humoral^35^ and cell-mediated^36^ responses. More detailed genetic studies indicate the importance of both HLA and non-HLA loci in vaccine responsiveness.^37^, ^38^ Studies in vitro of twins^39^ and first-degree relatives^40^ also show the importance of genetic factors in determining the innate immune responses to infectious stimuli.

A twin study of *H pylori* acquisition, which compared concordance rates for twins reared either together or apart, gave very similar measures of genetic influences in each setting, which accounted for most of the disease risk.^29^ However, twins and siblings are very commonly reared together, making it difficult to tease out the relative contributions of genetic factors and shared environmental exposures, particularly pathogen sharing. Intergenerational studies may circumvent this issue. A seminal study compared the risk and shared causes of premature mortality in children adopted very early in life with that of their biological and adoptive parents.^41^ The risk of an adopted child dying of infection was increased almost six times if their biological parent had also died prematurely of infection, whereas no increased risk was observed if the adoptive parent died of infection, suggesting that genetic rather than environmental factors are the important determinants of infectious disease mortality. Much smaller genetic effects were noted for cardiovascular disease and malignancy.^41^

## Study design and methodological issues

These epidemiological data indicate that genetic factors influence both susceptibility to, and outcome of, infection. The various study designs and methodological issues are important in interpreting previous data and in designing contemporary and future studies in infection. As the fundamentals of genetic epidemiology have recently been reviewed in detail,^42^, ^43^, ^44^, ^45^, ^46^, ^47^, ^48^ only a brief overview will be provided here. Some of the terminology is explained briefly in the panel. Of note, although single nucleotide polymorphisms (SNPs) are the workhorse of current high-throughput genotyping technologies, other forms of genetic variation are also common in the human genome and may have important effects on disease risk. These include repeating sequence motifs (microsatellites and minisatellites), insertions and deletions, and differences in gene copy number.^55^ For example, recent data indicate that there is a strong inverse correlation between the number of copies of an HIV suppressive chemokine gene (*CCL3L1*) and HIV susceptibility.^56^

Panel **Definitions for genetic terminology**

**Allelic association**

Statistical analysis to determine whether disease is associated with particular allelic variants at one or more loci, usually by comparing marker allele frequencies between a disease group and a control group.

***F***_st_ **statistic**

A statistic to describe individuals, subpopulations, and whole population structures. Used to assess evidence for selection by comparing the frequency of an allele in different populations.^49^

**Haplotype**

A combination of marker alleles at different markers along the same chromosome that tend to be inherited as a unit.

**Haplotype tag**

An SNP that acts as a defining marker for a haplotype by virtue of linkage disequilibrium. Analysing haplotype tags, rather than all the variants on a haplotype, reduces redundancy and genotyping costs.

**Heterozygosity**

A measure of genetic diversity within a population.

**Linkage analysis**

Statistical analysis to localise genes and markers with respect to each other in the genome, based on recombination frequency. Linkage analysis can also be used to map a disease phenotype in relation to polymorphic markers.

**Linkage disequilibrium**

The non-random association of alleles at two or more neighbouring loci that are inherited together more often than expected by chance, providing the basis to haplotypes and linkage disequilibrium mapping. This association can potentially derive from population admixture.

**Microsatellite**

A polymorphism characterised by a variable number of tandem repeats, often defined by the numbers of repeats in a row of at least two or more nucleotides. They are useful markers for family linkage studies and determining a person's unique DNA fingerprint in forensic medicine. Also known as SSR (simple sequence repeat) or STR (simple tandem repeat).

**Population admixture**

The recent combination of two or more previously distinct populations. Unknown admixture may result in spurious genetic associations in a case-control study design.

**SNP**

Single nucleotide polymorphism, single base-pair change at a specific point in the genome.

**SNP chip**

Array technology that allows large numbers (up to a million) of SNPs to be genotyped on a single array typically the size of a microscope slide (eg, Affymetrix^50^ or Illumina^51^)

Additional useful references: Burton et al,^42^ Malats and Calafell,^52^, ^53^ and Calafell and Malats.^54^

### Genome-wide linkage and association studies

There are essentially two types of study design—genome-wide and candidate gene—that are used to localise the genes underlying human disease. Genome-wide studies have the advantage that no supposition is made about the genes involved, and potentially novel or unconsidered genes may be identified. Typically, the genome-wide approach has used linkage studies of multiply affected (or infected) pedigrees to identify regions of the entire genome that are transmitted from the parents to the offspring more often than expected under independent inheritance.^43^ Typically, affected sibling pairs,^57^, ^58^ or larger multicase pedigrees^59^ if available, are recruited and the inherited regions of the genome are defined by a few hundred microsatellite markers. The main disadvantages of linkage studies are that sufficient numbers of affected sibling pairs may be difficult to recruit for many infections, and that linkage studies are often too insensitive to pick up the relatively small contributions from individual genetic regions that are typical of complex diseases such as infection. There are examples of successful genome scans undertaken using linkage analysis for infectious diseases, including the intensity of infection with *Schistosoma mansoni*,^59^ susceptibility to *H pylori*,^60^ and to leprosy.^57^, ^58^, ^61^ As with association data, some linkage studies have not been replicated in different populations. In some cases this may represent a false discovery rate, an inherent problem in studies that make multiple statistical comparisons, but it is increasingly clear that many complex diseases show genetic heterogeneity, with different genetic determinants for the same disease operating in different ethnic groups. For example, mutations in the *NOD2*/*CARD15* locus, an important susceptibility determinant of inflammatory bowel disease in white populations, are effectively absent in Asian populations with the same phenotype.^62^

Genome-wide association studies, in which the whole genome is interrogated using hundreds of thousands of SNPs, have recently become a reality as genotyping technology and analytical tools evolve. This approach will potentially detect more subtle genetic effects than a classic linkage study.^63^ At present, studies use up to 0·5 million of the approximately 11 million estimated SNPs in the human genome, but there are unresolved issues particularly relating to the optimum density and location for these SNP markers (gene-centric or evenly spaced through the genome), which have implications for how much of the genome such studies truly cover,^45^ as well as concerns regarding sample size and power.^64^ Genome-wide association studies have a high false discovery rate, and replication in independent populations is essential. There are currently no reported genome-wide association studies in infectious diseases, although such studies are underway. In particular, studies being undertaken under the banner of the Wellcome Trust Case-Control Consortium will look at up to 2000 cases and 3000 controls for eight complex diseases, including tuberculosis and malaria.^65^ For these two important infectious diseases, a two-stage strategy is proposed in which a 750 000 SNP chip (panel) will be used to genotype 1000 cases and 1000 controls, and regions positive at p=0·1 followed up in at least another 1000 cases and 1000 controls. This is one of the biggest projects ever undertaken to identify the genetic variations that may predispose people to, or protect them from, complex diseases. The hope is that by identifying these genetic signposts, researchers will be able to understand which people are most at risk, and also produce more effective treatments.

The genome-wide association approach has been used on a smaller scale in other non-infectious genetically complex diseases. For example, genotyping of almost 93 000 SNPs across the genome has identified a region of chromosome 6 associated with myocardial infarction. Further mapping identified a novel functional polymorphism in the lymphotoxin alpha (*LTA*) gene within this region and related functional variants in a ligand of LTA.^66^, ^67^ The odds ratio associated with the *LTA* mutation was 1·78,^67^ an order of magnitude expected for a complex disease. In another study, a 120 000 SNP chip was used to compare 96 cases of age-related macular degeneration with 50 controls to identify a major gene (complement factor H) that determines susceptibility.^68^ A common intronic SNP among the 120 000 SNPs genotyped was strongly associated with disease, with a nominal p value of less than 10^−7^ (after correction for multiple testing), and a genotype relative risk for homozygous individuals of 7·4 (95% CI 2·9–19·0). Re-sequencing revealed a polymorphism in linkage disequilibrium with the risk allele that caused a tyrosine to histidine change at amino acid 402 in the complement factor H protein. The numbers of cases and controls studied was small, increasing the possibility of a false positive result (although the relative risk was high compared with our usual expectation for complex diseases). Importantly, the SNP-chip study was immediately supported by two studies using four independent case-control populations.^69^, ^70^

These studies provide a major conceptual breakthrough in terms of the approaches that can be made with novel technologies, now more feasible with the recent publication of the phase I HapMap,^71^ and the data for phase II released in 2005 (http://www.hapmap.org). The main practical objective of the HapMap project was to identify sets of SNPs that are in linkage disequilibrium with each other, so that a subset of these can be genotyped as so-called haplotype-tagging SNPs in a genome-wide association study, thus reducing the overall amount of genotyping required. The phase I data involved the genotyping of 1 million SNPs in unrelated individuals or parent/child trios of Nigerian, European, Chinese, and Japanese descent. Phase II extends this to 3·5 million SNPs. The phase I data has already provided many predictions and tests of applicability of haplotype tagging across populations,^72^, ^73^ optimum sampling and two-stage testing strategies for whole genome association studies,^74^ and which commercially available SNP chips will work best. As predicted from previous studies, a greater density of SNPs on a chip is required to capture all the haplotypic diversity in the African population compared with European, Chinese, or Japanese populations, because of the greater diversity and shorter regions of linkage disequilibrium observed in the former,^75^ a factor that will be important in application of the technology to infectious disease association studies in Africa. Nevertheless, for 1000 cases and 1000 controls and a nominal p value of 0·01, the phase I HapMap (eg, the newly released Sentrix human Hap300 genotyping beadchip by Illimuna;^51^ one SNP about every 12 kb) retains 77% power to detect associations due to common (≥5%) causal alleles in the African population, compared with roughly 90% in European, Chinese, or Japanese populations.^75^ The 500 000 SNP chip from Affymetrix,^50^ which was made before the release of phase I HapMap haplotype-tagging SNP information, has about the same power as the Illumina human Hap300 chip (ie, it contains about two tags per haplotype).

### Candidate gene approach

Although there will probably be many whole genome association studies in the future, to date most studies of complex diseases, including infection, have adopted a candidate gene approach. The choice of the candidate genes may come from animal data,^76^, ^77^ results of whole-genome studies,^57^, ^58^, ^78^ clinical data,^79^, ^80^ or simply biological plausibility. Important methodological considerations in this approach include the following: (1) an adequate density of the markers in the candidate gene to ensure that the study has sufficient sensitivity to exclude a genetic region; (2) narrow and consistent phenotypic definitions; (3) matching of cases and controls; and (4) sample size and power.

Most candidate gene studies use a case-control approach, and unrecognised ethnic differences between the groups will probably result in spurious genetic associations that are unrelated to the disease of interest. For example, there is probably a strong association between the HLA region and chopstick use in Sydney. This does not imply that the HLA region necessarily influences manual dexterity, but more likely that the HLA type identifies an Asian subpopulation. This issue of population stratification is likely to be more confounded when small genetic effects are sought in large populations.^81^ However, this confounding can be minimised if cases and controls are carefully matched. The presence of genetic admixture can also be quantified from the genetic data itself,^82^ allowing stratified analyses if the sample size permits. In paediatric populations, an alternative approach is to use the parents as the genetic controls for their affected offspring.^83^ This approach is intrinsically less powerful than case-control designs, since the parental controls are enriched for the genetic variants of interest, but it allows inclusion of families of mixed ethnicity and potential pooling of data across ethnic groups, if significant genetic heterogeneity is not present.

Probably the most crucial and overlooked methodological issue is sample size and power. Because the genetic effects from individual genetic variants are usually modest, the genetic model (mode of inheritance) is often unknown, and the frequency of the disease-modifying variant may vary, almost all studies of complex disease genetics to date have been underpowered. Power calculations (figure) indicate that in excess of 1500 case-control pairs or case-parent trios are needed to provide adequate power, especially as effect sizes (odds ratios) expected for a genetic contribution to complex disease susceptibility will probably be small (about 1·5).

**Figure:**
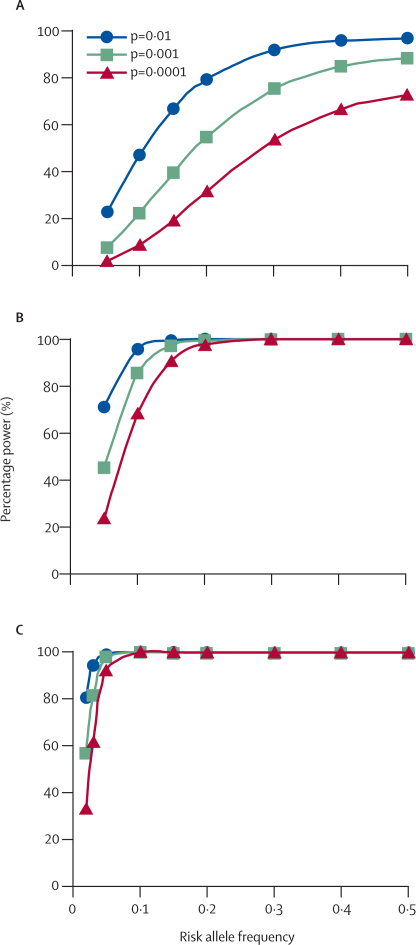
Power to detect allelic association for a risk allele having an effect size (odds ratio) of 1·5 (A) 500, (B) 1500, and (C) 3000 trios or case-control pairs. Power is calculated for different frequencies of risk alleles, and for samples of 500, 1500, or 3000 trios or case-control pairs. Power approximations for trios by a standard transmission disequilibrium test^84^ have been made using the method of Knapp.^85^ Theoretical power to detect allelic association was made assuming a multiplicative model. Results are given as a first approximation of the percentage power to detect allelic association at p=0·01, p=0·001, or p=0·0001. Power calculations were essentially identical for a similar size case-control sample. The advantage of trios is that they are not influenced by population admixture. The advantage of case-control analysis is one third less genotyping to obtain equivalent power. The graphs show that 500 trios or case-control pairs have very poor power to detect allelic association even for relatively common risk alleles (frequencies >0·2). A sample of 1500 trios or case-control pairs has good power for risk alleles at frequencies >0·1. A sample of 3000 trios or case-control pairs improves power for rare risk alleles (frequencies <0·1).

The lack of power owing to small sample sizes makes it difficult to interpret current and previous data on genetic susceptibility to infection. For this reason we have not attempted a critical appraisal or value judgment of past and present studies, but have provided comprehensive tables (webtables 1, 2, 3 and 4) that list all of the studies undertaken to date for the most commonly studied infectious diseases—HIV, mycobacterial infections (leprosy and tuberculosis), malaria, and leishmaniasis. Whereas some loci (eg, HLA class I, class II, and class III alleles, *SLC11A1*) feature repeatedly both within and between diseases, no formal meta-analyses^86^ of these smaller studies have yet been undertaken to determine which may indicate statistically reliable associations. One interesting observation is the number of candidate gene studies that have focused on proteins involved in innate recognition of microbes and first-line defence innate immunity pathways (table 1), underscoring the likely selective pressures that have been exerted by infectious diseases. The key genes that regulate acquired immune responses also feature as candidate genes in multiple infectious diseases (table 2).

**Table 1:** Examples of candidate genes related to innate immunity that are associated with infectious disease susceptibility or outcome

**Gene**	**Function**	Infectious disease associations*
*TNFA*	Pro-inflammatory cytokine	HIV, hepatitis B,^87^ human papilloma virus,^88^, ^89^ meningococcal meningitis,^90^, ^91^ typhoid,^92^ leprosy, tuberculosis, malaria, leishmaniasis, *Helicobacter pylori*^93^, ^94^, ^95^
*IL1A, IL1B*	Pro-inflammatory cytokine	HIV, hepatitis C,^96^ meningococcal meningitis,^97^*H pylori*,^98^, ^99^, ^100^ periodontitis,^101^, ^102^ tuberculosis, malaria
*IL10*	Anti-inflammatory cytokine	HIV, Epstein-Barr virus,^103^, ^104^ herpes zoster virus,^105^ cytomegalovirus,^106^ hepatitis B and C,^107^*H pylori*,^95^, ^99^ meningococcal meningitis,^91^, ^108^ pneumonia^109^ and pneumococcal septic shock,^110^ tuberculosis, leprosy
*NOS2A*	Inducible nitric oxide synthase producing toxic nitrogen radicals	Hepatitis C,^111^ brucellosis,^112^ tuberculosis, malaria
*FcγRIIA*†	Activating Fc receptor promoting pro-inflammatory response	HIV, severe acute respiratory syndrome-associated coronavirus,^113^ dengue haemorrhagic fever,^114^ otitis media,^115^*Streptococcus pneumoniae*,^116^ rheumatic fever (streptococcus),^117^ periodontitis,^118^, ^119^, ^120^, ^121^ meningococcal meningitis and sepsis,^108^, ^122^, ^123^ malaria
*FcγRIIB*†	De-activating Fc receptor down regulating pro-inflammatory response	Periodontitis,^124^ malaria
*SLC11A1*	Proton-coupled divalent cation transporter with multiple pleiotropic effects on macrophage function	HIV, hepatitis C,^125^ leprosy, tuberculosis, Buruli ulcer (*Mycobacterium ulcerans*),^126^ Kawasaki disease,^127^ visceral leishmaniasis
*MBP* (*MBL*)	Mannose binding lectin opsonises for complement activation by classic pathway	HIV, hepatitis B,^128^, ^129^ severe acute respiratory syndrome-associated coronavirus,^130^ Gram negative and positive bacterial infection,^131^ Kawasaki disease,^132^*Chlamydia pneumoniae*,^133^ meningococcal meningitis,^134^ aspergillosis,^135^ candidiasis,^136^ tuberculosis, malaria, filariasis^137^
*TLR2*/*TLR4*	Toll receptors 2 and 4;^138^ pattern recognition receptor for lipopolysaccharide and lipoproteins on bacteria and mycobacteria	Respiratory syncytial virus,^139^ meninogoccal meningitis,^122^, ^140^ Lyme disease (*Borrelia bergdorferi*),^141^ staphylococcal infection,^142^ periodontitis,^143^ Boutonneuse fever,^144^ Gram negative^145^, ^146^ and positive^131^ bacterial infection, brucellosis,^147^ Legionnaires' disease,^148^ tuberculosis, leprosy, malaria

* References given are only for disease associations not covered in webtables 1–4 that summarise candidate gene association studies for HIV, tuberculosis, leprosy, malaria, and leishmaniasis. References are not exhaustive, and generally provide examples of studies showing associations that have been published in the last 5 years.

† These two genes are closely linked and are usually in linkage disequilibrium with each other, making it unclear which of these loci is the disease-associated gene.

**Table 2:** Examples of candidate genes related to acquired immunity that are associated with infectious disease susceptibility or outcome

**Gene**	**Function**	Infectious disease associations*
HLA class I	Presentation of antigen to CD8 T cells	HIV, tuberculosis, leprosy, malaria, leishmaniasis
HLA class II	Presentation of antigen to CD4 T cells	HIV, hepatitis C,^149^ tuberculosis, leprosy, malaria, leishmaniasis
*IL4*/*IL13*	Cytokine product of T-helper 2 cells	HIV, hepatitis C,^150^ respiratory syncytial virus,^151^, ^152^*H pylori*,^153^ candidiasis,^154^ malaria, leishmaniasis, schistosomiasis^155^
*IFNG*†	Cytokine product of T-helper 1 cells	HIV, hepatitis B,^156^ Epstein-Barr virus,^157^ respiratory syncytial virus,^158^, ^159^*H pylori*,^95^ brucellosis,^160^ malaria, tuberculosis, leprosy, schistosomiasis^161^
*IFNGR1*†	Type 1 receptor for interferon γ on macrophages	Periodontitis,^162^*H pylori*,^163^ atypical mycobacterial infection,^164^, ^165^ tuberculosis, malaria, post-Kala-azar dermal leishmaniasis

* References given are only for disease associations not covered in webtables 1–4 that summarise candidate gene association studies for HIV, tuberculosis, leprosy, malaria, and leishmaniasis. References are not exhaustive, and generally provide examples of studies showing associations that have been published in the last 5 years.

† Since natural killer cells make interferon γ, these genes could also be thought of as innate immunity genes.

Most of these studies also suffer from a lack of statistical power, and require replication and validation. of these immune response genes are highly polymorphic or clustered, or both, in the genome, which is an indication of evolutionary pressure and selection.^166^, ^167^ A more formal attempt to search for signals of evolutionary selection was recently undertaken in 168 genes related to immune function.^3^ Almost 1700 common SNPs were successfully genotyped across these genes in three population samples: 96 independent chromosomes from Utah residents with European ancestry, 120 from Han Chinese Guangxi, and 124 from the Yoruba people of Southwest Nigeria (these samples were overlapping but not identical to those used in the HapMap project). Evidence for selection was based on four tests of non-neutral evolution: (1) assessment of the percentage of SNPs within a locus with low (<10%) minor allele frequency and the percentage with high (>40%) minor allele frequency; (2) assessment of the frequency of derived (non-ancestral) alleles; (3) comparison of *F*_ST_ (panel) versus heterozygosity; and (4) relative extended haplotype homozygosity. In tests of allele frequency, three of the 168 genes stood out: *IL9, FUT2*, and *CAV2*. *IL9* lies in the cluster of T-helper-2 related genes encoding interleukins 4, 5, 9, and 13 on chromosome 5q31-q33, which has been implicated in many infectious disease studies (table 2; webtables 1–4). *FUT2* encodes a protein responsible for determining ABO blood protein secretor status, and variation in this gene may protect against respiratory pathogens,^168^ *H pylori*,^169^ and HIV-1.^170^ *CAV2* encodes one of the caveolin proteins that function as scaffolding structures in cholesterol-rich lipid rafts, which enhance immune cell function,^168^, ^171^ and are involved in pathogen entry into host cells.^172^ The relative extended haplotype homozygosity test provided the best evidence for selection, highlighting *ABCC1* (or multidrug resistance protein *MRP1*, implicated in resistance to *Streptococcus pneumoniae* in mice^173^), *APCS* (serum amyloid P component), and *VAV3* (a guanine nucleotide exchange factor expressed in haematopoietic and other cells, recently identified as a gene for type I diabetes in mice^174^) as genes with signatures of past selection. Although Walsh and colleagues^3^ discuss the limitations of their study and the need for validation, their research pilots the search for signatures of evolutionary selection that can now be undertaken in HapMap-style data, providing a real opportunity to identify the genes that have been under the strongest evolutionary pressure from infectious diseases. The advent of all these new technologies and tools offer exciting prospects for future research, allowing us to return to the drawing board in studying human genetic susceptibility to infectious disease. In so doing, it will be interesting to see how many of the candidate gene studies undertaken to date (webtables 1–4) are supported by potentially more powerful genome-wide association studies. Although a case can also be made for simply increasing sample size and power even with a more directed candidate gene approach, it is debatable whether the genes that show significant evidence of selection would have been readily chosen as obvious candidate loci. Thus, a combination of genome-wide, candidate gene, and other approaches may ultimately prove the most rewarding.

## Gene–gene and gene–environment interactions

Genetic determinants obviously act in a genetic and environmental context, a fact often ignored in genetic epidemiological studies that have previously regarded the environment as something of an annoyance. A genetic variant may substantially increase the risk of avian influenza several hundred times, but if the person carrying the variant never encounters H5N1 influenza, or requires an additional polymorphism in a different gene to have a functional effect, then this first variant may be of no clinical relevance. The field of genetic epidemiology is increasingly acknowledging the importance of complex biological interactions in understanding genetic determinants. Gene–gene interactions (epistasis) have been described for HIV infection, in which an HLA class I allele is associated with more rapid progression to AIDS only when an individual also carries a specific natural killer cell (KIR) receptor.^175^ Gene–environment interactions have been shown in spontaneous preterm labour, which has an inflammatory basis. Although genetic variation at the tumour necrosis factor (*TNFA*) gene is associated with increased risk of preterm labour, this risk is increased significantly in the presence of bacterial vaginosis, itself an independent risk for the same outcome.^176^ In cystic fibrosis, those carrying functional variants of mannose-binding lectin (MBL) have worse pulmonary outcomes,^177^ and replacement MBL therapy has been reported in MBL-deficient cystic fibrosis patients.^178^ Not surprisingly, MBL exerts a protective effect through its innate immunological effects and in a recent small study, MBL deficiency only affected lung function when associated with *Staphylococcus aureus* colonisation,^179^ an example of a gene–enviroment interaction.

## Conclusion: translation of genetic data into therapeutic interventions

Despite an increasing body of robust data identifying genetic determinants of infectious diseases and illuminating key biological pathways, the move from bench to bedside in infectious diseases and other fields remains largely an unrealised, but often made, prediction. There are a few worthy exceptions. Functional genetic variation of the P450 cytochrome system seems to mediate the efficacy of *H pylori* eradication by its effects on proton pump inhibitor metabolism.^180^ The most striking and elegant example of pharmacogenetics is in HIV medicine, in which hypersensitivity to the nucleoside reverse transcriptase inhibitor abacavir has previously limited its use. Analysis of the MHC led to the identification of a particular HLA haplotype that greatly increased the risk of abacavir hypersensitivity,^181^ and further mapping localised a functional variant in the peptide-binding groove of heat shock protein 70, which lies in the central MHC.^182^ Genotyping individuals for this variant before commencing abacavir is cost effective.^183^ Hopefully, further examples of clinically relevant pharmacogenetics in infectious diseases will be reported. In the face of increasing antimicrobial resistance, falling pharmaceutical enthusiasm for new antibiotic development, and the disappointing track record of adjunct biological therapies in severe sepsis, the development of novel vaccines and pharmacogenetic interventions remain among the most important goals for genetic studies of infectious disease susceptibility.

## Search strategy and selection criteria

Data for this review were identified by searches of PubMed, MEDLINE, Current Contents, and references from relevant articles; numerous articles were identified through searches of the extensive files of the authors. Search terms included combinations of “susceptibility”, “genetic susceptibility”, “heritability”, “twin”, “genetic”, “genetic epidemiology”, “infection”, “infectious diseases”, as well as terms for specific infections (eg, “malaria”) and genes and gene products (eg, “mannose binding lectin”): eg, PubMed search term: “HIV AND polymorphism NOT drug”; field: text word; limits: humans. No date or language restrictions were set in these searches.
