# Supplementary material for: Genetic susceptibility to infectious diseases: big is beautiful, but will bigger be even better?
Source: Lancet Infect Dis. 2006 Oct;6(10):653–63. doi: 10.1016/S1473-3099(06)70601-6 (PMC2330096; doi:10.1016/S1473-3099(06)70601-6)
Supplement: Supplementary file 1 [file mmc1.pdf]

On-line Supplementary Table 1 - HIV and AIDS

**Papers Reporting Significant Linkage or Association**						
**Candidate Gene**	**Population**	**Phenotype**	**Sample Size**	**Reported Results**	**Year**	**Reference**
**MHC Class I Region:**						
A1-B8-DR3	Caucasian British	HIV-1 Progression (AIDS and Death)	262 Ind	RR = 1.9 - 3.7; p < 0.05	1996	[McNeil, 1996 #373]
A*2301	**N. American?**	HIV-1 Progression	LTNP = 30; RP = 14	Increased in RP; p = 0.0006	1997	[Chen, 1997 #352]
B53	Hispanic American	HIV-1 Infection	HIV-1^{+ve} = 38; EP = 12	OR = 0.08; p = 0.038	1998	[Rohowsky-Kochan, 1998 #520]
Cw7	Hispanic American	HIV-1 Infection	HIV-1^{+ve} = 38; EP = 12	OR = 9.9; p = 0.012	1998	[Rohowsky-Kochan, 1998 #520]
B*35, Cw*04	Caucasian American	HIV-1 Progression	330 Ind	RH = 2.34 & 2.41; p = 0.0001 & 0.00001	1999	[Carrington, 1999 #326]
B*5703	Rwandan	HIV-1 Progression	RP = 15; SP = 101; IP = 86	OR = 0.37; p = 0.02	1999	[Costello, 1999 #316]
B27; B57	Caucasian	HIV-1 Progression (AIDS Free Time)	375 Seroconvertors	RH < 0.54; p < 0.02	1999	[Keet, 1999 #524]
A24	Caucasian	HIV-1 Progression (AIDS Free Time)	375 Seroconvertors	RH = 1.57; p = 0.004	1999	[Keet, 1999 #524]
B14, C8, B27, B57, C14	Caucasian French	HIV-1 Infection	SP = 200; RP = 75	OR = 0.14 - 0.34; p < 0.032	1999	[Hendel, 1999 #345]
A29, B22, B35, C16	Caucasian French	HIV-1 Infection	SP = 200; RP = 75	OR = 1.62 - 14.09; p < 0.042	1999	[Hendel, 1999 #345]
B*5701	**N. American?**	HIV-1 Progression	HIV-1^{+ve} = 200; LTNP = 13	Increased in LTNP; p < 0.001	2000	[Migueles, 2000 #383]
A2/6802 Supertype	Italian	HIV-1 Seroconversion vs HEPS	HIV-1^{+ve} = 122; HEPS = 110	IRR = 0.45; p = 0.0003	2000	[MacDonald, 2000 #535]
A3	Italian	HIV-1 Seroconversion vs HEPS	HIV-1^{+ve} = 122; HEPS = 110	IRR = 1.55; p = 0.02	2000	[MacDonald, 2000 #535]
B8	Australian Caucasian	HIV-1 Infection Following Transfusion	Ca = 20; HC Numbers Not Given	Decreased in Cases; p < 0.05	2000	[Geczy, 2000 #532]
Bw4	**?**	HIV-1 Progression (CD4 Count, Viral Load, AIDS)	**?**	Delayed Progression in Bw4 Homozygosity; ?	2001	[Flores-Villanueva, 2001 #354]
B*35 (-Px)	**N. American (Mixed Ethnicities?)**	HIV-1 Progression (to AIDS)	850 Ind	**More Rapid Progression; ?**	2001	[Gao, 2001 #339]
G?	**?**	VT in Mother-Child Pairs	**?**	Higher Risk of VT in Concordant Mother-Child Pairs	2001	[Aikhionbare, 2001 #545]
B35	Senegalese	HIV-2 Progression	**RP = 26; SP = 41?**	Increased in RP; p < 0.05	2002	[Diouf, 2002 #356]
B57	REACH Cohort (Mixed Ethnicities)	HIV-1 Progression (Viral Load)	227 Ind	Reduced Viral Load; p < 0.0001 (Adj for Ethnicity)	2002	[Tang, 2002 #313]
Bw4-80Ile	N. American (Mixed Ethnicities)	HIV-1 Progression (to AIDS or Death)	1039 Ind	Slower Progression; RH = 0.79 - 0.83; p < 0.05	2002	[Martin, 2002 #338]
A*0205	**?**	HIV-1 Infection	HIV-1^{+ve} = 184; HEPS = 100	OR = 4.45; p = 0.009	2003	[Liu, 2003 #334]
B*35	**?**	HIV-1 Infection	HIV-1^{+ve} = 184; HEPS = 100	OR = 0.29; p = 0.009	2003	[Liu, 2003 #334]
**B?**	Zambian	HIV-1 Transmission in Discordant Couples	125 Initially; 104 Persistantly Discordant	Increased HLA-B allele-sharing; RH = 2.23; p < 0.001	2004	[Dorak, 2004 #319]
B*5401	Japanese	HIV-1 Progression (Survival)	LTNP = 80; ?	OR = 0.22; p = 0.016	2005	[Munkanta, 2005 #430]
B*1507	Japanese	HIV-1 Progression (Survival)	LTNP = 80; ?	OR = 6.40; p = 0.039	2005	[Munkanta, 2005 #430]
**MHC Class II Region:**						
DR4	Australian Caucasian	HIV-1 Progression (CD4 < 200)	139 Transfusion Infected Ind	Decreased in RP; p < 0.05	1992	[Donald, 1992 #500]
DR5, DR3	Australian Caucasian	HIV-1 Progression (CD4 < 200)	139 Transfusion Infected Ind	Increased in RP; p < 0.05	1992	[Donald, 1992 #500]
DRB1-14a, -13a4	Italian	VT in Mother-Child Pairs	HIV-1^{+ve} = 35; HIV-1-ve = 48; HC = 89	**Decreased in Seropositive Babies; p = ?**	1993	[Greggio, 1993 #499]
DQB1*0501-DQA1*0101-DRB1*0101	**N. American?**	HIV-1 Progression (CD4 Count or AIDS)	95 HIV-1^{+ve} Sibling Pairs	**More Rapid Progression;?**	1995	[Kroner, 1995 #350]
DQB1*0302-DQA1*0301-DRB1*0401-DRB4*0101	**N. American?**	HIV-1 Progression (CD4 Count or AIDS)	95 HIV-1^{+ve} Sibling Pairs	**Slower Progression;?**	1995	[Kroner, 1995 #350]
DQB1*0605	African American	HIV-1 Infection	Ca = 98; HC = 30	**Increased Risk of Infection;?**	1996	[Achord, 1996 #320]
DQB1*0602	Caucasian American	HIV-1 Infection	Ca = 143; HC = 22	**Increased Risk of Infection;?**	1996	[Achord, 1996 #320]
DQB1*0603	Caucasian American	HIV-1 Infection	Ca = 143; HC = 22	**Decreased Risk of Infection; ?**	1996	[Achord, 1996 #320]
DRB*1301, 1302, 1303, 1310	**N. American?**	HIV-1 Progression Following VT	LTNP = 30; RP = 14	Increased in LTNP; p = 0.009	1997	[Chen, 1997 #352]
DQ4	African American	HIV-1 Infection	HIV-1^{+ve} = 47; EP = 16	OR = 0.086; p = 0.018	1998	[Rohowsky-Kochan, 1998 #520]
DQ7	African American	HIV-1 Infection	HIV-1^{+ve} = 47; EP = 16	OR = 10.3; p = 0.03	1998	[Rohowsky-Kochan, 1998 #520]
DR11	Caucasian French	HIV-1 Infection	SP = 200; RP = 75	OR > 2.05; p < 0.008	1999	[Hendel, 1999 #345]
DRB1*1200 - DQB1*0301	Caucasian	HIV-1 Progression (AIDS Free Time)	375 Seroconvertors	RH = 1.83; p = 0.04	1999	[Keet, 1999 #524]
DRB1*01	Kenyan	HIV-1 Seroconversion vs HEPS	HIV-1^{+ve} = 122; HEPS = 110	IRR = 0.22; p = 0.0003	2000	[MacDonald, 2000 #535]
DQB1*0203	Argentinian	HIV-1 Infection	HIV-1^{+ve} = 54; HC = 57	Decreased in cases; p = 0.041	2002	[Motta, 2002 #384]
DRB1*01	Argentinian	HIV-1 Infection	HIV-1^{+ve} = 54; HC = 46	Decreased in cases; p = 0.05	2002	[Motta, 2002 #384]
DRB*13	Argentinian	HIV-1 Infection	HIV-1^{+ve} = 54; HC = 46	Increased in cases; p = 0.017	2002	[Motta, 2002 #384]
DQB1*06	British (Mixed Ethnicities)	HIV-1 Porgression (CD4 Count)	LTNP = 46; IP = 87; RP = 26	Increased in LTNP vs IP; p = 0.04	2004	[Vyakarnam, 2004 #481]
DRB1*0301	Zambian	HIV-1 Infection	HIV-1^{+ve} = 433; HIV-1^{-ve} = 151	Increased in HIV-1^{+ve}; p = 0.04	2004	[Tang, 2004 #482]
DQB1*0604	Zambian	HIV-1 Infection	HIV-1^{+ve} = 433; HIV-1^{-ve} = 151	Increased in HIV-1^{-ve}; p = 0.04	2004	[Tang, 2004 #482]
DRB1*1503-DQB1*0602	Zambian	Transmission in HIV-1 Discordant Couples	292 HIV-1 Discordant Couples	Accelerated Seroconversion; RH = 1.67; p = 0.03	2004	[Tang, 2004 #482]
DRB1*0301-DQB1*0201	Zambian	Transmission in HIV-1 Discordant Couples	292 HIV-1 Discordant Couples	Accelerated Seroconversion; RH = 1.60; p = 0.009	2004	[Tang, 2004 #482]
DRB*1301	Zambian	Transmission in HIV-1 Discordant Couples	Trans Couples = 124; Untrans = 151	Delayed Transmission; RH = 0.54; p = 0.05	2004	[Tang, 2004 #482]
**MHC Class III Region:**						
C4A*Q0	Caucasian Australian	HIV-1 Infection	HIV-1^{+ve} = 122; HEPS = 67	Increased in Cases; X^2 = 5.65; p < 0.05	1990	[Cameron, 1990 #366]
TNF (m/sat in region)	British Caucasian	HIV-1 Progression (RP vs LTNP or Co_	LTNP = 24; RP = 20; HIV^{-ve} = 109	OR = 0.1 or 0.2; p = 0.002 or 0.006	1997	[Khoo, 1997 #503]
TNF (-308 - AA)	N. American (Mixed Ethnicities?)	HIV-1 Progression (LTNP vs Blood Donors)	LTNP = 32; BD = 196	Increased in LTNP; p < 0.05	1998	[Knuchel, 1998 #504]
TAP2 (Ala665)	**?**	HIV-1 Infection	HIV-1^{+ve} = 184; HEPS = 100	OR = 2.26; p = 0.002	2003	[Liu, 2003 #334]
TNF (-1030, -862)	**N. American (Mixed Ethnicities?)**	HIV-1 Progression (Viral Load)	**?**	Increased Viral Load; p = 0.03	2003	[Delgado, 2003 #355]
**Chemokines and Chemokine Receptors:**						

On-line Supplementary Table 1 - HIV and AIDS

CCL2 (+113 CT, +459 CT)	African American	HIV-1 Infection	HIV-1^{+ve} = 403; HC = 246	OR = 0.623; p = 0.029	2001	[Gonzalez, 2001 #370]
CCL2 (-2578 GG)	N. American (Mixed Ethnicities)	HIV-1 Infection	HIV-1^{+ve} = 1084; HC = 898	OR = 0.80; p = 0.002 (Adjusted for Ethnicity)	2002	[Gonzalez, 2002 #369]
CCL2 (-2136T, 767G)	Caucasian American	HIV-1 Infection	HIV-1^{+ve} = 600; HEPS = 159	OR = 0.63 & 0.62; p = 0.01 & 0.009	2003	[Modi, 2003 #432]
CCL3L1 (Copy Number)	African Americans	HIV-1 Infection	HIV-1^{+ve} = 409; HC = 497	Low (0-3) Copies ; OR > 1.55; p < 0.03	2005	[Gonzalez, 2005 #368]
CCL3L1 (Copy Number)	Caucasian Americans	HIV-1 Infection	HIV-1^{+ve} = 620; HC = 675	Low (0-1) Copies ; OR > 2.41; p < 0.0007	2005	[Gonzalez, 2005 #368]
CCL3L1 (Copy Number)	Hispanic Americans	HIV-1 Infection	HIV-1^{+ve} = 69; HC = 101	Low (1) Copies ; OR = 4.96; p = 0.0021	2005	[Gonzalez, 2005 #368]
CCL4L2	Spanish	HIV-1 Infection	HIV-1^{+ve} = 175; HC = 220	Increased in HIV^{+ve}; X^2 = 14.24; p = 0.00016	2005	[Colobran, 2005 #492]
CCL4 (R22H)	Caucasian French	HIV-1 Progression	**?**	**Reduced Survival; ?**	2005	[Capoulade-Metay, 2005 #374]
CCL5 (-28G)	Japanese	HIV-1 Progression (CD4 Count)	272 Ind	Slower CD4 Depletion; p = 0.008	1999	[Liu, 1999 #440]
CCL5 (-430G/A, -28C/G)	Caucasian American	HIV-1 Infection vs EU or Blood Donors	HIV-1^{+ve} = 349; EU = 79; BD = 151	OR = 2.11; p = 0.011 (Stratified by D32)	2000	[McDermott, 2000 #516]
CCL5 (-430-GA, -28-CC)	N. American (Mixed Ethnicities)	HIV-1 Progression	404 Ind	RH = 0.65; p = 0.007 (Stratified by D32)	2000	[McDermott, 2000 #516]
CCL5 (-403 AA, -28 CC)	Caucasian Americans	HIV-1 Infection and Progression	HIV-1^{+ve} = 618; HC = 383	OR = 0.78; p = 0.05	2001	[Gonzalez, 2001 #370]
CCL5 (In1.1C)	African American	HIV-1 Progression (to AIDS or Death)	291 Ind	RH = 1.9 & 4.6; p = 0.009 & 0.002	2002	[An, 2002 #389]
CCL5 (-403G, -28C)	Chinese	HIV-1 Infection	Ca = 249; HC = 1082	Increased in Controls; p = 0.048	2004	[Zhao, 2004 #455]
CCL5 (-403A, -28G)	Chinese	HIV-1 Progression	HIV-1^{+ve} Sym = 134; Asym = 115	Increased in Symptomatic Cases; p = 0.007	2004	[Zhao, 2004 #455]
CCL11 (-1385A)	Caucasian American	HIV-1 Infection	HIV-1^{+ve} = 600; HEPS = 159	OR = 0.60; p = 0.005	2003	[Modi, 2003 #432]
CCR5 (D32/D32)	Caucasian American	HIV-1 Infection	HIV-1^{+ve} = 1343; EU = 612	**Increased in EU; ?**	1996	[Dean, 1996 #329]
CCR5 (wt/D32)	Danish	HIV-1 Progression (CD4 Count, Viral RNA)	96 Ind	Slower Progression; p =< 0.005	1997	[Katzenstein, 1997 #359]
CCR5 (D32/D32)	Caucasian American	HIV-1 Infection	HEPS = 111; HIV-1^{+ve} = 614; BD = 387	Increased in HEPS; p < 0.0001	1997	[Zimmerman, 1997 #497]
CCR5 (wt/D32)	Swiss	HIV-1 Progression (LTNP vs Progressors)	**?**	Increased in LTNP; p < 0.0001	1997	[Morawetz, 1997 #506]
CCR5 (wt/D32)	**?American?**	HIV-1 Transmission	54 Heterosexual Couples	Increased in HIV-1 Seronegative Partners; p = 0.03	1997	[Hoffman, 1997 #508]
CCR5 (wt/D32)	Dutch	LTNP vs Normal Progressors	364 HIV-1^{+ve} Ind	OR = 6.9; p < 0.001	1997	[de Roda Husman, 1997 #525]
CCR5 (D32)	Danish	LTNP vs HIV^{-ve} or Fast Progressors	LTNP = 9; RP = 9; HIV^{-ve} = 37	Increased in LTNP & HIV^{-ve}; p < 0.01	1997	[Eugen-Olsen, 1997 #513]
CCR5 (wt/D32)	Caucasian French	HIV-1 Progression(to AIDS)	412 Ind	RR = 0.67; p < 0.02	1997	[Meyer, 1997 #380]
CCR5 (wt/D32)	Norwegian	HIV-1 Progression (to Death)	310 Ind	Slower Progression; p < 0.05	1998	[Eskild, 1998 #357]
CCR5 (P1/P1)	Caucasian American	HIV-1 Progression (to Aids)	700 Ind	RH = 1.53; p = 0.005	1998	[Martin, 1998 #346]
CCR5 (wt/D32)	Swedish	HIV-1 Disease Progresion (SI Virus Type)	258 Ind	Increased Prevalence of AIDS; p < 0.001	1998	[Bratt, 1998 #325]
CCR5 (D32)	Spanish	HIV-1 Infection & Progression	RP = 100; SP = 50; HIV-1^{-ve} = 250	Increased in Controls; p = 0.013 & p = 0.0014	1998	[Alvarez, 1998 #321]
CCR5 (59029-GG)	N. American (Mixed Ethnicities)	HIV-1 Progression to AIDS	418 Ind	RH = 10.4; p = 0.004 (Stratified for D32 & 64I)	1998	[McDermott, 1998 #515]
CCR5 (wt/D32)	British	LTNP vs Blood Donors	**?**	**?Increased in cases;?**	1998	[Balfe, 1998 #322]
CCR5 (59353C)	British Caucasian	HIV-1 Progression (CD4 < 200)	132 HIV-1^{+ve} Ind	HR = 0.58; p = 0.48	1999	[Easterbrook, 1999 #527]
CCR5 (D32/D32)	N. American (Mixed Ethnicities)	VT in Mother-Child Pairs	552 Children of HIV-1^{+ve} Mothers	Increased in Uninfected Children; p < 0.044	1999	[Philpott, 1999 #491]
CCR5 (+.P1.+/+.P1.+)	African American	HIV-1 Progression (CD4 < 200 or AIDS)	149 Ind	RH = 2.30 & 2.31; p = 0.02	2000	[An, 2000 #340]
CCR5 (-2459 AG)	Danish	HIV-1 Progression (to Death)	119 Ind	Slower Progression; p < 0.01 (adj for D32 & 64I)	2001	[Knudsen, 2001 #358]
CCR5 (303A, 627C or HHE)	Argentinian	VT in Mother-Child Pairs	HIV-1^{+ve} = 347; EU = 302	OR = 2.20; p = 0.015	2001	[Mangano, 2001 #519]
CCR5 (-2135T, -2086G)	Japanese	Late Onset AIDS in HIV-1 Infection	98 Ind	OR < 0.502; p < 0.05	2001	[Kageyama, 2001 #546]
CCR5 (P1 Haplotype)	Italian	HIV-1 Progression	73 Ind	More Rapid Disease Progression; p < 0.016	2001	[Ometto, 2001 #536]
CCR5 (59356 CC)	Kenyan	Mortality in HIV-1^{+ve} Mothers	276 Mother-Child Pairs	RH = 3.1; p = 0.05	2001	[John, 2001 #441]
CCR5 (D32)	Italian	HIV-1 Progression	LTNP = 42; HIV-1^{+ve} = 112	Increased in LTNP; p = 0.0434	2001	[Mazzucchelli, 2001 #324]
CCR5 (wt/D32)	N. American (Mixed Ethnicities)	HIV-1 Progression to AIDS in Children	127 Ind	RH = 0.22; p = 0.053	2002	[Sei, 2001 #434]
CCR5 (G*2 - D32)	Caucasian American	HIV-1 Infection	HIV-1^{+ve} = 469; HEPS = 90;	**Increased in HEPS; p = 0.004**	2002	[Tang, 2002 #314]
CCR5 (D32/D32)	Russian	HIV-1 Resistance	HEPS = 74; HC = 163	Increased in HEPS; p < 0.05	2002	[Riabov, 2002 #360]
CCR5 (HHE/HHE)	Caucasian American	HIV-1 Infection	HIV-1^{+ve} = 469; HEPS = 90;	**Decreased in HEPS; p = 0.042**	2002	[Tang, 2002 #314]
CCR5 (A335V)	African plus SA Coloureds	HIV-1 Progression in LTNP vs Rapid or Normal	LTNP = 15; RP = 16; NP = 31	Increased in LTNP; p < 0.0491	2002	[Hayes, 2003 #551]
CCR5 (wt/D32)	N. American (Mixed Ethnicities)	HIV-1 Progression (CD4 Count)	1049 Ind	Increased CD4 Count; p = 0.005 (adj for Ethnicity)	2003	[Singh, 2003 #478]
CCR5 (wt/wt & 59029-AA)	N. American (Mixed Ethnicities)	HIV-1 Progresson (AIDS or Death)	1049 Ind	RH = 1.48; p = 0.009	2003	[Singh, 2003 #478]
CCR5 (wt/D32)	European	HIV-1 Seroconversion to AIDS to Death	1635 Ind	Slower Progression; RH < 0.69; p < 0.003	2003	[Mulherin, 2003 #376]
CCR5 (D32)	German	HIV-1 Progression (Viral Load)	149 Ind	Reduced Viral Load; p = 0.03	2003	[Lichterfeld, 2003 #464]
CCR5 (D32)	N. American (Mixed Ethnicities?)	HIV-1 Progression	**RP = ?; IP = ?**	OR = 0.25; p = 0.007	2004	[Winkler, 2004 #333]
CCR5 (+.PI.+)	N. American (Mixed Ethnicities?)	HIV-1 Progression	**RP = ?; IP = ?**	OR = 2.21; p = 0.01	2004	[Winkler, 2004 #333]
CCR5 (D32/D32)	N. American (Mixed Ethnicities?)	HIV-1 Infection (HEPS vs HIV-1^{+ve})	HEPS = 94; HIV-1^{+ve} = 316	OR = 24.21; p = 0.012	2004	[Liu, 2004 #488]
CCR5 (HHE)	Thai	HIV-1 Progression (CD4 Count < 200)	106 HIV-1^{+ve} IDU	RH = 1.88; p = 0.02	2004	[Nguyen, 2004 #471]
CCR5 (HHE)	Caucasian American & Spanish	HIV-1 Progression (RP vs SP)	RP = 28; SP = 48	Increased in RP; p = 0.002	2005	[Li, 2005 #473]
CCR5 (wt/D32, -2459 AG)	Caucasian American	HEPS vs Low Risk HIV-1^{-ve}	HEPS = 78; HIV-1^{-ve} = 104	Increased in HEPS; p = 0.012	2005	[Hladik, 2005 #493]
CCR2 (wt/64I or 64I/64I)	Caucasian American	HIV-1 Progression (AIDS or Death)	678 Ind	Slower Progression; RH < 0.74; p < 0.04	1997	[Smith, 1997 #330]
CCR2 (64I)	Kenyan	RP vs SP in CSW	RP = 22; SP = 52; HIV-1^{+ve} = 161	RR = 4.17; p = 0.005	1998	[Anzala, 1998 #509]
CCR2 (64I)	African American	AIDS Free Time or Survival	403 Ind	RH = 0.61 or 0.51; p < 0.016	1998	[Mummidi, 1998 #518]
CCR2 (wt/64I)	Caucasian British	HIV-1 Transmission in Discordant Couples	EU = 44; HIV-1^{+ve} = 65	Increased in HIV-1^{+ve} Females; RR = 1.6; p = 0.02	1999	[Lockett, 1999 #344]
CCR2 (64I)	Caucasian British	HIV-1 Progression (CD4 < 200)	132 HIV-1^{+ve} Ind	HR = 0.39; p = 0.03	1999	[Easterbrook, 1999 #527]
CCR2/CCR5 (STRs in Region)	Caucasian American	HIV-1 Progression	410 Ind	p = 0.0009 - 0.05	2000	[Shin, 2000 #530]

On-line Supplementary Table 1 - HIV and AIDS

CCR2/CCR5 (V64I wt/wt, D32 wt/wt, 59029 A/A	Caucasian American	HIV-1 Response to Therapy (Viral RNA Level)	307 Ind	Reduced Viral RNA Level; p = 0.02	2000	[O'Brien, 2000 #341]
CCR2 (64I)	Argentinian	Perinatal HIV-1 Infection	**?**	Increased in Uninfected Children; p = 0.03	2000	[Mangano, 2000 #371]
CCR2 (N260 T)	African plus SA Coloureds	HIV-1 Infection	Ca = 190; HC = 268	Reduced in Cases; p = 0.0255	2002	[Petersen, 2002 #540]
CCR2 (64I)	South African Coloureds	HIV-1 Infection	Ca = 52; HC = 144	Reduced in Cases; p = 0.0034	2002	[Petersen, 2002 #540]
CCR2/CCR5 (HHE/HHE Haplotype)	African American	HIV-1 Progression (Viral RNA Levels)	207 HV-1^{+ve}, AIDS Free Ind	Increased Viral RNA Levels; p = 0.007	2002	[Tang, 2002 #483]
CCR2 (64I)	German	HIV-1 Progression (Viral Load)	149 Ind	Reduced Viral Load; p = 0.015	2003	[Lichterfeld, 2003 #464]
CCR2 (64I)	European & African	HIV-1 Seroconversion to AIDS to Death	Eu = 1635; Af = 215	Slower Progression; RH = 0.42; p < 0.034	2003	[Mulherin, 2003 #376]
CCR2 (64I)	Greek	HIV-1 Response to Therapy (Viral load)	166 Ind	Improved Response; p = 0.0183	2005	[Passam, 2005 #426]
CCR2 (64I)	Cameroon	HIV-1 Infection	**?**	**OR = 6.3; p = ?**	2005	[Ma, 2005 #362]
CX$_3$CR1 (280M/280M)	Caucasian	HIV-1 Progression (CD4 Count or AIDS)	426 Ind	RR = 2.55 or 2.44; p < 0.017 (adj for D32)	2000	[Faure, 2000 #378]
CX$_3$CR1 (249I)	Spanish	HIV-1 Progression	HIV-1^{+ve} = 109; LTNP = 60	OR = 0.46; p = 0.0017	2005	[Vidal, 2005 #429]
CX$_3$CR1 (V249I I/I)	N. American (Mixed Ethnicities)	HIV-1 Progression (AIDS or Death)	1005 Ind	RH = 2.19; p = 0.003	2005	[Singh, 2005 #476]
CXCL12 (3'A/3'A)	Caucasian American	HIV-1 Progression (to AIDS or Death)	639 Ind	RH < 0.37; p_c < 0.004	1998	[Winkler, 1998 #349]
CXCL12 (3'A/3'A)	Dutch	Survival Time After AIDS Diagnosis	344 Ind	RH = 0.40; p = 0.02	1998	[van Rij, 1998 #511]
CXCL12 (wt/3'A)	Kenyan	VT in Mother-Child Pairs	306 Mother-Child Paris	Risk of VT for Maternal wt/3'A; OR = 2.1; p = 0.05	2000	[John, 2000 #442]
CXCL12 (wt/wt)	Italian	Measures of HIV-1 Replication	LTNP = 42 Ind	Decreased Viral Replication; p < 0.01	2001	[Mazzucchelli, 2001 #324]
CXCL12 (3'A)	Italian	HIV-1 Progression	RP = 38; SP = 132; LTNP = 24	Decreased in LTNP; p = 0.0375 & p = 0.0329	2002	[Tresoldi, 2002 #436]
CXCL12 (wt/3'A)	Spanish	HIV-1 LTNP vs EU or Healthy Controls	LTNP = 82; EU = 60; HC = 88	Increased in LTNP; p < 0.005	2002	[Soriano, 2002 #547]
CXCL12 (3'A)	German	HIV-1 Progression (Viral Load)	149 Ind	Reduced Viral Load; p = 0.047	2003	[Lichterfeld, 2003 #464]
CXCL12 (3'A/3'A)	Thai	Resistance to HIV-1 in HEPS CSW	**?**	Increased in HEPS; p < 0.005)	2004	[Tiensiwakul, 2004 #486]
CXCL12 (rs754618)	Caucasian American	HIV-1 Infection	**?**	OR = 1.50; p = 0.03	2005	[Modi, 2005 #431]
CXCL12 (3'A)	Greek	HIV-1 Response to Therapy (CD4 Count)	166 Ind	Improved Response; p = 0.0238	2005	[Passam, 2005 #426]
CXCL12 (3'A)	Caucasian American	HIV-1 Progression	**?**	OR = 0.63; p = 0.01	2005	[Modi, 2005 #431]
**Other Candidates:**						
APOBEC3G (186R)	African American	HIV-1 Progression (Aids or Death)	292 Ind	RH > 1.94; p < 0.024	2004	[An, 2004 #466]
DC-SIGNR (Intron 5 SNP)	Japanese	HIV-1 Progression (CD4 Count)	59 Ind	Lower CD4; p = 0.0087	2002	[Kobayashi, 2002 #447]
DC-SIGN (Neck Region Repeat Domain)	N. American (Mixed Ethnicities?)	HIV-1 Infection (HEPS vs HIV-1^{+ve})	HEPS = 94; HIV-1^{+ve} = 316	OR = 25.03; p = 0.011	2004	[Liu, 2004 #488]
DC-SIGN (-336C)	Caucasian American	HIV-1 Parenteral Infection	HIV-1^{+ve} = 348; HIV-1^{-ve} = 365	OR = 1.87; p_c = 0.005	2004	[Martin, 2004 #387]
DEFB1 (-44 CC)	Italian	HIV-1 Infection	HIV-1^{+ve} = 97; HC = 120	OR = 3.6; p < 0.05	2002	[Braida, 2004 #459]
FcγRIIa (131H/H)	Kenyan	VT in Mother-Child Pairs	448 Mother-Child Pairs	Increased in HIV-1^{+ve} Babies; OR = 2.22; p = 0.009	2004	[Brouwer, 2004 #594]
GNB3 (825TT)	German	HIV-1 Response to Therapy (CD4 Count)	55 Ind	Reduced CD4 Counts; p = 0.047	2005	[Brockmeyer, 2005 #425]
Haptoglobin (Hp2-2)	European Caucasian	HIV-1 Progression	653 Ind	AMRR = 1.78; p = 0.0001	1998	[Delanghe, 1998 #510]
Haptoglobin (Hp0)	Ghanaian	HIV-1 Infection	Ca = 58; HC = 79	OR = 0.21; p = 0.0002	2000	[Quaye, 2000 #534]
Haptoglobin (Hp2-2)	Ghanaian	HIV-1 Progression - CD4 Counts	48 HIV-1^{+ve} Ind	Reduced CD4 Counts; p < 0.25	2000	[Quaye, 2000 #533]
IFNG (-179G)	African American	HIV-Progression (CD4 < 200 or AIDS)	298 Ind	RH = 2.31 & 2.47; p = 0.03 & 0.006	2003	[An, 2003 #465]
IL1A (-889, +4945)	Caucasian Australian	HIV-1 Response to Therapy (Viral RNA Level)	81 Ind	Increased Viral RNA Levels; p = 0.007	2004	[Price, 2004 #393]
IL1RA (N*2/N*2)	Brazilian	Measures of HIV-1 Replication	83 Ind	Replication Reduced; p = 0.01	2001	[Witkin, 2001 #541]
IL10 (H10 Haplotype - 46A)	Caucasian	HIV-1 Infection or Progression	SP = 253; RP = 84	RR > 9.0; p < 0.05	2003	[Vasilescu, 2003 #397]
IL12B (3'UTR, Promoter SNP)	Caucasian Australian	HIV-1 Response to Therapy (Viral RNA Levels)	81 Ind	Increased Viral RNA Levels; p < 0.03	2004	[Price, 2004 #393]
IL4 (-589 TT)	Japanese	HIV-1 Progression (SI Viral Variants)	339 Ind	Increased SI Variants; p = 0.0091	2000	[Nakayama, 2000 #443]
IL4 (-589T)	French	HIV-1 Progression (to AIDS)	148 Ind	Delayed Progression to AIDS; RR = 0.65; p = 0.04	2002	[Nakayama, 2002 #437]
IL4 (-589T)	French	HIV-1 Progression (Viral Load)	400 Ind	Reduced Early Viral Load; p = 0.02	2002	[Nakayama, 2002 #437]
IL4 (589 CC)	Dutch	HIV-1 Progression (SI Viral Variants - X4)	279 Ind	Increased SI Variants; p = 0.01	2003	[Kwa, 2003 #449]
IL4 (549T, 12200T, 12201G, 12198A)	Caucasian	HIV-1 Progression	SP = 253; RP = 84	RR = 1.9; p = 0.016	2003	[Vasilescu, 2004 #396]
IL6 (-174 GG)	Caucasian Australian	HIV-1 Response to Therapy (Viral RNA Levels)	81 Ind	Increased Viral RNA Levels; p = 0.03	2004	[Price, 2004 #393]
IL10 (STRs in Region)	American Caucasian	HIV-1 Infection	Ca = 420; HC = 90 or 410 HIV-1+ve	p = 0.03; p = 0.02_{pc}	2000	[Shin, 2000 #530]
IL10 (-592A)	American Caucasian	HIV-1 Seroconversion to AIDS to Death	769 HIV-1 Seroconvertors	Accelerated Progression; p = 0.0009 - 0.05	2000	[Shin, 2000 #530]
KIR3DS1 (in Absence of HLA-Bw4-80Ile)	N. American (Mixed Ethnicities)	HIV-1 Progression (to AIDS or Death)	1039 Ind	More Rapid Progression; RH = 1.31 - 1.86; p < 0.05	2002	[Martin, 2002 #338]
KIR3DS1 (in Presence of HLA-Bw4-80Ile)	N. American (Mixed Ethnicities)	HIV-1 Progression (to AIDS or Death)	1039 Ind	Slower Progression; RH = 0.598 - 0.65; p < 0.04	2002	[Martin, 2002 #338]
KIR3DS1 (No Bw4-80Ile Interaction Observed)	Caucasian Australian	HIV-1 Progression (CD4 Count)	249 Ind	More Rapid Decline; p = 0.01	**?**	[Gaudieri, 2005 #364]
MBP (Variant Alleles)	Dutch	HIV-1 Seroconversion to AIDS (CD4 Count)	131 HIV-1 Seroconvertors	Lower CD4 Count; p = 0.03	1998	[Maas, 1998 #522]
MBP (Codon 54)	Italian	HIV-1 Progression Following VT	RP = 25; SP = 27; EU = 27; HC = 41	Increased in RP; p < 0.0247	1999	[Amoroso, 1999 #461]
MBP (-550)	Italian	VT in Mother-Child Pairs	HIV-1^{+ve} = 90; EU = 27	Increased in EU; p = 0.0214	2000	[Boniotto, 2000 #460]
MBP (-221)	Italian	HIV-1 Infection (VT) and Progression	RP = 22; SP = 58; EU = 27; HC = 74	RH = 0.09; p = 0.0003	2000	[Boniotto, 2000 #460]
MDR1 (-3435 C/T)	N. American (Mixed Ethnicities)	Response to HAART in HIV-1^{+ve}	71 Ind	More Rapid Response; p < 0.04	2005	[Saitoh, 2005 #477]
SERPINA1 (M2)	South African Coloureds	HIV-1 Infection	Ca = 55; HC = 90	OR = 2.29; p = 0.017	2003	[Hayes, 2003 #551]
SERPINA1 (A332A)	African	HIV-1 Infection	Ca = 177; HC = 155	OR = 4.0; p = 0.037	2003	[Hayes, 2003 #551]
SLC11A1 (GT$_{(n)}$, 274C/T, 469+14G/C)	Colombian	HIV-1 Infection	Ca = 182; HC = 135	RR < 0.35; p < 0.02	1999	[Marquet, 1999 #528]
SLC11A1 (823C/T)	Colombian	HIV-1 Infection	Ca = 182; HC = 135	RR = 2.29; p = 0.035	1999	[Marquet, 1999 #528]

On-line Supplementary Table 1 - HIV and AIDS

**Papers Reporting No Significant Linkage or Association**						
**Candidate Gene**	**Population**	**Phenotype**	**Sample Size**	**Reported Results**	**Year**	**Reference**
**MHC Class I Region:**						
A3, 32, 31	African American	HIV-1 Infection	HIV-1^{+ve} = 47; EP = 16	ns	1998	[Rohowsky-Kochan, 1998 #520]
A24, 31, 33	Hispanic American	HIV-1 Infection	HIV-1^{+ve} = 38; EP = 12	ns	1998	[Rohowsky-Kochan, 1998 #520]
A2, 24, 28	Caucasian American	HIV-1 Infection	HIV-1^{+ve} = 18; EP = 13	ns	1998	[Rohowsky-Kochan, 1998 #520]
B8, 52, 61, 35, 53	African American	HIV-1 Infection	HIV-1^{+ve} = 47; EP = 16	ns	1998	[Rohowsky-Kochan, 1998 #520]
B44, 58, 61, 8	Hispanic American	HIV-1 Infection	HIV-1^{+ve} = 38; EP = 12	ns	1998	[Rohowsky-Kochan, 1998 #520]
B8, 44	Caucasian American	HIV-1 Infection	HIV-1^{+ve} = 18; EP = 13	ns	1998	[Rohowsky-Kochan, 1998 #520]
Cw1, 5, 7, 4, 6, 8	African American	HIV-1 Infection	HIV-1^{+ve} = 47; EP = 16	ns	1998	[Rohowsky-Kochan, 1998 #520]
Cw4, 8	Hispanic American	HIV-1 Infection	HIV-1^{+ve} = 38; EP = 12	ns	1998	[Rohowsky-Kochan, 1998 #520]
Cw4, 7, 8	Caucasian American	HIV-1 Infection	HIV-1^{+ve} = 18; EP = 13	ns	1998	[Rohowsky-Kochan, 1998 #520]
B*4415 & Cw*0407	Kenyan	HIV-1 Infected CSW	HIV-1^{+ve} = 141; HEPS = 92	ns	2002	[Bird, 2002 #548]
**MHC Class II Region:**						
DR1, 3, 11, 13, 12, 17, 18	African American	HIV-1 Infection	HIV-1^{+ve} = 47; EP = 16	ns	1998	[Rohowsky-Kochan, 1998 #520]
DR3, 8, 13, 14, 4, 11, 17, 18	Hispanic American	HIV-1 Infection	HIV-1^{+ve} = 38; EP = 12	ns	1998	[Rohowsky-Kochan, 1998 #520]
DR3, 17, 18, 4, 11, 12	Caucasian American	HIV-1 Infection	HIV-1^{+ve} = 18; EP = 13	ns	1998	[Rohowsky-Kochan, 1998 #520]
DQ6	African American	HIV-1 Infection	HIV-1^{+ve} = 47; EP = 16	ns	1998	[Rohowsky-Kochan, 1998 #520]
DQ6, 7	Caucasian American	HIV-1 Infection	HIV-1^{+ve} = 18; EP = 13	ns	1998	[Rohowsky-Kochan, 1998 #520]
**MHC Class III Region:**						
TNF (-376, -308, -238, -163)	Dutch	HIV-1 Progression	**?**	ns	1997	[Brinkman, 1997 #498]
LTA (STR in Region)	American Caucasian	HIV-1 Infection or Progression	Ca = 420; HC = 90 or 410 HIV-1+ve	p = 0.28; p = 0.19	2000	[Shin, 2000 #530]
MICA	**N. American (Mixed Ethnicities?)**	HIV-1 Progression (Viral Load)	**?**	ns	2003	[Delgado, 2003 #355]
TNF (-308)	Caucasian Australian	HIV-1 Response to Therapy (Viral RNA Levels)	81 Ind	p = 0.26	2004	[Price, 2004 #393]
**Chemokines and Chemokine Receptors:**						
CCL2 (-2136T, 767G)	African American	HIV-1 Infection	HIV-1^{+ve} = 225; HEPS = 88	OR > 0.69; p > 0.34	2003	[Modi, 2003 #432]
CCL3	Japanese	HIV-1 Infection	**?**	ns	2001	[Xin, 2001 #439]
CCL3L1	Caucasian Australian	HIV-1 Infection or Progression	HIV-1^{+ve} = 268; HC = 260	ns	2004	[Bugeja, 2004 #412]
CCL5 (-403, -28)	Japanese	HIV-1 Infection	Ca = 272; HC = 193	ns	1999	[Liu, 1999 #440]
CCL5 (-28)	Japanese	HIV-1 Progression (SI Viral Variants)	339 Ind	p = 0.74	2000	[Nakayama, 2000 #443]
CCL5 (-403, -28)	N. American (Mixed Ethnicities)	HIV-1 Infection	HIV-1^{+ve} = 316; HEPS = 94; HC = 425	ns	2004	[Liu, 2004 #488]
CCL5 (-403G/A, -109T/C, -28C/G)	Spanish	HIV-1 Progression	HIV-1^{+ve} = 109; LTNP = 58	ns	2006	[Vidal, 2006 #428]
CCL11 (-1385A)	African American	HIV-1 Infection	HIV-1^{+ve} = 225; HEPS = 88	OR = 0.55; p = 0.23	2003	[Modi, 2003 #432]
CCR2 (64I)	Danish	HIV-1 Infection or Progression	**HIV-1^{+ve} = 215; EU = ?; BD = ?**	p > 0.06	1998	[Eugen-Olsen, 1998 #514]
CCR2 (64I)	Caucasian American	Aids Free Time or Survival	589 Ind	RH = 0.67; p = 0.91	1998	[Mummidi, 1998 #518]
CCR2/CCR5 (STRs in Region)	American Caucasian	HIV-1 Infection	Ca = 420; HC = 90	p > 0.06	2000	[Shin, 2000 #530]
CCR2 (64I)	Italian	HIV-1 Progression and Viral Load	LTNP = 42; HIV-1^{+ve} = 112	ns	2001	[Mazzucchelli, 2001 #324]
CCR2 (64I)	Ivorian	HIV-1 Infection in CSW	HIV-1^{+ve} = 260; HEPS = 99	ns	2001	[Adje, 2001 #435]
CCR2 (64I)	Spanish	HIV-1 Infection	Ca = 440; HC = 100	ns	2001	[Royo, 2001 #444]
CCR2 (64I)	Luxembourg	HIV-1 Infection	Ca = 288; HC = 158	ns	2002	[Roman, 2002 #542]
CCR2 (64I)	N. American (Mixed Ethnicities)	HIV-1 Progression (CD4 Count or Viral RNA)	1049 Ind	ns	2003	[Singh, 2003 #478]
CCR2 (64I)	N. American (Mixed Ethnicities)	HIV-1 Infection	HIV-1+ve = 316; HEPS = 94; HC = 425	ns	2004	[Liu, 2004 #488]
CCR2 (64I)	N. American (Mixed Ethnicities)	HIV-1 Infection and Progression	HIV-1^{+ve} = 2047; HC = 559	OR = 1.06; p > 0.05 (Stratified for Ethnicity)	2004	[Philpott, 2004 #490]
CCR2 (64I)	Kenyan	VT in Mother-Child Pairs	445 Mother-Child Pairs	ns	2005	[Brouwer, 2005 #474]
CCR5 (D32)	Taiwanese	HIV-1 Infection	HIV-1^{+ve} = 24; HEPS = 131; HC = 187	**ns**	1997	[Li, 1997 #353]
CCR5 (D32)	Italian	HIV-1 Infection (Macrophage Tropic HIV-1)	Ca = 152; HC = 122	ns	1997	[Balotta, 1997 #323]
CCR5 (D35)	Danish	HIV-Infection	Ca = 99; HEPS = 35; HIV^{-ve} = 37	ns	1997	[Eugen-Olsen, 1997 #513]
CCR5 (D32)	Caucasian British	HIV-1 Transmission in Discordant Couples	EU = 51; HIV-1^{+ve} = 70; HC = 48	ns	1999	[Lockett, 1999 #344]
CCR5 (wt/D32)	Italian	VT in Mother-Child Pairs	HIV^{+ve} = 90; HIV^{-ve} = 307	No Protection Against VT	1999	[Ometto, 1999 #537]
CCR5 (D32)	Argentinian	Perinatal HIV-1 Infection	**?**	ns	2000	[Mangano, 2000 #371]
CCR5 (59353, 59356, 59402, 59029)	Kenyan	VT in Mother-Child Pairs	276 Mother-Child Pairs	ns	2001	[John, 2001 #441]
CCR5 (-894C)	Chinese	HIV-1 Infection	**?**	ns	2002	[Liu, 2002 #450]
CCR5 (Codon 35)	African	HIV-1 Infection	Ca = 82; HC = 96	p = 0.2511	2002	[Hayes, 2002 #539]
CCR5 (D32, 58755, m303, 59029, 59653)	Luxembourg	HIV-1 Infection	Ca = 288; HC = 158	ns	2002	[Roman, 2002 #542]
CCR5 (Human Haplogroups)	Thai	HIV-1 Infection	HIV-1^{+ve} = 10; HEPS = 12; HIV-1^{-ve} = 9	ns	2003	[Yang, 2003 #470]
CCR5 (G106R, D32, R223Q, S336I, 299FS)	Chinese	HIV-1 Infection	HIV-1+ve = 314; HC = 785	ns	2005	[Zhao, 2005 #462]
CCR5 (D32, 59029A/G)	Greek	HIV-1 Response to Therapy (Viral Load or CD4)	166 Ind	ns	2005	[Passam, 2005 #426]
CX$_3$CR1 (V249I, T280M)	American Caucasian	HIV-1 Infection vs Exposed, Uninfected	HIV-1^{+ve} = 573; EU = 109	p = 0.72 - 0.82	2000	[McDermott, 2000 #517]
CX$_3$CR1	Caucasian	HIV-1 Porgression	RP = 80; LTNP = 244	p > 0.80	2001	[Hendel, 2001 #399]

On-line Supplementary Table 1 - HIV and AIDS

CX$_3$CR1 (V249I, T280M)	Luxembourg	HIV-1 Infection	Ca = 288; HC = 158	ns	2002	[Roman, 2002 #542]
CXCL12 (3'A)	N. American (Mixed Ethnicities)	HIV-1 Progression	1090 Ind	RH = 1.9; p = 0.014 Adjsuted For Race	1998	[Mummidi, 1998 #518]
CXCL12 (3'A)	Argentinian	Perinatal HIV-1 Infection	**?**	ns	2000	[Mangano, 2000 #371]
CXCL12 (3'A)	Caucasian American	HIV-1 Response to Therapy	307 Ind	ns	2000	[O'Brien, 2000 #341]
CXCL12 (3'A)	Spanish	HIV-1 Infection	Ca = 440; HC = 100	ns	2001	[Royo, 2001 #444]
CXCL12 (3'A)	Italian	HIV-1 Infection	HIV-1+ve = 256; eu = 118; HC = 170	ns	2002	[Tresoldi, 2002 #436]
CXCL12 (3'A)	Luxembourg	HIV-1 Infection	Ca = 288; HC = 158	ns	2002	[Roman, 2002 #542]
CXCL12 (3'A)	N. American (Mixed Ethnicities)	HIV-1 Progression (CD4 Count or Viral RNA)	1049 Ind	ns	2003	[Singh, 2003 #478]
CXCL12 (3'A)	Brazilian	HIV-1 Progression (SI Viral Variants)	Ca = 62; HC = 60	ns	2003	[Watanabe, 2003 #467]
CXCL12 (3'A)	N. American (Mixed Ethnicities)	HIV-1 Infection	HIV-1+ve = 316; HEPS = 94; HC = 425	ns	2004	[Liu, 2004 #488]
CXCL12 (3'A)	Spanish	HIV-1 Progression	HIV-1^{+ve} = 107; LTNP = 57	ns	2005	[Vidal, 2005 #427]
**Other Candidates:**						
APOBEC3G (186R)	Caucasian American	HIV-1 Progression (Aids or Death)	673 Ind	ns	2004	[An, 2004 #466]
APOBEC3G	Caucasian French	HIV-1 Infection	HIV-1^{+ve} = 327; HC = 446	ns	2005	[Do, 2005 #395]
CD30 (Promoter m/sat)	Caucasian Australian	HIV-1 Infection	HIV-1^{+ve} = 112; HC = 100	ns	2003	[McIntyre, 2003 #394]
CD4 (STR in Region)	American Caucasian	HIV-1 Infection or Progression	Ca = 420; HC = 90 or 410 HIV-1+ve	p = 0.27; p = 0.10	2000	[Shin, 2000 #530]
DC-SIGN (-336C)	Caucasian American	HIV-1 Mucosal Infection	HIV-1^{+ve} = 719; HIV-1^{-ve} = 179	p = 0.46	2004	[Martin, 2004 #387]
DC-SIGNR (Exon 4 Repeat)	German	HIV-1 Infection or Progression	HIV-1+ve = 391; HC = 134 or 149 Ind	ns	2003	[Lichterfeld, 2003 #464]
CR1	French	HIV-1 Infection	Ca = 79; HC = 84	ns	1989	[Cohen, 1989 #501]
NR2F6/ERBAL2 (STR in Region)	American Caucasian	HIV-1 Infection or Progression	Ca = 420; HC = 90 or 410 HIV-1+ve	p = 0.38; p = 0.13	2000	[Shin, 2000 #530]
FAS	Caucasian French	HIV-1 Infection	HIV-1^{+ve} = 212; HC = 155	ns	2004	[Vasilescu, 2004 #396]
FASLG	Caucasian French	HIV-1 Infection	HIV-1^{+ve} = 212; HC = 155	ns	2004	[Vasilescu, 2004 #396]
GC	Dutch	HIV-1 Infection or Progression	EU = 351; AIDS = 96; HC = 86	ns	1988	[Pronk, 1988 #502]
GC (STR in Region)	American Caucasian	HIV-1 Infection or Progression	Ca = 420; HC = 90 or 410 HIV-1+ve	p = 0.36; p = 0.14	2000	[Shin, 2000 #530]
IFNA (STR in Region)	American Caucasian	HIV-1 Infection or Progression	Ca = 420; HC = 90 or 410 HIV-1+ve	p = 0.69; p = 0.34	2000	[Shin, 2000 #530]
IFNG (STR in Region)	American Caucasian	HIV-1 Infection or Progression	Ca = 420; HC = 90 or 410 HIV-1+ve	p = 0.82; p = 0.19	2000	[Shin, 2000 #530]
IFNG (CA$_{(n)}$)	American Caucasian	HIV-1 Infection or Progression	**?**	ns	2000	[Bream, 2000 #335]
IFNG (-176G/T)	African American	HIV-1 Infection	HIV-1^{+ve} = 298; HEPS = 78; HIV-1^{-ve} = 385	ns	2003	[An, 2003 #465]
IFNG (X7 SNPs)	Caucasian	HIV-1 Infection or Progression	SP = 253; RP = 84; HC = 470	ns	2003	[Vasilescu, 2003 #397]
IL1A (STR in Region)	American Caucasian	HIV-1 Infection or Progression	Ca = 420; HC = 90 or 410 HIV-1+ve	p = 0.65; p = 0.14	2000	[Shin, 2000 #530]
IL1B (+3935)	Caucasian Australian	HIV-1 Response to Therapy (Viral RNA Levels)	81 Ind	ns	2004	[Price, 2004 #393]
IL1RA (STR in Region)	American Caucasian	HIV-1 Infection or Progression	Ca = 420; HC = 90 or 410 HIV-1+ve	p = 0.39; p = 0.15	2000	[Shin, 2000 #530]
IL12p35 (X9 SNPs)	Caucasian	HIV-1 Infection or Progression	SP = 253; RP = 84; HC = 470	ns	2003	[Vasilescu, 2003 #397]
IL12p40 (X9 SNPs)	Caucasian	HIV-1 Infection or Progression	SP = 253; RP = 84; HC = 470	ns	2003	[Vasilescu, 2003 #397]
IL13 (11842, 776, 816)	Caucasian	HIV-1 Infection or Progression	SP = 253; RP = 84; HC = 470	ns	2003	[Vasilescu, 2003 #397]
IL2 (STR in Region)	American Caucasian	HIV-1 Infection or Progression	Ca = 420; HC = 90 or 410 HIV-1+ve	p = 0.44; p = 0.11	2000	[Shin, 2000 #530]
IL2 (12196, 12193, 376)	Caucasian	HIV-1 Infection or Progression	SP = 253; RP = 84; HC = 470	ns	2003	[Vasilescu, 2003 #397]
IL2RB (STR in Region)	American Caucasian	HIV-1 Infection or Progression	Ca = 420; HC = 90 or 410 HIV-1+ve	p = 0.33; p = 0.53	2000	[Shin, 2000 #530]
IL4 (-589C/T)	Dutch	HIV-1 Progression to AIDS	342 Ind	ns	2003	[Kwa, 2003 #449]
IL4 (-589C/T)	Caucasian American	HIV-1 Infection or Progression	**?**	ns	2003	[Modi, 2003 #433]
IL4 (-589C/T)	African American	HIV-1 Infection or Progression	**?**	ns	2003	[Modi, 2003 #433]
IL4 (-589C/T)	N. American (Mixed Ethnicities)	HIV-1 Progression (CD4 Count, Disease etc)	1043 Ind	ns	2004	[Singh, 2004 #475]
IL5RA (STR in Region)	American Caucasian	HIV-1 Infection or Progression	Ca = 420; HC = 90 or 410 HIV-1+ve	p = 0.33; p = 0.48	2000	[Shin, 2000 #530]
IL6 (X14 SNPs)	Caucasian	HIV-1 Infection or Progression	SP = 253; RP = 84; HC = 470	ns	2003	[Vasilescu, 2003 #397]
IL9 (STR in Region)	American Caucasian	HIV-1 Infection or Progression	Ca = 420; HC = 90 or 410 HIV-1+ve	p = 0.97; p = 0.21	2000	[Shin, 2000 #530]
IL10 (-1087, -824, -597)	Japanese	HIV-1 Progression (SI Viral Variants)	339 Ind	p > 0.05	2000	[Nakayama, 2000 #443]
IL16	Japanese	HIV-1 Infection	**?**	ns	2000	[Nakayama, 2000 #438]
IL16	Thai	HIV-1 Infection	**?**	ns	2000	[Nakayama, 2000 #438]
MBP (Codon 54)	Italian	VT in Mother-Child Pairs	HIV-1+ve = 52; HIV-1-ve = 27; HC = 41	ns	1999	[Amoroso, 1999 #461]
MBP	Colombian	HIV-1 Infection	Ca = 278; HC = 278	p = 0.10	2003	[Malik, 2003 #463]
MDR1 (T129C, G2677T, C3435T)	Caucasian Canadian	HIV-1 Infection	HIV-1^{+ve} = 70; HEPS = 67	ns	2002	[Ifergan, 2002 #456]
MDR1 (G2677T, C3435T)	**N. American (Mixed Ethnicities?)**	Response to Anti-Viral Therapy	31 Ind	ns	2003	[Haas, 2003 #468]
MDR1 (G2677T, C3435T)	Swiss (Mixed Ethnicities)	HIV-1 Progression (CD4 Count)	Ca = 411; HC = 128	ns	2004	[Bleiber, 2004 #479]
MMP7 (-181A/G, -153C/T)	Italian	HIV-1 Infection & Response to Therapy	**Ca = 132; HC = ?**	ns	2005	[Lugli, 2005 #494]
NOS2A (CCTTT$_{(n)}$)	Swiss	HIV-1 Infection or Progression	HIV-1+ve = 857; HC = 240	ns	2004	[Hersberger, 2004 #489]
PFC (STR in Region)	American Caucasian	HIV-1 Infection or Progression	Ca = 420; HC = 90 or 410 HIV-1+ve	p = 0.10; p = 0.41	2000	[Shin, 2000 #530]
SLC11A1 (GT(n), 332C/T)	African & SA Coloureds	HIV-1 Infection	Ca = 84; HC = 133	ns	2004	[Donninger, 2004 #495]
TBP (STR in Region)	American Caucasian	HIV-1 Infection or Progression	Ca = 420; HC = 90 or 410 HIV-1+ve	p = 0.07; p = 0.17	2000	[Shin, 2000 #530]
Theta-Defensin Pseudogene (DEFT)	Thai	HIV-1 Infection	HIV-1^{+ve} = 10; HEPS = 11; HIV-1^{-ve} = 9	ns	2005	[Yang, 2005 #472]

On-line Supplementary Table 1 - HIV and AIDS

**PUBMED Search Term = HIV AND polymorphism NOT drug; Field: Text Word, Limits: Humans**						
Ca = Cases						
Co = Controls						
Ind = Individuals						
ns = Not Significant						
OR = Odds Ratio						
RR = Relative Risk						
χ^2 = Chi-Squared						
ZMLB = Z Score of the Maximum-Likelihood-Binomial						
LOD = Logarithm of the Odds						
MLB-LOD = Maximum-Likelihood-Binomial Logarithm of the Odds						
pc = Corrected p-Value						
nc = Not Corrected						
N/A = Not Available (Possibly Abstract Only Available)						
RH = Relative Hazard						
HR = Hazard Ratio						
HC = Healthy Controls						
BD = Blood Donors						
Eu = European						
Af = African						
VT = Vertical Transmission						
HEPS = Highly Exposed, Persistantly Seronegative						
CSW = Commercial Sex Worker						
LTNP = Long Term Non-Progressor						
EU = Exposed But Uninfected						
RP = Rapid Progressor						
SP = Slow Progressor						
IP = Intermediate Progressor						
NP = Normal Progressor						
EP = Exposed Sexual Partner						
AMRR = Adjusted Mortality Risk Ratio						
IDU = Injection Drug Users						
Sym = Symptomatic						
Asym = Asymptomatic						
(N)SI = (Non)Syncitia Inducing						

Bibliography for Webtable 1.

Achord AP, Lewis RE, Brackin MN, Henderson H, Cruse JM (1996) HIV-1 disease association with HLA-DQ antigens in African Americans and Caucasians. Pathobiology 64:204-8

Adje CA, Bile CE, Kestens L, Koblavi-Deme S, Ghys PD, Maurice C, Kalou-Badirou M, Kabran N, Ekpini RE, Roels TH, Wiktor SZ, Nkengasong JN (2001) Lack of effect of chemokine receptor CCR2b gene polymorphism (64I) on HIV-1 plasma RNA viral load and immune activation among HIV-1 seropositive female workers in Abidjan, Cote d'Ivoire. J Med Virol 64:398-401

Aikhionbare FO, Hodge T, Kuhn L, Bulterys M, Abrams EJ, Bond VC (2001) Mother-to-child discordance in HLA-G exon 2 is associated with a reduced risk of perinatal HIV-1 transmission. Aids 15:2196-8

Alvarez V, Lopez-Larrea C, Coto E (1998) Mutational analysis of the CCR5 and CXCR4 genes (HIV-1 co-receptors) in resistance to HIV-1 infection and AIDS development among intravenous drug users. Hum Genet 102:483-6

Amoroso A, Berrino M, Boniotto M, Crovella S, Palomba E, Scarlatti G, Serra C, Tovo PA, Vatta S (1999) Polymorphism at codon 54 of mannose-binding protein gene influences AIDS progression but not HIV infection in exposed children. Aids 13:863-4

An P, Bleiber G, Duggal P, Nelson G, May M, Mangeat B, Alobwede I, Trono D, Vlahov D, Donfield S, Goedert JJ, Phair J, Buchbinder S, O'Brien SJ, Telenti A, Winkler CA (2004) APOBEC3G genetic variants and their influence on the progression to AIDS. J Virol 78:11070-6

An P, Martin MP, Nelson GW, Carrington M, Smith MW, Gong K, Vlahov D, O'Brien SJ, Winkler CA (2000) Influence of CCR5 promoter haplotypes on AIDS progression in African-Americans. Aids 14:2117-22

An P, Nelson GW, Wang L, Donfield S, Goedert JJ, Phair J, Vlahov D, Buchbinder S, Farrar WL, Modi W, O'Brien SJ, Winkler CA (2002) Modulating influence on HIV/AIDS by interacting RANTES gene variants. Proc Natl Acad Sci U S A 99:10002-7

An P, Vlahov D, Margolick JB, Phair J, O'Brien TR, Lautenberger J, O'Brien SJ, Winkler CA (2003) A tumor necrosis factor-alpha-inducible promoter variant of interferon-gamma accelerates CD4+ T cell depletion in human immunodeficiency virus-1-infected individuals. J Infect Dis 188:228-31

Anzala AO, Ball TB, Rostron T, O'Brien SJ, Plummer FA, Rowland-Jones SL (1998) CCR2-64I allele and genotype association with delayed AIDS progression in African women. University of Nairobi Collaboration for HIV Research. Lancet 351:1632-3

Balfe P, Churcher Y, Penny M, Easterbrook PJ, Goodall RL, Galpin S, Gotch F, Daniels RS, McKeating JA (1998) Association between a defective CCR-5 gene and progression to disease in HIV infection. AIDS Res Hum Retroviruses 14:1229-34

Balotta C, Bagnarelli P, Violin M, Ridolfo AL, Zhou D, Berlusconi A, Corvasce S, Corbellino M, Clementi M, Clerici M, Moroni M, Galli M (1997) Homozygous delta 32 deletion of the CCR-5 chemokine receptor gene in an HIV-1-infected patient. Aids 11:F67-71

Bird TG, Kaul R, Rostron T, Kimani J, Embree J, Dunn PP, Bwayo JJ, Plummer FA, Rowland-Jones SL, Dong T (2002) HLA typing in a Kenyan cohort identifies novel class I alleles that restrict cytotoxic T-cell responses to local HIV-1 clades. Aids 16:1899-904

Bleiber G, May M, Suarez C, Martinez R, Marzolini C, Egger M, Telenti A (2004) MDR1 genetic polymorphism does not modify either cell permissiveness to HIV-1 or disease progression before treatment. J Infect Dis 189:583-6

Boniotto M, Crovella S, Pirulli D, Scarlatti G, Spano A, Vatta L, Zezlina S, Tovo PA, Palomba E, Amoroso A (2000) Polymorphisms in the MBL2 promoter correlated with risk of HIV-1 vertical transmission and AIDS progression. Genes Immun 1:346-8

Braida L, Boniotto M, Pontillo A, Tovo PA, Amoroso A, Crovella S (2004) A single-nucleotide polymorphism in the human beta-defensin 1 gene is associated with HIV-1 infection in Italian children. Aids 18:1598-600

Bratt G, Leandersson AC, Albert J, Sandstrom E, Wahren B (1998) MT-2 tropism and CCR-5 genotype strongly influence disease progression in HIV-1-infected individuals. Aids 12:729-36

Bream JH, Carrington M, O'Toole S, Dean M, Gerrard B, Shin HD, Kosack D, Modi W, Young HA, Smith MW (2000) Polymorphisms of the human IFNG gene noncoding regions. Immunogenetics 51:50-8

Brinkman BM, Keet IP, Miedema F, Verweij CL, Klein MR (1997) Polymorphisms within the human tumor necrosis factor-alpha promoter region in human immunodeficiency virus type 1-seropositive persons. J Infect Dis 175:188-90

Brockmeyer NH, Potthoff A, Kasper A, Nabring C, Jockel KH, Siffert W (2005) GNB3 C825T polymorphism and response to anti-retroviral combination therapy in HIV-1-infected patients--a pilot study. Eur J Med Res 10:489-94

Brouwer KC, Lal RB, Mirel LB, Yang C, van Eijk AM, Ayisi J, Otieno J, Nahlen BL, Steketee R, Lal AA, Shi YP (2004) Polymorphism of Fc receptor IIa for IgG in infants is associated with susceptibility to perinatal HIV-1 infection. Aids 18:1187-94

Brouwer KC, Yang C, Parekh S, Mirel LB, Shi YP, Otieno J, Lal AA, Lal RB (2005) Effect of CCR2 chemokine receptor polymorphism on HIV type 1 mother-to-child transmission and child survival in Western Kenya. AIDS Res Hum Retroviruses 21:358-62

Bugeja MJ, Booth DR, Bennetts BH, Guerin J, Kaldor JM, Stewart GJ (2004) Analysis of the CCL3-L1 gene for association with HIV-1 susceptibility and disease progression. Aids 18:1069-71

Cameron PU, Mallal SA, French MA, Dawkins RL (1990) Major histocompatibility complex genes influence the outcome of HIV infection. Ancestral haplotypes with C4 null alleles explain diverse HLA associations. Hum Immunol 29:282-95

Capoulade-Metay C, Meyer L, Tran T, Persoz A, Bourdais A, Dudoit Y, Delfraissy JF, Debre P, Theodorou I (2005) Influence of the R22H variant of macrophage inflammatory protein 1beta/Lag-1 in HIV-1 survival. Aids 19:831-3

Carrington M, Nelson GW, Martin MP, Kissner T, Vlahov D, Goedert JJ, Kaslow R, Buchbinder S, Hoots K, O'Brien SJ (1999) HLA and HIV-1: heterozygote advantage and B*35-Cw*04 disadvantage. Science 283:1748-52

Chen Y, Winchester R, Korber B, Gagliano J, Bryson Y, Hutto C, Martin N, McSherry G, Petru A, Wara D, Ammann A (1997) Influence of HLA alleles on the rate of progression of vertically transmitted HIV infection in children: association of several

HLA-DR13 alleles with long-term survivorship and the potential association of HLA-A*2301 with rapid progression to AIDS. Long-Term Survivor Study. Hum Immunol 55:154-62
Cohen JH, Geffriaud C, Caudwell V, Kazatchkine MD (1989) Genetic analysis of CR1 (the C3b complement receptor, CD35) expression on erythrocytes of HIV-infected individuals. Aids 3:397-9
Colobran R, Adreani P, Ashhab Y, Llano A, Este JA, Dominguez O, Pujol-Borrell R, Juan M (2005) Multiple products derived from two CCL4 loci: high incidence of a new polymorphism in HIV+ patients. J Immunol 174:5655-64
Costello C, Tang J, Rivers C, Karita E, Meizen-Derr J, Allen S, Kaslow RA (1999) HLA-B*5703 independently associated with slower HIV-1 disease progression in Rwandan women. Aids 13:1990-1
de Roda Husman AM, Koot M, Cornelissen M, Keet IP, Brouwer M, Broersen SM, Bakker M, Roos MT, Prins M, de Wolf F, Coutinho RA, Miedema F, Goudsmit J, Schuitemaker H (1997) Association between CCR5 genotype and the clinical course of HIV-1 infection. Ann Intern Med 127:882-90
Dean M, Carrington M, Winkler C, Huttley GA, Smith MW, Allikmets R, Goedert JJ, Buchbinder SP, Vittinghoff E, Gomperts E, Donfield S, Vlahov D, Kaslow R, Saah A, Rinaldo C, Detels R, O'Brien SJ (1996) Genetic restriction of HIV-1 infection and progression to AIDS by a deletion allele of the CKR5 structural gene. Hemophilia Growth and Development Study, Multicenter AIDS Cohort Study, Multicenter Hemophilia Cohort Study, San Francisco City Cohort, ALIVE Study. Science 273:1856-62
Delanghe JR, Langlois MR, Boelaert JR, Van Acker J, Van Wanzeele F, van der Groen G, Hemmer R, Verhofstede C, De Buyzere M, De Bacquer D, Arendt V, Plum J (1998) Haptoglobin polymorphism, iron metabolism and mortality in HIV infection. Aids 12:1027-32
Delgado JC, Leung JY, Baena A, Clavijo OP, Vittinghoff E, Buchbinder S, Wolinsky S, Addo M, Walker BD, Yunis EJ, Goldfeld AE (2003) The -1030/-862-linked TNF promoter single-nucleotide polymorphisms are associated with the inability to control HIV-1 viremia. Immunogenetics 55:497-501
Diouf K, Sarr AD, Eisen G, Popper S, Mboup S, Kanki P (2002) Associations between MHC class I and susceptibility to HIV-2 disease progression. J Hum Virol 5:1-7
Do H, Vasilescu A, Diop G, Hirtzig T, Heath SC, Coulonges C, Rappaport J, Therwath A, Lathrop M, Matsuda F, Zagury JF (2005) Exhaustive genotyping of the CEM15 (APOBEC3G) gene and absence of association with AIDS progression in a French cohort. J Infect Dis 191:159-63
Donald JA, Rudman K, Cooper DW, Baumgart KW, Garsia RJ, Gatenby PA, Rickard KA (1992) Progression of HIV-related disease is associated with HLA DQ and DR alleles defined by restriction fragment length polymorphisms. Tissue Antigens 39:241-8
Donninger H, Cashmore TJ, Scriba T, Petersen DC, Janse van Rensburg E, Hayes VM (2004) Functional analysis of novel SLC11A1 (NRAMP1) promoter variants in susceptibility to HIV-1. J Med Genet 41:e49
Dorak MT, Tang J, Penman-Aguilar A, Westfall AO, Zulu I, Lobashevsky ES, Kancheya NG, Schaen MM, Allen SA, Kaslow RA (2004) Transmission of HIV-1 and HLA-B allele-sharing within serodiscordant heterosexual Zambian couples. Lancet 363:2137-9

Easterbrook PJ, Rostron T, Ives N, Troop M, Gazzard BG, Rowland-Jones SL (1999) Chemokine receptor polymorphisms and human immunodeficiency virus disease progression. J Infect Dis 180:1096-105

Eskild A, Jonassen TO, Heger B, Samuelsen SO, Grinde B (1998) The estimated impact of the CCR-5 delta32 gene deletion on HIV disease progression varies with study design. Oslo HIV Cohort Study Group. Aids 12:2271-4

Eugen-Olsen J, Iversen AK, Benfield TL, Koppelhus U, Garred P (1998) Chemokine receptor CCR2b 64I polymorphism and its relation to CD4 T-cell counts and disease progression in a Danish cohort of HIV-infected individuals. Copenhagen AIDS cohort. J Acquir Immune Defic Syndr Hum Retrovirol 18:110-6

Eugen-Olsen J, Iversen AK, Garred P, Koppelhus U, Pedersen C, Benfield TL, Sorensen AM, Katzenstein T, Dickmeiss E, Gerstoft J, Skinhoj P, Svejgaard A, Nielsen JO, Hofmann B (1997) Heterozygosity for a deletion in the CKR-5 gene leads to prolonged AIDS-free survival and slower CD4 T-cell decline in a cohort of HIV-seropositive individuals. Aids 11:305-10

Faure S, Meyer L, Costagliola D, Vaneensberghe C, Genin E, Autran B, Delfraissy JF, McDermott DH, Murphy PM, Debre P, Theodorou I, Combadiere C (2000) Rapid progression to AIDS in HIV+ individuals with a structural variant of the chemokine receptor CX3CR1. Science 287:2274-7

Flores-Villanueva PO, Yunis EJ, Delgado JC, Vittinghoff E, Buchbinder S, Leung JY, Uglialoro AM, Clavijo OP, Rosenberg ES, Kalams SA, Braun JD, Boswell SL, Walker BD, Goldfeld AE (2001) Control of HIV-1 viremia and protection from AIDS are associated with HLA-Bw4 homozygosity. Proc Natl Acad Sci U S A 98:5140-5

Gao X, Nelson GW, Karacki P, Martin MP, Phair J, Kaslow R, Goedert JJ, Buchbinder S, Hoots K, Vlahov D, O'Brien SJ, Carrington M (2001) Effect of a single amino acid change in MHC class I molecules on the rate of progression to AIDS. N Engl J Med 344:1668-75

Gaudieri S, Nolan D, McKinnon E, Witt CS, Mallal S, Christiansen FT (2005) Associations between KIR epitope combinations expressed by HLA-B/-C haplotypes found in an HIV-1 infected study population may influence NK mediated immune responses. Mol Immunol 42:557-60

Geczy AF, Kuipers H, Coolen M, Ashton LJ, Kennedy C, Ng G, Dodd R, Wallace R, Le T, Raynes-Greenow CH, Dyer WB, Learmont JC, Sullivan JS (2000) HLA and other host factors in transfusion-acquired HIV-1 infection. Hum Immunol 61:172-6

Gonzalez E, Dhanda R, Bamshad M, Mummidi S, Geevarghese R, Catano G, Anderson SA, Walter EA, Stephan KT, Hammer MF, Mangano A, Sen L, Clark RA, Ahuja SS, Dolan MJ, Ahuja SK (2001) Global survey of genetic variation in CCR5, RANTES, and MIP-1alpha: impact on the epidemiology of the HIV-1 pandemic. Proc Natl Acad Sci U S A 98:5199-204

Gonzalez E, Kulkarni H, Bolivar H, Mangano A, Sanchez R, Catano G, Nibbs RJ, Freedman BI, Quinones MP, Bamshad MJ, Murthy KK, Rovin BH, Bradley W, Clark RA, Anderson SA, O'Connell R J, Agan BK, Ahuja SS, Bologna R, Sen L, Dolan MJ, Ahuja SK (2005) The influence of CCL3L1 gene-containing segmental duplications on HIV-1/AIDS susceptibility. Science 307:1434-40

Gonzalez E, Rovin BH, Sen L, Cooke G, Dhanda R, Mummidi S, Kulkarni H, Bamshad MJ, Telles V, Anderson SA, Walter EA, Stephan KT, Deucher M, Mangano A, Bologna R, Ahuja SS, Dolan MJ, Ahuja SK (2002) HIV-1 infection and AIDS dementia

are influenced by a mutant MCP-1 allele linked to increased monocyte infiltration of tissues and MCP-1 levels. Proc Natl Acad Sci U S A 99:13795-800

Greggio NA, Cameran M, Giaquinto C, Zacchello F, Koroliuk D, Colizzi V (1993) DNA HLA-DRB1 analysis in children of positive mothers and estimated risk of vertical HIV transmission. Dis Markers 11:29-35

Haas DW, Wu H, Li H, Bosch RJ, Lederman MM, Kuritzkes D, Landay A, Connick E, Benson C, Wilkinson GR, Kessler H, Kim RB (2003) MDR1 gene polymorphisms and phase 1 viral decay during HIV-1 infection: an adult AIDS Clinical Trials Group study. J Acquir Immune Defic Syndr 34:295-8

Hayes VM, Gardiner-Garden M (2003) Are polymorphic markers within the alpha-1-antitrypsin gene associated with risk of human immunodeficiency virus disease? J Infect Dis 188:1205-8

Hayes VM, Petersen DC, Scriba TJ, Zeier M, Grimwood A, Janse van Rensburg E (2002) African-based CCR5 single-nucleotide polymorphism associated with HIV-1 disease progression. Aids 16:2229-31

Hendel H, Caillat-Zucman S, Lebuanec H, Carrington M, O'Brien S, Andrieu JM, Schachter F, Zagury D, Rappaport J, Winkler C, Nelson GW, Zagury JF (1999) New class I and II HLA alleles strongly associated with opposite patterns of progression to AIDS. J Immunol 162:6942-6

Hendel H, Winkler C, An P, Roemer-Binns E, Nelson G, Haumont P, O'Brien S, Khalilli K, Zagury D, Rappaport J, Zagury JF (2001) Validation of genetic case-control studies in AIDS and application to the CX3CR1 polymorphism. J Acquir Immune Defic Syndr 26:507-11

Hersberger M, Bonhoeffer S, Rampini SK, Opravil M, Marti-Jaun J, Telenti A, Hanseler E, Ledergerber B, Speck RF (2004) CCTTT-repeat polymorphism of the inducible nitric oxide synthase is not associated with HIV pathogenesis. Clin Exp Immunol 137:566-9

Hladik F, Liu H, Speelmon E, Livingston-Rosanoff D, Wilson S, Sakchalathorn P, Hwangbo Y, Greene B, Zhu T, McElrath MJ (2005) Combined effect of CCR5-Delta32 heterozygosity and the CCR5 promoter polymorphism -2459 A/G on CCR5 expression and resistance to human immunodeficiency virus type 1 transmission. J Virol 79:11677-84

Hoffman TL, MacGregor RR, Burger H, Mick R, Doms RW, Collman RG (1997) CCR5 genotypes in sexually active couples discordant for human immunodeficiency virus type 1 infection status. J Infect Dis 176:1093-6

Ifergan I, Bernard NF, Bruneau J, Alary M, Tsoukas CM, Roger M (2002) Allele frequency of three functionally active polymorphisms of the MDR-1 gene in high-risk HIV-negative and HIV-positive Caucasians. Aids 16:2340-2

John GC, Bird T, Overbaugh J, Nduati R, Mbori-Ngacha D, Rostron T, Dong T, Kostrikis L, Richardson B, Rowland-Jones SL (2001) CCR5 promoter polymorphisms in a Kenyan perinatal human immunodeficiency virus type 1 cohort: association with increased 2-year maternal mortality. J Infect Dis 184:89-92

John GC, Rousseau C, Dong T, Rowland-Jones S, Nduati R, Mbori-Ngacha D, Rostron T, Kreiss JK, Richardson BA, Overbaugh J (2000) Maternal SDF1 3'A polymorphism is associated with increased perinatal human immunodeficiency virus type 1 transmission. J Virol 74:5736-9

Kageyama S, Mimaya J, Yamada K, Kurimura T, Shiraki K (2001) Polymorphism of CCR5 affecting HIV disease progression in the Japanese population. AIDS Res Hum Retroviruses 17:991-5

Katzenstein TL, Eugen-Olsen J, Hofmann B, Benfield T, Pedersen C, Iversen AK, Sorensen AM, Garred P, Koppelhus U, Svejgaard A, Gerstoft J (1997) HIV-infected individuals with the CCR delta32/CCR5 genotype have lower HIV RNA levels and higher CD4 cell counts in the early years of the infection than do patients with the wild type. Copenhagen AIDS Cohort Study Group. J Acquir Immune Defic Syndr Hum Retrovirol 16:10-4

Keet IP, Tang J, Klein MR, LeBlanc S, Enger C, Rivers C, Apple RJ, Mann D, Goedert JJ, Miedema F, Kaslow RA (1999) Consistent associations of HLA class I and II and transporter gene products with progression of human immunodeficiency virus type 1 infection in homosexual men. J Infect Dis 180:299-309

Khoo SH, Pepper L, Snowden N, Hajeer AH, Vallely P, Wilkins EG, Mandal BK, Ollier WE (1997) Tumour necrosis factor c2 microsatellite allele is associated with the rate of HIV disease progression. Aids 11:423-8

Knuchel MC, Spira TJ, Neumann AU, Xiao L, Rudolph DL, Phair J, Wolinsky SM, Koup RA, Cohen OJ, Folks TM, Lal RB (1998) Analysis of a biallelic polymorphism in the tumor necrosis factor alpha promoter and HIV type 1 disease progression. AIDS Res Hum Retroviruses 14:305-9

Knudsen TB, Kristiansen TB, Katzenstein TL, Eugen-Olsen J (2001) Adverse effect of the CCR5 promoter -2459A allele on HIV-1 disease progression. J Med Virol 65:441-4

Kobayashi N, Nakamura HT, Goto M, Nakamura T, Nakamura K, Sugiura W, Iwamoto A, Kitamura Y (2002) Polymorphisms and haplotypes of the CD209L gene and their association with the clinical courses of HIV-positive Japanese patients. Jpn J Infect Dis 55:131-3

Kroner BL, Goedert JJ, Blattner WA, Wilson SE, Carrington MN, Mann DL (1995) Concordance of human leukocyte antigen haplotype-sharing, CD4 decline and AIDS in hemophilic siblings. Multicenter Hemophilia Cohort and Hemophilia Growth and Development Studies. Aids 9:275-80

Kwa D, van Rij RP, Boeser-Nunnink B, Vingerhoed J, Schuitemaker H (2003) Association between an interleukin-4 promoter polymorphism and the acquisition of CXCR4 using HIV-1 variants. Aids 17:981-5

Li C, Yan YP, Shieh B, Lee CM, Lin RY, Chen YM (1997) Frequency of the CCR5 delta 32 mutant allele in HIV-1-positive patients, female sex workers, and a normal population in Taiwan. J Formos Med Assoc 96:979-84

Li M, Song R, Masciotra S, Soriano V, Spira TJ, Lal RB, Yang C (2005) Association of CCR5 human haplogroup E with rapid HIV type 1 disease progression. AIDS Res Hum Retroviruses 21:111-5

Lichterfeld M, Nischalke HD, van Lunzen J, Sohne J, Schmeisser N, Woitas R, Sauerbruch T, Rockstroh JK, Spengler U (2003) The tandem-repeat polymorphism of the DC-SIGNR gene does not affect the susceptibility to HIV infection and the progression to AIDS. Clin Immunol 107:55-9

Liu C, Carrington M, Kaslow RA, Gao X, Rinaldo CR, Jacobson LP, Margolick JB, Phair J, O'Brien SJ, Detels R (2003) Association of polymorphisms in human leukocyte antigen class I and transporter associated with antigen processing genes with resistance to human immunodeficiency virus type 1 infection. J Infect Dis 187:1404-10

Liu H, Chao D, Nakayama EE, Taguchi H, Goto M, Xin X, Takamatsu JK, Saito H, Ishikawa Y, Akaza T, Juji T, Takebe Y, Ohishi T, Fukutake K, Maruyama Y, Yashiki S, Sonoda S, Nakamura T, Nagai Y, Iwamoto A, Shioda T (1999) Polymorphism in RANTES chemokine promoter affects HIV-1 disease progression. Proc Natl Acad Sci U S A 96:4581-5

Liu H, Hwangbo Y, Holte S, Lee J, Wang C, Kaupp N, Zhu H, Celum C, Corey L, McElrath MJ, Zhu T (2004) Analysis of genetic polymorphisms in CCR5, CCR2, stromal cell-derived factor-1, RANTES, and dendritic cell-specific intercellular adhesion molecule-3-grabbing nonintegrin in seronegative individuals repeatedly exposed to HIV-1. J Infect Dis 190:1055-8

Liu M, Wang F, Hong W, Wang B, Jin L, Lei Z, Hou J (2002) [Identification of new mutant sites and 894C deletion variant genotyping of HIV-1 coreceptor CCR5 in indigenous Chinese populations]. Zhonghua Yi Xue Za Zhi 82:1468-72

Lockett SF, Alonso A, Wyld R, Martin MP, Robertson JR, Gore SM, Leen CL, Brettle RP, Yirrell DL, Carrington M, Brown AJ (1999) Effect of chemokine receptor mutations on heterosexual human immunodeficiency virus transmission. J Infect Dis 180:614-21

Lugli E, Pinti M, Nasi M, Troiano L, Prada N, Mussini C, Borghi V, Esposito R, Cossarizza A (2005) MMP-7 promoter polymorphisms do not influence CD4+ recovery and changes in plasma viral load during antiretroviral therapy for HIV-1 infection. Int J Immunogenet 32:269-71

Ma L, Marmor M, Zhong P, Ewane L, Su B, Nyambi P (2005) Distribution of CCR2-64I and SDF1-3'A alleles and HIV status in 7 ethnic populations of Cameroon. J Acquir Immune Defic Syndr 40:89-95

Maas J, de Roda Husman AM, Brouwer M, Krol A, Coutinho R, Keet I, van Leeuwen R, Schuitemaker H (1998) Presence of the variant mannose-binding lectin alleles associated with slower progression to AIDS. Amsterdam Cohort Study. Aids 12:2275-80

MacDonald KS, Fowke KR, Kimani J, Dunand VA, Nagelkerke NJ, Ball TB, Oyugi J, Njagi E, Gaur LK, Brunham RC, Wade J, Luscher MA, Krausa P, Rowland-Jones S, Ngugi E, Bwayo JJ, Plummer FA (2000) Influence of HLA supertypes on susceptibility and resistance to human immunodeficiency virus type 1 infection. J Infect Dis 181:1581-9

Malik S, Arias M, Di Flumeri C, Garcia LF, Schurr E (2003) Absence of association between mannose-binding lectin gene polymorphisms and HIV-1 infection in a Colombian population. Immunogenetics 55:49-52

Mangano A, Gonzalez E, Dhanda R, Catano G, Bamshad M, Bock A, Duggirala R, Williams K, Mummidi S, Clark RA, Ahuja SS, Dolan MJ, Bologna R, Sen L, Ahuja SK (2001) Concordance between the CC chemokine receptor 5 genetic determinants that alter risks of transmission and disease progression in children exposed perinatally to human immunodeficiency virus. J Infect Dis 183:1574-85

Mangano A, Kopka J, Batalla M, Bologna R, Sen L (2000) Protective effect of CCR2-64I and not of CCR5-delta32 and SDF1-3'A in pediatric HIV-1 infection. J Acquir Immune Defic Syndr 23:52-7

Marquet S, Sanchez FO, Arias M, Rodriguez J, Paris SC, Skamene E, Schurr E, Garcia LF (1999) Variants of the human NRAMP1 gene and altered human immunodeficiency virus infection susceptibility. J Infect Dis 180:1521-5

Martin MP, Dean M, Smith MW, Winkler C, Gerrard B, Michael NL, Lee B, Doms RW, Margolick J, Buchbinder S, Goedert JJ, O'Brien TR, Hilgartner MW, Vlahov D, O'Brien SJ, Carrington M (1998) Genetic acceleration of AIDS progression by a promoter variant of CCR5. Science 282:1907-11

Martin MP, Gao X, Lee JH, Nelson GW, Detels R, Goedert JJ, Buchbinder S, Hoots K, Vlahov D, Trowsdale J, Wilson M, O'Brien SJ, Carrington M (2002) Epistatic interaction between KIR3DS1 and HLA-B delays the progression to AIDS. Nat Genet 31:429-34

Martin MP, Lederman MM, Hutcheson HB, Goedert JJ, Nelson GW, van Kooyk Y, Detels R, Buchbinder S, Hoots K, Vlahov D, O'Brien SJ, Carrington M (2004) Association of DC-SIGN promoter polymorphism with increased risk for parenteral, but not mucosal, acquisition of human immunodeficiency virus type 1 infection. J Virol 78:14053-6

Mazzucchelli R, Corvasce S, Violin M, Riva C, Bianchi R, Deho L, Velleca R, Cibella J, Bada M, Moroni M, Galli M, Balotta C (2001) Role of CCR5, CCR2 and SDF-1 gene polymorphisms in a population of HIV-1 infected individuals. J Biol Regul Homeost Agents 15:265-71

McDermott DH, Beecroft MJ, Kleeberger CA, Al-Sharif FM, Ollier WE, Zimmerman PA, Boatin BA, Leitman SF, Detels R, Hajeer AH, Murphy PM (2000a) Chemokine RANTES promoter polymorphism affects risk of both HIV infection and disease progression in the Multicenter AIDS Cohort Study. Aids 14:2671-8

McDermott DH, Colla JS, Kleeberger CA, Plankey M, Rosenberg PS, Smith ED, Zimmerman PA, Combadiere C, Leitman SF, Kaslow RA, Goedert JJ, Berger EA, O'Brien TR, Murphy PM (2000b) Genetic polymorphism in CX3CR1 and risk of HIV disease. Science 290:2031

McDermott DH, Zimmerman PA, Guignard F, Kleeberger CA, Leitman SF, Murphy PM (1998) CCR5 promoter polymorphism and HIV-1 disease progression. Multicenter AIDS Cohort Study (MACS). Lancet 352:866-70

McIntyre MQ, Price P, Franchina M, French MA, Abraham LJ (2003) Distribution of human CD30 gene promoter microsatellite alleles in healthy and human immunodeficiency virus-1 infected populations. Eur J Immunogenet 30:125-8

McNeil AJ, Yap PL, Gore SM, Brettle RP, McColl M, Wyld R, Davidson S, Weightman R, Richardson AM, Robertson JR (1996) Association of HLA types A1-B8-DR3 and B27 with rapid and slow progression of HIV disease. Qjm 89:177-85

Meyer L, Magierowska M, Hubert JB, Rouzioux C, Deveau C, Sanson F, Debre P, Delfraissy JF, Theodorou I (1997) Early protective effect of CCR-5 delta 32 heterozygosity on HIV-1 disease progression: relationship with viral load. The SEROCO Study Group. Aids 11:F73-8

Migueles SA, Sabbaghian MS, Shupert WL, Bettinotti MP, Marincola FM, Martino L, Hallahan CW, Selig SM, Schwartz D, Sullivan J, Connors M (2000) HLA B*5701 is highly associated with restriction of virus replication in a subgroup of HIV-infected long term nonprogressors. Proc Natl Acad Sci U S A 97:2709-14

Modi WS, Goedert JJ, Strathdee S, Buchbinder S, Detels R, Donfield S, O'Brien SJ, Winkler C (2003a) MCP-1-MCP-3-Eotaxin gene cluster influences HIV-1 transmission. Aids 17:2357-65

Modi WS, O'Brien TR, Vlahov D, Buchbinder S, Gomperts E, Phair J, O'Brien SJ, Winkler C (2003b) Haplotype diversity in the interleukin-4 gene is not associated with HIV-1 transmission and AIDS progression. Immunogenetics 55:157-64

Modi WS, Scott K, Goedert JJ, Vlahov D, Buchbinder S, Detels R, Donfield S, O'Brien S J, Winkler C (2005) Haplotype analysis of the SDF-1 (CXCL12) gene in a longitudinal HIV-1/AIDS cohort study. Genes Immun 6:691-8

Morawetz RA, Rizzardi GP, Glauser D, Rutschmann O, Hirschel B, Perrin L, Opravil M, Flepp M, von Overbeck J, Glauser MP, Ghezzi S, Vicenzi E, Poli G, Lazzarin A, Pantaleo G (1997) Genetic polymorphism of CCR5 gene and HIV disease: the heterozygous (CCR5/delta ccr5) genotype is neither essential nor sufficient for protection against disease progression. Swiss HIV Cohort. Eur J Immunol 27:3223-7

Motta P, Marinic K, Sorrentino A, Lopez R, Iliovich E, Habegger de Sorrentino A (2002) Association of HLA-DQ and HLA-DR alleles with susceptibility or resistance to HIV-1 infection among the population of Chaco Province, Argentina. Medicina (B Aires) 62:245-8

Mulherin SA, O'Brien TR, Ioannidis JP, Goedert JJ, Buchbinder SP, Coutinho RA, Jamieson BD, Meyer L, Michael NL, Pantaleo G, Rizzardi GP, Schuitemaker H, Sheppard HW, Theodorou ID, Vlahov D, Rosenberg PS (2003) Effects of CCR5-Delta32 and CCR2-64I alleles on HIV-1 disease progression: the protection varies with duration of infection. Aids 17:377-87

Mummidi S, Ahuja SS, Gonzalez E, Anderson SA, Santiago EN, Stephan KT, Craig FE, O'Connell P, Tryon V, Clark RA, Dolan MJ, Ahuja SK (1998) Genealogy of the CCR5 locus and chemokine system gene variants associated with altered rates of HIV-1 disease progression. Nat Med 4:786-93

Munkanta M, Terunuma H, Takahashi M, Hanabusa H, Miura T, Ikeda S, Sakai M, Fujii T, Takahashi Y, Oka S, Matsuda J, Ishikawa M, Taki M, Takashima Y, Mimaya J, Ito M, Kimura A, Yasunami M (2005) HLA-B polymorphism in Japanese HIV-1-infected long-term surviving hemophiliacs. Viral Immunol 18:500-5

Nakayama EE, Hoshino Y, Xin X, Liu H, Goto M, Watanabe N, Taguchi H, Hitani A, Kawana-Tachikawa A, Fukushima M, Yamada K, Sugiura W, Oka SI, Ajisawa A, Sato H, Takebe Y, Nakamura T, Nagai Y, Iwamoto A, Shioda T (2000a) Polymorphism in the interleukin-4 promoter affects acquisition of human immunodeficiency virus type 1 syncytium-inducing phenotype. J Virol 74:5452-9

Nakayama EE, Meyer L, Iwamoto A, Persoz A, Nagai Y, Rouzioux C, Delfraissy JF, Debre P, McIlroy D, Theodorou I, Shioda T (2002) Protective effect of interleukin-4 -589T polymorphism on human immunodeficiency virus type 1 disease progression: relationship with virus load. J Infect Dis 185:1183-6

Nakayama EE, Wasi C, Ajisawa A, Iwamoto A, Shioda T (2000b) A new polymorphism in the promoter region of the human interleukin-16 (IL-16) gene. Genes Immun 1:293-4

Nguyen L, Li M, Chaowanachan T, Hu DJ, Vanichseni S, Mock PA, van Griensven F, Martin M, Sangkum U, Choopanya K, Tappero JW, Lal RB, Yang C (2004) CCR5 promoter human haplogroups associated with HIV-1 disease progression in Thai injection drug users. Aids 18:1327-33

O'Brien TR, McDermott DH, Ioannidis JP, Carrington M, Murphy PM, Havlir DV, Richman DD (2000) Effect of chemokine receptor gene polymorphisms on the response to potent antiretroviral therapy. Aids 14:821-6

Ometto L, Bertorelle R, Mainardi M, Giurisato M, Chieco-Bianchi L, De Rossi A (1999) Analysis of the CC chemokine receptor 5 m303 mutation in infants born to HIV-1-seropositive mothers. Aids 13:871-2

Ometto L, Bertorelle R, Mainardi M, Zanchetta M, Tognazzo S, Rampon O, Ruga E, Chieco-Bianchi L, De Rossi A (2001) Polymorphisms in the CCR5 promoter region influence disease progression in perinatally human immunodeficiency virus type 1-infected children. J Infect Dis 183:814-8

Passam AM, Zafiropoulos A, Miyakis S, Zagoreos I, Stavrianeas NG, Krambovitis E, Spandidos DA (2005) CCR2-64I and CXCL12 3'A alleles confer a favorable prognosis to AIDS patients undergoing HAART therapy. J Clin Virol 34:302-9

Petersen DC, Laten A, Zeier MD, Grimwood A, Rensburg EJ, Hayes VM (2002) Novel mutations and SNPs identified in CCR2 using a new comprehensive denaturing gradient gel electrophoresis assay. Hum Mutat 20:253-9

Philpott S, Burger H, Charbonneau T, Grimson R, Vermund SH, Visosky A, Nachman S, Kovacs A, Tropper P, Frey H, Weiser B (1999) CCR5 genotype and resistance to vertical transmission of HIV-1. J Acquir Immune Defic Syndr 21:189-93

Philpott S, Burger H, Tarwater PM, Lu M, Gange SJ, Anastos K, Cohen M, Greenblatt RM, Kovacs A, Minkoff H, Young M, Miotti P, Dupuis M, Weiser B (2004) CCR2 genotype and disease progression in a treated population of HIV type 1-infected women. Clin Infect Dis 39:861-5

Price P, James I, Fernandez S, French MA (2004) Alleles of the gene encoding IL-1alpha may predict control of plasma viraemia in HIV-1 patients on highly active antiretroviral therapy. Aids 18:1495-501

Pronk JC, Frants RR, Crusius B, Eriksson AW, de Wolf F, Boucher CA, Bakker M, Goudsmit J (1988) No predictive value of GC phenotypes for HIV infection and progression to AIDS. Hum Genet 80:181-2

Quaye IK, Brandful J, Ekuban FA, Gyan B, Ankrah NA (2000a) Haptoglobin polymorphism in human immunodeficiency virus infection: Hp0 phenotype limits depletion of CD4 cell counts in HIV-1-seropositive individuals. J Infect Dis 181:1483-5

Quaye IK, Ekuban FA, Brandful JA, Gyan BA, Akanmori BD, Ankrah NA (2000b) Haptoglobin phenotypes in HIV-1-seropositive patients in Ghana: decreased risk for Hp0 individuals. Hum Hered 50:382-3

Riabov GS, Kazennova EV, Korepanova LB, Mal'tseva EA, Zhalnin VV, Krasnikova LA, Zverev S, Pokrovskii VV, Bobkov AF, Weber JN (2002) [The HIV-infection outbreak in the town Lys'va (Perm region): homozygote genotype CCR5 delta32/CCR5 delta32 provides the high level of the persistence in the parenteral transmission of the virus]. Vopr Virusol 47:13-6

Rohowsky-Kochan C, Skurnick J, Molinaro D, Louria D (1998) HLA antigens associated with susceptibility/resistance to HIV-1 infection. Hum Immunol 59:802-15

Roman F, Franck N, Burgy C, Servais J, Zimmer JM, Mossong J, Goubau P, Schneider F, Hemmer R, Schmit JC (2002) Prevalence of HIV co-receptor polymorphisms in HIV-infected patients and uninfected volunteers in Luxembourg. HIV Clin Trials 3:195-201

Royo JL, Ruiz A, Borrego S, Rubio A, Sanchez B, Nunez-Roldan A, Lissen E, Antinolo G (2001) Fluorescence resonance energy transfer analysis of CCR-V64I and SDF1-3'a polymorphisms: prevalence in southern Spain hiv type 1+ cohort and noninfected population. AIDS Res Hum Retroviruses 17:663-6

Saitoh A, Singh KK, Powell CA, Fenton T, Fletcher CV, Brundage R, Starr S, Spector SA (2005) An MDR1-3435 variant is associated with higher plasma nelfinavir levels and more rapid virologic response in HIV-1 infected children. Aids 19:371-80

Sei S, Boler AM, Nguyen GT, Stewart SK, Yang QE, Edgerly M, Wood LV, Brouwers P, Venzon DJ (2001) Protective effect of CCR5 delta 32 heterozygosity is restricted by SDF-1 genotype in children with HIV-1 infection. Aids 15:1343-52

Shin HD, Winkler C, Stephens JC, Bream J, Young H, Goedert JJ, O'Brien TR, Vlahov D, Buchbinder S, Giorgi J, Rinaldo C, Donfield S, Willoughby A, O'Brien SJ, Smith MW (2000) Genetic restriction of HIV-1 pathogenesis to AIDS by promoter alleles of IL10. Proc Natl Acad Sci U S A 97:14467-72

Singh KK, Barroga CF, Hughes MD, Chen J, Raskino C, McKinney RE, Spector SA (2003) Genetic influence of CCR5, CCR2, and SDF1 variants on human immunodeficiency virus 1 (HIV-1)-related disease progression and neurological impairment, in children with symptomatic HIV-1 infection. J Infect Dis 188:1461-72

Singh KK, Hughes MD, Chen J, Spector SA (2004) Lack of protective effects of interleukin-4 -589-C/T polymorphism against HIV-1-related disease progression and central nervous system impairment, in children. J Infect Dis 189:587-92

Singh KK, Hughes MD, Chen J, Spector SA (2005) Genetic polymorphisms in CX3CR1 predict HIV-1 disease progression in children independently of CD4+ lymphocyte count and HIV-1 RNA load. J Infect Dis 191:1971-80

Smith MW, Dean M, Carrington M, Winkler C, Huttley GA, Lomb DA, Goedert JJ, O'Brien TR, Jacobson LP, Kaslow R, Buchbinder S, Vittinghoff E, Vlahov D, Hoots K, Hilgartner MW, O'Brien SJ (1997) Contrasting genetic influence of CCR2 and CCR5 variants on HIV-1 infection and disease progression. Hemophilia Growth and Development Study (HGDS), Multicenter AIDS Cohort Study (MACS), Multicenter Hemophilia Cohort Study (MHCS), San Francisco City Cohort (SFCC), ALIVE Study. Science 277:959-65

Soriano A, Martinez C, Garcia F, Plana M, Palou E, Lejeune M, Arostegui JI, De Lazzari E, Rodriguez C, Barrasa A, Lorenzo JI, Alcami J, del Romero J, Miro JM, Gatell JM, Gallart T (2002) Plasma stromal cell-derived factor (SDF)-1 levels, SDF1-3'A genotype, and expression of CXCR4 on T lymphocytes: their impact on resistance to human immunodeficiency virus type 1 infection and its progression. J Infect Dis 186:922-31

Tang J, Penman-Aguilar A, Lobashevsky E, Allen S, Kaslow RA (2004) HLA-DRB1 and -DQB1 alleles and haplotypes in Zambian couples and their associations with heterosexual transmission of HIV type 1. J Infect Dis 189:1696-704

Tang J, Shelton B, Makhatadze NJ, Zhang Y, Schaen M, Louie LG, Goedert JJ, Seaberg EC, Margolick JB, Mellors J, Kaslow RA (2002a) Distribution of chemokine receptor CCR2 and CCR5 genotypes and their relative contribution to human immunodeficiency virus type 1 (HIV-1) seroconversion, early HIV-1 RNA concentration in plasma, and later disease progression. J Virol 76:662-72

Tang J, Wilson CM, Meleth S, Myracle A, Lobashevsky E, Mulligan MJ, Douglas SD, Korber B, Vermund SH, Kaslow RA (2002b) Host genetic profiles predict virological and immunological control of HIV-1 infection in adolescents. Aids 16:2275-84

Tang J, Wilson CM, Schaen M, Myracle A, Douglas SD, Kaslow RA (2002c) CCR2 and CCR5 genotypes in HIV type 1-infected adolescents: limited contributions to variability in plasma HIV type 1 RNA concentration in the absence of antiretroviral therapy. AIDS Res Hum Retroviruses 18:403-12

Tiensiwakul P (2004) Stromal cell-derived factor (SDF) 1-3'A polymorphism may play a role in resistance to HIV-1 infection in seronegative high-risk Thais. Intervirology 47:87-92

Tresoldi E, Romiti ML, Boniotto M, Crovella S, Salvatori F, Palomba E, Pastore A, Cancrini C, de Martino M, Plebani A, Castelli G, Rossi P, Tovo PA, Amoroso A, Scarlatti G (2002) Prognostic value of the stromal cell-derived factor 1 3'A mutation in pediatric human immunodeficiency virus type 1 infection. J Infect Dis 185:696-700

van Rij RP, Broersen S, Goudsmit J, Coutinho RA, Schuitemaker H (1998) The role of a stromal cell-derived factor-1 chemokine gene variant in the clinical course of HIV-1 infection. Aids 12:F85-90

Vasilescu A, Heath SC, Diop G, Do H, Hirtzig T, Hendel H, Bertin-Maghit S, Rappaport J, Therwath A, Lathrop GM, Matsuda F, Zagury JF (2004) Genomic analysis of Fas and FasL genes and absence of correlation with disease progression in AIDS. Immunogenetics 56:56-60

Vasilescu A, Heath SC, Ivanova R, Hendel H, Do H, Mazoyer A, Khadivpour E, Goutalier FX, Khalili K, Rappaport J, Lathrop GM, Matsuda F, Zagury JF (2003) Genomic analysis of Th1-Th2 cytokine genes in an AIDS cohort: identification of IL4 and IL10 haplotypes associated with the disease progression. Genes Immun 4:441-9

Vidal F, Peraire J, Domingo P, Broch M, Cairo M, Pedrol E, Montero M, Vilades C, Gutierrez C, Sambeat MA, Fontanet A, Dalmau D, Deig E, Knobel H, Sirvent JJ, Richart C (2006) Polymorphism of RANTES Chemokine Gene Promoter Is Not Associated With Long-Term Nonprogressive HIV-1 Infection of More Than 16 Years. J Acquir Immune Defic Syndr 41:17-22

Vidal F, Peraire J, Domingo P, Broch M, Knobel H, Pedrol E, Dalmau D, Vilades C, Sambeat MA, Gutierrez C, Richart C (2005a) Lack of association of SDF-1 3'A variant allele with long-term nonprogressive HIV-1 infection is extended beyond 16 years. J Acquir Immune Defic Syndr 40:276-9

Vidal F, Vilades C, Domingo P, Broch M, Pedrol E, Dalmau D, Knobel H, Peraire J, Gutierrez C, Sambeat MA, Fontanet A, Deig E, Cairo M, Montero M, Richart C, Mallal S (2005b) Spanish HIV-1-infected long-term nonprogressors of more than 15 years have an increased frequency of the CX3CR1 249I variant allele. J Acquir Immune Defic Syndr 40:527-31

Vyakarnam A, Sidebottom D, Murad S, Underhill JA, Easterbrook PJ, Dalgleish AG, Peakman M (2004) Possession of human leucocyte antigen DQ6 alleles and the rate of CD4 T-cell decline in human immunodeficiency virus-1 infection. Immunology 112:136-42

Watanabe MA, de Oliveira Cavassin GG, Orellana MD, Milanezi CM, Voltarelli JC, Kashima S, Covas DT (2003) SDF-1 gene polymorphisms and syncytia induction in Brazilian HIV-1 infected individuals. Microb Pathog 35:31-4

Winkler C, Modi W, Smith MW, Nelson GW, Wu X, Carrington M, Dean M, Honjo T, Tashiro K, Yabe D, Buchbinder S, Vittinghoff E, Goedert JJ, O'Brien TR, Jacobson LP, Detels R, Donfield S, Willoughby A, Gomperts E, Vlahov D, Phair J, O'Brien SJ (1998) Genetic restriction of AIDS pathogenesis by an SDF-1 chemokine gene variant. ALIVE Study, Hemophilia Growth and Development Study (HGDS), Multicenter AIDS Cohort Study (MACS), Multicenter Hemophilia Cohort Study (MHCS), San Francisco City Cohort (SFCC). Science 279:389-93

Winkler CA, Hendel H, Carrington M, Smith MW, Nelson GW, O'Brien S J, Phair J, Vlahov D, Jacobson LP, Rappaport J, Vasilescu A, Bertin-Maghit S, An P, Lu W, Andrieu JM, Schachter F, Therwath A, Zagury JF (2004) Dominant effects of CCR2-CCR5 haplotypes in HIV-1 disease progression. J Acquir Immune Defic Syndr 37:1534-8

Witkin SS, Linhares IM, Gerber S, Caetano ME, Segurado AC (2001) Interleukin-1 receptor antagonist gene polymorphism and circulating levels of human immunodeficiency virus type 1 RNA in Brazilian women. J Virol 75:6242-4

Xin X, Nakamura K, Liu H, Nakayama EE, Goto M, Nagai Y, Kitamura Y, Shioda T, Iwamoto A (2001) Novel polymorphisms in human macrophage inflammatory protein-1 alpha (MIP-1alpha) gene. Genes Immun 2:156-8

Yang C, Boone L, Nguyen TX, Rudolph D, Limpakarnjanarat K, Mastro TD, Tappero J, Cole AM, Lal RB (2005) theta-Defensin pseudogenes in HIV-1-exposed, persistently seronegative female sex-workers from Thailand. Infect Genet Evol 5:11-5

Yang C, Li M, Limpakarnjanarat K, Young NL, Hodge T, Butera ST, McNicholl JM, Mastro TD, Lal RB (2003) Polymorphisms in the CCR5 coding and noncoding regions among HIV type 1-exposed, persistently seronegative female sex-workers from Thailand. AIDS Res Hum Retroviruses 19:661-5

Zhao XY, Lee SS, Wong KH, Chan KC, Ma S, Yam WC, Yuen KY, Ng MH, Zheng BJ (2004) Effects of single nucleotide polymorphisms in the RANTES promoter region in healthy and HIV-infected indigenous Chinese. Eur J Immunogenet 31:179-83

Zhao XY, Lee SS, Wong KH, Chan KC, Ng F, Chan CC, Han D, Yam WC, Yuen KY, Ng MH, Zheng BJ (2005) Functional analysis of naturally occurring mutations in the open reading frame of CCR5 in HIV-infected Chinese patients and healthy controls. J Acquir Immune Defic Syndr 38:509-17

Zimmerman PA, Buckler-White A, Alkhatib G, Spalding T, Kubofcik J, Combadiere C, Weissman D, Cohen O, Rubbert A, Lam G, Vaccarezza M, Kennedy PE, Kumaraswami V, Giorgi JV, Detels R, Hunter J, Chopek M, Berger EA, Fauci AS, Nutman TB, Murphy PM (1997) Inherited resistance to HIV-1 conferred by an inactivating mutation in CC chemokine receptor 5: studies in populations with contrasting clinical phenotypes, defined racial background, and quantified risk. Mol Med 3:23-36
