# Supplementary material for: Genetic susceptibility to infectious diseases: big is beautiful, but will bigger be even better?
Source: Lancet Infect Dis. 2006 Oct;6(10):653–63. doi: 10.1016/S1473-3099(06)70601-6 (PMC2330096; doi:10.1016/S1473-3099(06)70601-6)
Supplement: Supplementary file 2 [file mmc2.pdf]

On-line Supplementary Table 2 - Mycobacterial Diseases

**Papers Reporting Significant Linkage or Association**						
**Candidate Gene**	**Population**	**Phenotype**	**Sample Size**	**Reported Results**	**Year**	**Reference**
**MHC Class I Region:**						
Aw24	Japanese?	Leprosy per se	Ca = 59; Co = 125	25.4% (Ca) vs 63.2% (Co)	1977	[Nakajima, 1977 #188]
Bw15	African American	PTB with Cavitation	Ca = 60; Co = 100	Increased Frequency	1979	[Al-Arif, 1979 #178]
B7; Bw54	Japanese	LL Type	Ca = 295; Co = 110	N/A	1982	[Izumi; 1982 #96]
HLA -A; -B	Chinese (Jiangsu)	LL vs TT Leprosy	26 Families	Increased Transmission (p < 0.05)	1985	[Xu, 1985 #186]
A2; B5	Egyptian	PTB	Ca = 42; Co = 156	N/A	1985	[Hafez; 1985 #12]
A11; Aw33	Korean	Leprosy per se	Ca = 157; Co = 162	Increased Frequency	1987	[Kim, 1987 #183]
Bw46	Thai	PTB	Ca = 35; Co = 35?	Increased Frequency	1988	[Chandanayingyong, 1988 #171]
B12	Thai	PTB	Ca = 35; Co = 35?	Decreased Frequency	1988	[Chandanayingyong, 1988 #171]
A10; B8	Indian (South)	Smear -ve PTB	Ca = 152; Co = 404	p < 0.01	1991	[Brahmajothi; 1991 #14]
Cw3	Japanese	Episcleritis in Leprosy	Ca = 79 (33/46); Co = 114	OR = 2.6; p < 0.05	1998	[Joko, 1998 #181]
B46/MICA-5A5	Chinese	LL Type	Ca = 69; Co = 112	RR = 0.22; p < 0.01	1999	[Wang; 1999 #53]
A3; 9; 10; 32	Turkish	Leprosy per se	Ca = 80; Co = 120	p < 0.02	2002	[Kocak; 2002 #54]
B5; 21; 44; 49	Turkish	Leprosy per se	Ca = 80; Co = 120	p < 0.03	2002	[Kocak; 2002 #54]
Bw4; 6; Cw1; 2	Turkish	Leprosy per se	Ca = 80; Co = 120	p < 0.005	2002	[Kocak; 2002 #54]
A; B & Cw	Indian	Leprosy per se	Ca = 32; Co = 67	N/A	2003	[Shankarkumar; 2003 #55]
A26; B17; B27; DR14	Iranian	PTB	Ca = 44; Co = 108	p < 0.05 (pc > 0.05)	2003	[Mahmoudzadeh-Niknam; 2003 #91]
A2, A11, B40, Cw7	Mumbai	Leprosy per se	Ca = 103; Co = 101	OR = 13.33 - 2.21; p < 0.009	2004	[Shankarkumar, 2004 #179]
A28, B12, B15, Cw3	Mumbai	Leprosy per se	Ca = 103; Co = 101	OR = 0.25 - 0.07; p < 0.05	2004	[Shankarkumar, 2004 #179]
A1 Supertype	Indian	PTB & Miliary TB	Ca = 235; Co = 289	OR = 0.43; pc = 0.001	2004	[Balamurugan; 2004 #103]
Cw Allotype 1	Indian	PTB & Miliary TB	Ca = 235; Co = 289	OR = 1.69; p = 0.005	2004	[Balamurugan; 2004 #103]
Cw Allotype 2	Indian	PTB & Miliary TB	Ca = 235; Co = 289	OR = 2.31; p = 0.000004	2004	[Balamurugan; 2004 #103]
B14	Italian	Cavitary TB	Ca = 54; Co = 1089	RR = 3.9; p = 0.001	2004	[Ruggiero; 2004 #110]
**MHC Class II Region:**						
DR2	Indian?	TT vs LL Leprosy	? Families	p = 0.002	1980	[van Eden, 1980 #185]
DR2	Japanese	LL Type	Ca = 84; Co = 55	RR = 8.7	1982	[Izumi; 1982 #96]
DR2	Japanese	TT Type	Ca = 28; Co = 55	RR = 5.9	1982	[Izumi; 1982 #96]
DR3	Suriname	TT Type	Ca = 73; Co = 92	p = 0.0003	1982	[van Eden; 1982 #56]
DR2	Indian	PTB	25 Families	p = 0.001	1983	[Singh, 1983 #176]
DR2	Thai	TT Type	Ca = 32; Co = 32	RR = 7.4; p = 0.02	1985	[Schauf; 1985 #57]
DQw1	Thai	TT Type	Ca = 32; Co = 32	N/A	1985	[Schauf; 1985 #57]
DR3	?	TT vs LL Leprosy	28 Families	p = 0.02	1985	[van Eden, 1985 #184]
DR1; DR2; DRw9; DQw1	Korean	Leprosy per se	Ca = 157; Co = 162	Increased Frequency	1987	[Kim, 1987 #183]
DR4; DRw53; DQw3	Korean	Leprosy per se	Ca = 157; Co = 162	Decreased Frequency	1987	[Kim, 1987 #183]
DQw1c	Melanesian	Leprosy per se	N/A	N/A	1988	[Jazwinska, 1988 #160]
DR3	Mexican	PTB	Ca = 51; Co = 54	Decreased Frequency	1988	[Cox, 1988 #170]
DR4	Thai	PTB	Ca = 35; Co = 35?	Increased Frequency	1988	[Chandanayingyong, 1988 #171]

On-line Supplementary Table 2 - Mycobacterial Diseases

DR2; DQw1	Indonesian	PTB	Ca = 101; Co = 65	Attributable risk = 36% & 39%	1989	[Bothamley; 1989 #13]
DR2 & DQw1	Caucasian	Leprosy per se	Ca = 351+; Co = 274+	RR = 2.65 & 2.73	1990	[Todd; 1990 #58]
DR2	Russian	PTB	N/A	Increased Frequency	1990	[Khomenko, 1990 #168]
DR3	Russian	PTB	N/A	Decreased Frequency	1990	[Khomenko, 1990 #168]
DR2	Indian (South)	Smear +ve PTB	Ca = 204; Co = 404	Attributable risk = 0.29; p = 0.01	1991	[Brahmajothi; 1991 #14]
DRB1*1501	North Indian	LL Type	Ca = 93; Co = 47	RR = 16.3	1993	[Rani; 1993 #59]
DRB1*1502	Asian Indian	TT Type	N/A	p < 0.05	1995	[Mehra; 1995 #60]
DRB1*1501 & *1502	Indian	TT Type	Ca = 54; Co = 44	p = 5x10-6	1996	[Zerva; 1996 #61]
DR2; DRw53	Tuvinian Russian	PTB	N/A	N/A	1996	[Pospelov, 1996 #165]
DR2	Indian (North)	PTB	Ca = 153; Co = 289	RR = 1.8; pc = 0.029	1996	[Rajalingam; 1996 #15]
TAP2B	North Indian	TT Type	Ca = 50; Co = 40	RR = 3.4; p < 0.03	1997	[Rajalingam; 1997 #68]
DRB1*02	Indonesian	LL Type	Ca = 79; Co = 50	OR = 2.54; p = 0.037	1997	[Soebono; 1997 #62]
TAP-A/F	Indian (North)	PTB	Ca = 57; Co – 40	RR = 4.3; pc = 0.01	1997	[Rajalingam; 1997 #68]
DR4; DRB1*0405; DQB1*0401	Japanese	Episcleritis in Leprosy	Ca = 79 (33/46); Co = 114	OR = 0.21 - 0.04; p < 0.001	1998	[Joko, 1998 #181]
DQB1*0503	Cambodian	PTB	Ca = 126; Co = 88	p = 0.005	1998	[Goldfeld; 1998 #16]
DR2; DQ1	Indian	PTB	Ca = 209; Co = 122	RR = 2.3; RR = 2.8	1998	[Selvaraj, 1998 #164]
DR4; DQB1*0302	Japanese	Uveitis in Leprosy	Ca = 93 (46/47); Co = 114	OR = 0.28 - 0.21; p < 0.05	1999	[Joko, 1999 #180]
DR2; DRB*1501; DRB1*0405; DQB1*0302	Japanese	Uveitis in Leprosy	Ca = 93 (46/47); Co = 114	OR = 9.5 - 7.2; p < 0.00005	1999	[Joko, 1999 #180]
DRB1 alleles	Nigerian	Leprosy per se	Ca = 287; Co = 170	OR > 2.4; p < 0.05	1999	[Uko; 1999 #63]
DRB1*1501; DQB1*0601	Southern Indian	Sputum +ve PTB	Ca = 126; Co = 87	OR = 2.68; OR = 2.32	1999	[Ravikumar; 1999 #18]
DQA1*0101; DQB1*0501; DRB1*1501	Mexican	PTB	Ca = 65; Co = 95	OR = 6.16 – 7.92	1999	[Teran-Escandon; 1999 #17]
DQB1*0402; DR4; DR8	Mexican	PTB	Ca = 65; Co = 95	Decreased Frequency	1999	[Teran-Escandon; 1999 #17]
DRB1*1501; DRB5*0101; DQA1*0102; DQB1*0401	Japanese	Leprosy per se	Ca = 93; Co = 114	Increased Frequency	2000	[Joko; 2000 #64]
DRB1*0405; DQA1*03; DQB1*0401	Japanese	Leprosy per se	Ca = 93; Co = 114	Decreased Frequency	2000	[Joko; 2000 #64]
DRB1*16	Polish	PTB	Ca = 31; Co = 58	RR = 9.7;p < 0.01	2000	[Dubaniewicz; 2000 #19]
DRB1*13	Polish	PTB	Ca = 31; Co = 58	RR = 0.04; p < 0.001	2000	[Dubaniewicz; 2000 #19]
DQB1; DQA1; DRB1	Brazilian	Leprosy per se	76 Pedigrees (1166 Ind)	LOD = 4.87 – 5.78	2001	[Shaw; 2001 #65]
DRB1*1501	Indian	PTB	Ca = 22; Co = 36	p < 0.05	2001	[Sriram; 2001 #92]
DRB1*15	Chinese	PTB	Ca = 74; Co = 90	RR = 2.91; p < 0.05	2001	[Wang; 2001 #21]
DRB1*11	Chinese	PTB	Ca = 74; Co = 90	RR = 0.12; p < 0.05	2001	[Wang; 2001 #21]
DR2 & DQ1	Egyptian	Leprosy per se	Ca = 24; Co = 154	OR > 3.33; p < 0.05	2002	[Hegazy; 2002 #67]
DQ1; DQ3	Turkish	Leprosy per se	Ca = 80; Co = 120	OR > 2.72; p < 0.002	2002	[Kocak; 2002 #54]
DQB1*0502	Thai	PTB	Ca = 82; Co = 160	OR = 2.06, p = 0.01 (pc = 0.13)	2002	[Vejbaesya, 2002 #157]
DQA1*0601, DQB1*0301	Thai	PTB	Ca = 82; Co = 160	OR = 0.4, p < 0.02	2002	[Vejbaesya, 2002 #157]
DQB1*05	Polish	PTB	Ca = 38; Co = 58	OR = 2.84; pc = 0.002	2003	[Dubaniewicz; 2003 #20]
DQB1*02	Polish	PTB	Ca = 38; Co = 58	OR = 0.39; p = 0.01	2003	[Dubaniewicz; 2003 #20]
DRB1; DQA1; DQB1	Mexican	LL Type	Ca = 114; Co = 204	OR > 2.73; p < 0.001	2004	[Gorodezky; 2004 #102]
QAP; QBP	Mexican	LL Type	Ca = 114; Co = 204	OR > 4.54; p < 0.007	2004	[Gorodezky; 2004 #102]
DRB1*07; DQA1*0101	Iranian	PTB	Ca = 40; Co = 100	OR = 2.7; OR = 2.66	2004	[Amirzargar; 2004 #112]
DQA1*0301; DQA1*0501	Iranian	PTB	Ca = 40; Co = 100	OR = 0.25; OR = 0.53	2004	[Amirzargar; 2004 #112]
DR4	Italian	Cavitary TB	Ca = 54; Co = 1089	RR = 2.7; p = 0.001	2004	[Ruggiero; 2004 #110]
DRB1 *13 & DRB1*14	Tuvinian Russian	PTB	14 Pedigrees	Transmitted more frequently	2005	[Pospelova, 2005 #308]

On-line Supplementary Table 2 - Mycobacterial Diseases

**MHC Class III Region:**						
C4B*QO	Brazilian	LL Type vs. ENL	Ca = 109; Co = 172	RR = 5.3	1993	[de Messias; 1993 #74]
TNF –238	Gambian	PTB	Ca = 206; Co = 229	OR = 2.54; p = 0.00001	1996	[Hill; 1996 #22]
TNF –308A	Indian	LL Type	Ca = 121; Co = 160	OR = 3.0; p = 0.02	1997	[Roy; 1997 #69]
HSP70-1A	Asian Indian	TT Type	N/A	RR = 4.58; p < 0.03	2000	[Rajalingam; 2000 #75]
HSP70-1A	Indian (North)	DR15 -ve PTB	N/A	RR = 12.6; p = 0.02	2000	[Rajalingam; 2000 #75]
TNF –308G	Brazilian	Leprosy per se	Ca = 300; Co = 92	χ^2 = 7.55; p = 0.005	2000	[Sarno; 2000 #70]
LTA	Brazilian	Leprosy per se	76 Pedigrees (1166 Ind)	LOD = 1.94	2001	[Shaw; 2001 #65]
TNF –308G	Brazilian	Leprosy per se	76 Pedigrees (1166 Ind)	LOD = 4.00	2001	[Shaw; 2001 #65]
TNF –308A	Brazilian	TT vs. LL Type	Ca = 90; Co = 92	OR = 1.65; p < 0.05	2002	[Santos; 2002 #72]
TNF	Vietnamese	Leprosy Subtypes	20 Pedigrees (118 sibs)	ZMLB = 3.52; p = 0.0002	2003	[Mira; 2003 #73]
TNF	Sicilian	PTB	Ca = 45; Co = 100	p = 0.05	2003	[Scola, 2003 #154]
LTA (5' UTR m/sat)	Malawian	Leprosy per se	Ca = 270; Co = 452	OR = 1.6; p = 0.03	2004	[Fitness; 2004 #126]
TNF (-308G; -238A)	Colombian	PTB	Ca = 135; Co = 430	OR = 1.8; OR = 2.2	2005	[Correa; 2005 #120]
**SLC11A1 (formerly NRAMP1):**						
SLC11A1 (region)	Brazilian	PTB	37 Pedigrees (287 Ind)	LOD = 0.51; p = 0.025	1997	[Shaw; 1997 #1]
SLC11A1 (region)	South Asian	Leprosy per se	20 pedigrees (168 Ind)	p < 0.02	1998	[Abel; 1998 #76]
SLC11A1 (GT(n); INT4; D543N & TGTG+/d)	Gambian	Smear +ve PTB	Ca = 410; Co = 417	OR = 4.07; p < 0.001	1998	[Bellamy; 1998 #2]
SLC11A1 (region)	Vietnamese	Mitsuda Reaction	20 pedigrees (118 Ind)	ZMLB = 3.06; p = 0.001	2000	[Alcais; 2000 #77]
SLC11A1 (INT4)	Guinea-Conakry	PTB	44 Families (160 Ind)	χ^2 = 4.14; p = 0.036	2000	[Cervino; 2000 #6]
GT(n) & D543N	Japanese	PTB	Ca = 202; Co = 267	OR = 2.07; p = 0.0003	2000	[Gao; 2000 #4]
SLC11A1 (D2S424 (distal to SLC11A1))	Canadian Indian	PTB	1 Family (81 Ind)	LOD = 3.81; p = 0.00001	2000	[Greenwood; 2000 #3]
SLC11A1 (3' UTR)	Korean	PTB	Ca = 192; Co = 192	OR = 1.85; p = 0.02	2000	[Ryu; 2000 #5]
SLC11A1 (3' UTR)	Mali	LL vs. TT Type	Ca = 273; Co = 201	OR = 5.79; p = 0.003	2001	[Meisner; 2001 #78]
SLC11A1 ($GT_{(n)}$)	Gambian	PTB	Ca = 329; Co = 324	OR = 1.40; p = 0.024	2002	[Awomoyi; 2002 #8]
SLC11A1 (D543N & TGTG+/del)	Cambodian	PTB	Ca = 358; Co = 106	OR = 0.59; p = 0.02	2002	[Delgado; 2002 #98]
SLC11A1 ($GT_{(n)}$)	Caucasian US	PTB	Ca = 135; Co = 108	OR = 2.02	2002	[Ma; 2002 #7]
SLC11A1 (INT4)	Danish	Microscopy +ve TB	Ca = 104; Co = 176	RR = 1.9; p = 0.013	2002	[Soborg, 2002 #162]
SLC11A1 (D543N)	Japanese	Cavitary Lesion in TB	Ca = 95; Co = 90	OR = 5.16	2003	[Abe; 2003 #11]
SLC11A1 (TGTG+/del)	Chinese Han	PTB	Ca = 147; Co = 145	χ^2 = 7.79; p < 0.01	2003	[Duan; 2003 #10]
SLC11A1 (D543N & TGTG+/del)	Chinese Han	PTB	Ca = 110; Co = 180	OR = 1.93; OR = 2.22	2003	[Liu; 2003 #9]
SLC11A1 ($GT_{(23)}$)	Brazilian	Mitsuda Reaction in Leprosy	Ca = 90; Co = 61	OR = 8.09	2004	[Ferreira; 2004 #125]
SLC11A1 (CAAA+/del)	Malawian	HIV -ve/+ve PTB	Ca = 239/259; Co = 762	OR = 0.65; OR = 0.70	2004	[Fitness; 2004 #127]
SLC11A1 (GT(9) & TGTG+/del)	South African	PTB	Ca = 265; Co = 224	p = 0.002 & p = 0.013	2004	[Hoal, 2004 #591]
SLC11A1 (D543N; TGTG+/del)	Chinese Han	PTB	Ca = 120; Co = 240	OR = 2.59; OR = 1.89	2004	[Liu; 2004 #104]
SLC11A1 (INT4 + D543N)	Korean	NTM Lung Disease	Ca = 41; Co = 50	OR = 10.88; p = 0.04	2005	[Koh; 2005 #132]
SLC11A1 (INT4 & D543N)	Chinese	Severe TB	Ca = 127; Co = 91	OR = 2.29; OR = 2.27	2005	[Zhang; 2005 #153]
SLC11A1 ($GT_{(n)}$ & 274C/T)	Houston (USA)	Pediatric TB	184 Nuclear Families	p = 0.04 & OR = 1.75; p = 0.01	2005	[Malik, 2005 #310]

On-line Supplementary Table 2 - Mycobacterial Diseases

**Other Candidates:**						
BCHE (cholinesterase)	Russian	PTB	N/A	RR = 6.92	1987	[Gadzhiev, 1987 #172]
CCL18 (rs2015086; rs14304)	Brazilian	PTB	92 Pedigrees (627 Ind)	RR = 0.4; RR = 0.38	2004	[Jamieson; 2004 #38]
CCL2 (-2518G)	Mexican	PTB	Ca = 445; Co = 334	OR = 2.43; p = 0.0003	2005	[Flores-Villanueva, 2005 #312]
CCL2 (-2518G)	Korean	PTB	Ca = 129; Co = 162	OR = 2.63; p = 0.0001	2005	[Flores-Villanueva, 2005 #312]
CCL4 (rs1719144)	Brazilian	PTB	92 Pedigrees (627 Ind)	RR = 0.35; p = 0.002	2004	[Jamieson; 2004 #38]
COL3A	Indian	LL Type	Ca = 25; Co = 13	RR = 5.5; p < 0.05	1997	[Kaur; 1997 #83]
CR1 (K1590E)	Malawian	Leprosy *per se*	Ca = 270; Co = 452	OR = 0.3; p = 0.02	2004	[Fitness; 2004 #126]
CR1 (Q1022H)	Malawian	HIV-ve PTB	Ca = 196; Co = 670	OR = 3.12; p = 0.03	2004	[Fitness; 2004 #127]
CTLA4	Indian	Leprosy per se	Ca = 25; Co = 13	RR = 25.83; p < 0.0065	1997	[Kaur; 1997 #83]
ESD (Esterase)	Tuvinian Russian	PTB	Ca = 73; Co = 251	N/A	1993	[Matrakshin, 1993 #166]
Haptoglobin (2:2)	Russian	PTB	Ca = 223; Co = 567	Significantly Increased	1990	[Kharakter Zh, 1990 #169]
IFNG (874 TT)	Sicilian	PTB	Ca = 45; Co = 97	p = 0.02	2002	[Lio; 2002 #32]
IFNG	Brazilian	TT Type	Ca = 192; Co = 196	p = 0.013	2003	[Reynard; 2003 #82]
IFNG	Caucasian	Smear +ve PTB	Ca = 113; Co = 207	OR = 3.75; 0.0017	2003	[Lopez-Maderuelo; 2003 #33]
IFNG	South African	PTB	Ca = 313; Co = 235	OR = 1.64; p = 0.0055	2003	[Rossouw; 2003 #89]
IFNG (874T/A)	Hong Kong Chinese	PTB	Ca = 385; Co = 451	OR = 2.24; p < 0.001	2005	[Tso; 2005 #150]
IFNG (874T/A)	Colombian	TB	Ca = 190; Co = 135	p = 0.01	2005	[Henao; 2005 #130]
IFNGR1	Croatian	PTB	Ca = 120; Co = 87	p = 0.02	2003	[Fraser; 2003 #34]
IL10 (-1082)	Cambodian	PTB	Ca = 358; Co = 106	OR = 1.84; p = 0.01	2002	[Delgado; 2002 #98]
IL10 (-8199TT)	Brazilian	TT vs. LL Type	Ca = 222; Co = 62	OR = 2.28; p < 0.01	2002	[Santos; 2002 #72]
IL10 (-1082A)	Sicilian	PTB	Ca = 45; Co = 100	p = 0.05	2003	[Scola, 2003 #154]
IL10 (-1082)	Malawian	HIV+ve PTB	Ca = 155; Co = 541	OR = 0.37; p = 0.007	2004	[Fitness; 2004 #127]
IL10 (-3575T -2849A -2763C)	Brazilian	Leprosy per se	Ca = 297; Co = 283	OR = 2.37; p = 0.027	2004	[Moraes; 2004 #136]
IL10 (-3575T -2849G -2763C -1082A -819C -592C)	Indian	Leprosy per se	Ca = 282; Co = 266	OR = 0.58; p = 0.01	2005	[Malhotra, 2005 #304]
IL12RB2 (-1035; -1023; -650; -464)	Japanese	LL vs TT Leprosy	Ca = 176; Co = 68	OR = 2.95 – 3.97	2005	[Ohyama; 2005 #139]
IL1B (-511C)	Gambian	PTB	Ca = 335; Co = 298	OR = 0.58; p = 0.015	2005	[Awomoyi, 2005 #307]
IL1RA	Gambian	PTB	Ca = 404; Co = 417	OR = 0.46; p = 0.032	1998	[Bellamy; 1998 #26]
IL8 (-251A)	Caucasian	PTB	Ca = 106; Co = 107	OR = 3.41; p < 0.006	2003	[Ma; 2003 #27]
IL8 (-251A)	African American	PTB	Ca = 180; Co = 167	OR = 3.46; p < 0.01	2003	[Ma; 2003 #27]
LAMA2	Indonesian	TT vs. LL type	Ca = 53; Co = 58	OR = 6.73; p < 0.005	2002	[Wibawa; 2002 #80]
MBP (C)	Gambian	PTB	Ca = 397; Co = 422	OR = 0.79; p = 0.037	1998	[Bellamy; 1998 #29]
MBP (B)	South African	TB Meningitis	Ca = 91; Co = 79	p = 0.017	1999	[Hoal-Van Helden; 1999 #31]
MBP (B; C; D)	Indian	PTB	Ca = 202; Co = 109	OR = 6.5; p = 0.008	1999	[Selvaraj; 1999 #30]
MBP (B; C)	West African	TB Incidence	626 Individuals	r = 0.565; t = 2.273	2003	[Mombo, 2003 #158]
MBP (B; C; D & X)	Danish	PTB	Ca = 59; Co = 250	p = 0.03	2003	[Soborg, 2003 #161]
MBP (B)	African Ameican	PTB	Ca = 176; Co = 71	OR = 0.34; p < 0.01	2004	[El Sahly; 2004 #124]
MBP (C)	Malawian	HIV+ve PTB	Ca = 154; Co = 546	OR = 1.69; p = 0.034	2004	[Fitness; 2004 #127]
NOS2A (-1026)	Brazilian	PTB	92 Pedigrees (627 Ind)	RR = 3.25; p = 0.021	2004	[Jamieson; 2004 #38]
P2RX7	Gambian	PTB	Ca = 646; Co = 694	OR = 0.70; p = 0.003	2002	[Li; 2002 #35]
PARK2/ PACRG	Vietnamese	Leprosy per se	197 pedigrees	OR = 5.28; p = 0.0005	2004	[Mira; 2004 #84]
PARK2/ PACRG	Brazilian	Leprosy per se	Ca = 587; Co = 388	OR = 2.21; p < 0.00002	2004	[Mira; 2004 #84]
PGM1 (Phosphoglucomutase)	Indian	PTB	N/A	Significant Difference	1983	[Papiha, 1983 #173]
PGM1 (*2+ allele)	South Indian	PTB	Ca = 204; Co = ?	N/A	1987	[Papiha, 1987 #174]

On-line Supplementary Table 2 - Mycobacterial Diseases

SP-A1	Mexican	PTB	Ca = 107; Co = 101	OR = 4.51, p = 0.008	2000	[Floros, 2000 #156]
SP-A2	Mexican	PTB	Ca = 107; Co = 102	OR = 9.57, p = 0.38	2000	[Floros, 2000 #156]
SP-A2 (A1660G; G1649C)	Indian	PTB	Ca = 17; Co = 19	OR = 16.3; p< 0.001	2002	[Madan; 2002 #36]
SP-A1 (307A, 776T)	Ethiopian	PTB	181 Pedigrees (226 cases)	p < 0.019	2005	[Malik, 2005 #309]
SP-A2 (355C, 751C)	Ethiopian	PTB	181 Pedigrees (226 cases)	p < 0.042	2005	[Malik, 2005 #309]
SP-B (B1012_A, AAGG_1)	Mexican	PTB	Ca = 107; Co = 103	OR = 2.36; OR = 0.12	2000	[Floros, 2000 #156]
STAT5B (rs2230097)	Brazilian	PTB	92 Pedigrees (627 Ind)	RR = 0.36; p = 0.038	2004	[Jamieson; 2004 #38]
TLR2 (Arg753Gln)	Turkish	PTB	Ca = 151; Co = 116	1.60 - 6.04 Fold Increase	2004	[Ogus; 2004 #39]
TLR2 (C2029T)	Tunisian	PTB	Ca = 33; Co = 33	p < 0.0001	2004	[Ben-Ali; 2004 #108]
TLR2 (psuedogene?)	Korean	LL Type	Ca = 131; Co = 45	22% of LL cases	2001	[Kang; 2001 #81]
UBE3A	African	PTB	180 Pedigrees	χ^2 = 4.17; p = 0.03	2002	[Cervino; 2002 #37]
VDR (tt)	Bengali Indian	TT Type	Ca = 231; Co = 166	OR = 3.22; p < 0.001	1999	[Roy; 1999 #79]
VDR (TT)	Bengali Indian	LL Type	Ca = 231; Co = 166	OR = 1.67; p = 0.03	1999	[Roy; 1999 #79]
VDR (tt)	Gambian	PTB	Ca = 408; Co = 414	OR = 0.53; p = 0.01	1999	[Bellamy; 1999 #23]
VDR (ff)	Gujarati Asian	Extra-pulmonary TB	Ca = 52; Co = 116	OR = 2.8	2000	[Wilkinson; 2000 #24]
VDR (ff)	Chinese Han	PTB	Ca = 76; Co = 171	OR = 3.67	2003	[Liu; 2003 #25]
VDR (BsmI; FokI)	Indian	Spinal TB	Ca = 64; Co = 103	OR = 2.2; OR = 2.4	2004	[Selvaraj; 2004 #146]
VDR (–ff)	Chinese Han	PTB	Ca = 120; Co = 240	OR = 2.35; p = 0.03	2004	[Liu; 2004 #104]
VDR (FokI; BsmI; ApaI; TaqI)	West African	PTB	382 Trios	χ^2 = 22.11; p = 0.009	2004	[Bornman; 2004 #116]
VDR (TaqI)	Malawian	Leprosy per se	Ca = 270; Co = 452	OR = 4.3; p = 0.004	2004	[Fitness; 2004 #126]
VDR (TaqI; FokI)	Peruvian	TB Treatment	Ca = 103; Co = 206	RR = 5.6; RR = 9.6	2004	[Roth; 2004 #144]

On-line Supplementary Table 2 - Mycobacterial Diseases

Papers Reporting No Significant Linkage or Association						
**Candidate Gene**	**Population**	**Phenotype**	**Sample Size**	**Reported Results**	**Year**	**Reference**
**MHC Class I Region:**						
HLA -A; -B	Thai	Leprosy per se	Ca = 170; Co = 100	ns	1979	[Chiewsilp, 1979 #187]
HLA -A; -B; -C	Japanese	Leprosy per se	Ca = 54; Co = 167	ns	1981	[Miyanaga, 1981 #189]
Aw30; Aw33; B7; B15; B17	Mexican American	PTB	Ca = 100; Co = 100	ns	1982	[Cox; 1982 #43]
HLA-A, -B, -C	Northern Indian	PTB	Ca = 124; Co = 109	ns	1983	[Singh, 1983 #176]
HLA	Indian	PTB	21 Families	No Linkage	1984	[Singh, 1984 #175]
HLA-A & -B	Hong Kong Chinese	PTB	Ca = 256; Co = 100	ns	1988	[Hawkins; 1988 #44]
HLA	Brazilian	PTB	98 Pedigrees (704 Ind)	ns	2001	[Blackwell; 2001 #45]
HLA –A; -B; -C	Italian	Current TB	Ca = 68; Co = 1089	ns	2004	[Ruggiero; 2004 #110]
**MHC Class II Region:**						
DR2	Northern Indian	PTB	Ca = 124; Co = 109	ns (after correction)	1983	[Singh, 1983 #176]
DRB; DQA, DQB	South Indian	PTB	Ca = 38; Co = 36; 12 families	No association; No linkage	1992	[Sanjeevi, 1992 #167]
DQB1*0501	Iranian	PTB	Ca = 40; Co = 100	ns	2004	[Amirzargar; 2004 #112]
**MHC Class III Region:**						
TNF	Polynesian	Leprosy per se	6 Pedigrees	ns	1997	[Levee; 1997 #97]
TNF	Brazilian	PTB	37 pedigrees (287 Ind)	LOD = 0.01	1997	[Shaw; 1997 #1]
TNF	Cambodian	PTB	Ca = 126; Co = 88	ns	1998	[Goldfeld; 1998 #16]
TNF -238; -308	Indian	PTB	Ca = 210; Co = 120	ns	2001	[Selvaraj; 2001 #51]
LT	Indian	PTB	Ca = 210; Co = 120	ns	2001	[Selvaraj; 2001 #51]
TNF -1030; -862; -856; -307	Cambodian	PTB	Ca = 358; Co = 106	p > 0.05	2002	[Delgado; 2002 #98]
TNF (-308; -238)	Brazilian	LL vs TT Leprosy	LL = 401; TT = 230	ns	2004	[Vanderborght; 2004 #152]
TNF (-238; -308; -376; -893)	Malawian	Leprosy per se	Ca = 270; Co = 452	ns	2004	[Fitness; 2004 #126]
TNF (-238; -308; -376; -893)	Malawian	HIV -ve/+ve PTB	Ca ~ 181/144; Co = 417	ns	2004	[Fitness; 2004 #127]
LTA (5' UTR m/sat)	Malawian	HIV -ve/+ve PTB	Ca = 198/237; Co = 707	ns	2004	[Fitness; 2004 #127]
TNF (-308)	Colombian	TB	Ca = 190; Co = 135	ns	2005	[Henao; 2005 #130]
**SLC11A1 (formerly NRAMP1):**						
SLC11A1	Polynesian	Leprosy per se	7 pedigrees (84 Ind)	ns	1997	[Roger; 1997 #86]
SLC11A1	Indian	Leprosy per se	Ca = 220; Co = 162	p = 0.80	1999	[Roy; 1999 #79]
SLC11A1	Brazilian	Mitsuda Reaction	33 ASPs (8 Ca)	ns	2001	[Hatagima; 2001 #87]
SLC11A1	Russian (Slavic)	PTB	Ca = 58; Co = 127	ns	2002	[Puzyrev; 2002 #40]
SLC11A1	Taiwanese	PTB	Ca = 49; Co = 48	ns	2002	[Liaw; 2002 #41]
SLC11A1	Moroccan	PTB	116 Pedigrees	ns	2003	[El Baghdadi; 2003 #42]
SLC11A1	Malawian	Leprosy per se	Ca = 270; Co = 452	ns	2004	[Fitness; 2004 #126]
SLC11A1 (1703G/A)	Japanese	PTB	Ca = 114, Co = 110	p = 0.144	2004	[Akahoshi; 2004 #101]
SLC11A1	Polish	PTB	Ca = 85; Co = 93	ns	2005	[Dubaniewicz; 2005 #123]

On-line Supplementary Table 2 - Mycobacterial Diseases

**Other Candidates:**						
CCL3 (-906 promoter m/sat)	Malawian	Leprosy per se	Ca = 270; Co = 452	ns	2004	[Fitness; 2004 #126]
CCL3 (-906 promoter m/sat)	Malawian	HIV -ve/+ve PTB	Ca = 147/206; Co = 580	ns	2004	[Fitness; 2004 #127]
CCL3 (-459)	Mexican	PTB	Ca = 445; Co = 518	ns	2005	[Flores-Villanueva, 2005 #312]
CCL5 (-471)	Mexican	PTB	Ca = 445; Co = 518	ns	2005	[Flores-Villanueva, 2005 #312]
CD14 -159CT	Columbian	PTB	Ca = 267; Co = 112	ns	2004	[Pacheco; 2004 #105]
CXCR1	Caucasian US	PTB	Ca = 106; Co = 107	ns	2003	[Ma; 2003 #27]
CXCR1	African American	PTB	Ca = 180; Co = 167	ns	2003	[Ma; 2003 #27]
CXCR2	Caucasian US	PTB	Ca = 106; Co = 107	ns	2003	[Ma; 2003 #27]
CXCR2	African American	PTB	Ca = 180; Co = 167	ns	2003	[Ma; 2003 #27]
Haptoglobin (HP)	Zimbabwean	PTB	Ca = 98; Co = 98	p = 0.5	2000	[Kasvosve, 2000 #155]
ICAM1 (179A/T)	Malawian	Leprosy per se	Ca = 270; Co = 452	ns	2004	[Fitness; 2004 #126]
ICAM1 (179A/T)	Malawian	HIV -ve/+ve PTB	Ca = 209/217; Co = 596	ns	2004	[Fitness; 2004 #127]
IFNA17 (551T/G)	Japanese	PTB	Ca = 114, Co = 110	p = 0.155	2004	[Akahoshi; 2004 #101]
IFNB (153C/T)	Japanese	PTB	Ca = 114, Co = 110	p = 0.137	2004	[Akahoshi; 2004 #101]
IFNG (1348T/A)	Japanese	PTB	Ca = 114, Co = 110	p = 0.55	2004	[Akahoshi; 2004 #101]
IFNG (874T/A)	Malawian	Leprosy per se	Ca = 270; Co = 452	ns	2004	[Fitness; 2004 #126]
IFNG (874T/A)	Malawian	HIV -ve/+ve PTB	Ca = 213/238; Co = 703	ns	2004	[Fitness; 2004 #127]
IFNGR1	Korean	LL Type	Ca = 93; Co = 94	ns	2003	[Lee; 2003 #88]
IFNGR1	Gambian	PTB	Ca = 297; Co = 285	ns	2004	[Awomoyi, 2004 #592]
IFNGR1 (167T/C)	Japanese	PTB	Ca = 114, Co = 110	p = 0.213	2004	[Akahoshi; 2004 #101]
IFNGR1 (-611; -56)	African American	PTB	Ca = 76; Co = 114	ns	2004	[Rosenzweig; 2004 #143]
IFNGR1 (-611; -56)	Caucasian	PTB	Ca = 70; Co = 128	ns	2004	[Rosenzweig; 2004 #143]
IFNGR1 (395)	Iranian	PTB	Ca = 50; Co = 54	ns	2005	[Mirsaeidi, 2005 #311]
IFNGR2 (839G/A)	Japanese	PTB	Ca = 114, Co = 110	p = 0.498	2004	[Akahoshi; 2004 #101]
IL10	Gambian	PTB	Ca = 792; Co = 816	ns	1998	[Bellamy; 1998 #26]
IL10	Caucasian	PTB	Ca = 113; Co = 207	ns	2003	[Lopez-Maderuelo; 2003 #33]
IL10 (-592; -819; -1082)	Malawian	Leprosy per se	Ca = 270; Co = 452	ns	2004	[Fitness; 2004 #126]
IL12 - 3’ UTR	Russian	PTB	Ca = 58; Co = 127	ns	2002	[Puzyrev; 2002 #40]
IL12p40	Caucasian US	PTB	Ca = 106; Co = 107	ns	2003	[Ma; 2003 #49]
IL12p40	African American	PTB	Ca = 180; Co = 167	ns	2003	[Ma; 2003 #49]
IL12RB1	Korean	LL Type	Ca = 93; Co = 94	ns	2003	[Lee; 2003 #88]
IL12RB1 (641A/G)	Japanese	PTB	Ca = 114, Co = 110	p = 0.610	2004	[Akahoshi; 2004 #101]
IL12RB1 (+705, +1158, +1196, +1637, +1664)	Koreans	PTB	Ca = 115; Co = 151	ns	2005	[Lee, 2005 #305]
IL12RB2 (365C/T)	Japanese	PTB	Ca = 114, Co = 110	p = 0.59	2004	[Akahoshi; 2004 #101]
IL1RA	Gujarati Asian	PTB	Ca = 89; Co = 114	ns	1999	[Wilkinson; 1999 #47]
IL1RA	Indian	PTB	Ca = 202; Co = 109	ns	2000	[Selvaraj; 2000 #48]
IL1RA	Cambodian	PTB	Ca = 358; Co = 106	χ^2 = 3.27; p = 0.19	2002	[Delgado; 2002 #98]
IL1RN	Gambian	PTB	Ca = 35; Co = 298	ns	2005	[Awomoyi, 2005 #307]
IL1β	Polynesian	Leprosy per se	6 Pedigrees	ns	1997	[Levee; 1997 #97]
IL1β	Gujarati Asian	PTB	Ca = 89; Co = 114	ns	1999	[Wilkinson; 1999 #47]
IL1β -511; +3953	Cambodian	PTB	Ca = 358; Co = 106	p = 0.32 & 0.78	2002	[Delgado; 2002 #98]
IL6 (-174G/C)	Colombian	TB	Ca = 190; Co = 135	ns	2005	[Henao; 2005 #130]

On-line Supplementary Table 2 - Mycobacterial Diseases

IL8 -251 & +781	Gambian	PTB	Ca = 284; Co = 245	p = .50; p = .42	2004	[Cooke; 2004 #106]
MBP (B; C; D)	Hispanic	PTB	Ca = 198; Co = 46	ns	2004	[El Sahly; 2004 #124]
MBP (B; C; D)	Caucasian	PTB	Ca = 113; Co = 69	ns	2004	[El Sahly; 2004 #124]
MBP (C)	Malawian	Leprosy per se	Ca = 270; Co = 452	ns	2004	[Fitness; 2004 #126]
MBP (C; D)	African American	PTB	Ca = 176; Co = 71	ns	2004	[El Sahly; 2004 #124]
MMP-1 1G/2G	Japanese	PTB	Ca = 105; Co = 106	ns	2004	[Ninomiya; 2004 #107]
NLI-IF	Caucasian US	PTB	Ca = 94; Co = 145	$p > 0.05$	2002	[Ma; 2002 #90]
NOD2	Gambian	PTB	Ca = 320; Co = 320	ns	2004	[Stockton; 2004 #109]
NOS2A (-954)	Mexican	PTB	Ca = 445; Co = 518	ns	2005	[Flores-Villanueva, 2005 #312]
SLC11A2	South African	PTB	Ca = 265; Co = 224	ns	2004	[Hoal, 2004 #591]
SPP1 (2514C/T)	Japanese	PTB	Ca = 114, Co = 110	p = 0.643	2004	[Akahoshi; 2004 #101]
TGFβ1 (T869C)	Japanese	PTB	Ca = 101; Co = 110	ns	2002	[Niimi; 2002 #137]
TGFβ1	Colombian	TB	Ca = 190; Co = 135	ns	2005	[Henao; 2005 #130]
TLR2 (Int 2 m/sat)	Malawian	Leprosy per se	Ca = 270; Co = 452	ns	2004	[Fitness; 2004 #126]
TLR2 (Int 2 m/sat)	Malawian	HIV -ve/+ve PTB	Ca = 215/249; Co = 742	ns	2004	[Fitness; 2004 #127]
TLR4	Gambian	PTB	Ca = 307; Co = 298	$\chi 2 = 0.19$; p = 1.00	2004	[Newport, 2004 #593]
TLR4 (896A/G)	Malawian	Leprosy per se	Ca = 270; Co = 452	ns	2004	[Fitness; 2004 #126]
TLR4 (896A/G)	Malawian	HIV -ve/+ve PTB	Ca = 162/120; Co = 427	ns	2004	[Fitness; 2004 #127]
TLR2	Indian	Leprosy per se	Ca = 286; Co = 183	ns	2005	[Malhotra; 2005 #135]
TNFSF5 (CD40)	West African	PTB	121 Trios	ns	2003	[Campbell; 2003 #52]
VDR	Cambodian	PTB	Ca = 358; Co = 106	$\chi 2 = 0.99$; p = 0.60	2002	[Delgado; 2002 #98]
VDR (TaqI; ApaI; BsmI)	Malawian	HIV -ve/+ve PTB	Ca ~212/225; Co = 672	ns	2004	[Fitness; 2004 #127]
**PUBMED Search Terms = Mycobacteri* AND susceptibility NOT drug; Field: Text Word, Limits: Humans**						
**PUBMED Search Term = tuberculosis AND susceptibility NOT drug; Field: Text Word, Limits: Humans**						
**PUBMED Search Terms = leprosy AND susceptibility NOT drug; Field: Text Word, Limits: Humans**						
Ca = Cases						
Co = Controls						
Ind = Individuals						
ns = Not Significant						
OR = Odds Ratio						
RR = Relative Risk						
χ^2 = Chi-Squared						
ZMLB = Z Score of the Maximum-Likelihood-Binomial						
LOD = Logarithm of the Odds						
pc = Corrected p-Value						
N/A = Not Available (Possibly Abstract Only Available)						
LL = Lepromatous Leprosy						
TT = Tuberculoid Leprosy						
TB = Tuberculosis						
PTB = Pulmonary Tuberculosis						
NTM - Non-Tuberculous Mycobacterial						

Bibliography for Webtable 2.

Abe T, Iinuma Y, Ando M, Yokoyama T, Yamamoto T, Nakashima K, Takagi N, Baba H, Hasegawa Y, Shimokata K (2003) NRAMP1 polymorphisms, susceptibility and clinical features of tuberculosis. J Infect 46:215-20

Abel L, Sanchez FO, Oberti J, Thuc NV, Hoa LV, Lap VD, Skamene E, Lagrange PH, Schurr E (1998) Susceptibility to leprosy is linked to the human NRAMP1 gene. J Infect Dis 177:133-45.

Achord AP, Lewis RE, Brackin MN, Henderson H, Cruse JM (1996) HIV-1 disease association with HLA-DQ antigens in African Americans and Caucasians. Pathobiology 64:204-8

Adje CA, Bile CE, Kestens L, Koblavi-Deme S, Ghys PD, Maurice C, Kalou-Badirou M, Kabran N, Ekpini RE, Roels TH, Wiktor SZ, Nkengasong JN (2001) Lack of effect of chemokine receptor CCR2b gene polymorphism (64I) on HIV-1 plasma RNA viral load and immune activation among HIV-1 seropositive female workers in Abidjan, Cote d'Ivoire. J Med Virol 64:398-401

Agarwal A, Guindo A, Cissoko Y, Taylor JG, Coulibaly D, Kone A, Kayentao K, Djimde A, Plowe CV, Doumbo O, Wellems TE, Diallo D (2000) Hemoglobin C associated with protection from severe malaria in the Dogon of Mali, a West African population with a low prevalence of hemoglobin S. Blood 96:2358-63

Aidoo M, McElroy PD, Kolczak MS, Terlouw DJ, ter Kuile FO, Nahlen B, Lal AA, Udhayakumar V (2001) Tumor necrosis factor-alpha promoter variant 2 (TNF2) is associated with pre-term delivery, infant mortality, and malaria morbidity in western Kenya: Asembo Bay Cohort Project IX. Genet Epidemiol 21:201-11

Aikhionbare FO, Hodge T, Kuhn L, Bulterys M, Abrams EJ, Bond VC (2001) Mother-to-child discordance in HLA-G exon 2 is associated with a reduced risk of perinatal HIV-1 transmission. Aids 15:2196-8

Aitman TJ, Cooper LD, Norsworthy PJ, Wahid FN, Gray JK, Curtis BR, McKeigue PM, Kwiatkowski D, Greenwood BM, Snow RW, Hill AV, Scott J (2000) Malaria susceptibility and CD36 mutation. Nature 405:1015-6

Akahoshi M, Ishihara M, Remus N, Uno K, Miyake K, Hirota T, Nakashima K, Matsuda A, Kanda M, Enomoto T, Ohno S, Nakashima H, Casanova JL, Hopkin JM, Tamari M, Mao XQ, Shirakawa T (2004) Association between IFNA genotype and the risk of sarcoidosis. Hum Genet

Akahoshi M, Nakashima H, Miyake K, Inoue Y, Shimizu S, Tanaka Y, Okada K, Otsuka T, Harada M (2003) Influence of interleukin-12 receptor beta1 polymorphisms on tuberculosis. Hum Genet 112:237-43

Al-Arif LI, Goldstein RA, Affronti LF, Janicki BW (1979) HLA-Bw15 and tuberculosis in a North American black population. Am Rev Respir Dis 120:1275-8

Alcais A, Sanchez FO, Thuc NV, Lap VD, Oberti J, Lagrange PH, Schurr E, Abel L (2000) Granulomatous reaction to intradermal injection of lepromin (Mitsuda reaction) is linked to the human NRAMP1 gene in Vietnamese leprosy sibships. J Infect Dis 181:302-8.

Ali S, Niang MA, N'Doye I, Critchlow CW, Hawes SE, Hill AV, Kiviat NB (2000) Secretor polymorphism and human immunodeficiency virus infection in Senegalese women. J Infect Dis 181:737-9

Allen SJ, O'Donnell A, Alexander ND, Mgone CS, Peto TE, Clegg JB, Alpers MP, Weatherall DJ (1999) Prevention of cerebral malaria in children in Papua New Guinea by southeast Asian ovalocytosis band 3. Am J Trop Med Hyg 60:1056-60

Alvarez V, Lopez-Larrea C, Coto E (1998) Mutational analysis of the CCR5 and CXCR4 genes (HIV-1 co-receptors) in resistance to HIV-1 infection and AIDS development among intravenous drug users. Hum Genet 102:483-6

Amirzargar AA, Yalda A, Hajabolbaghi M, Khosravi F, Jabbari H, Rezaei N, Niknam MH, Ansari B, Moradi B, Nikbin B (2004) The association of HLA-DRB, DQA1, DQB1 alleles and haplotype frequency in Iranian patients with pulmonary tuberculosis. Int J Tuberc Lung Dis 8:1017-21

Amodu OK, Gbadegesin RA, Ralph SA, Adeyemo AA, Brenchley PE, Ayoola OO, Orimadegun AE, Akinsola AK, Olumese PE, Omotade OO (2005) Plasmodium falciparum malaria in south-west Nigerian children: Is the polymorphism of ICAM-1 and E-selectin genes contributing to the clinical severity of malaria? Acta Trop

Amoroso A, Berrino M, Boniotto M, Crovella S, Palomba E, Scarlatti G, Serra C, Tovo PA, Vatta S (1999) Polymorphism at codon 54 of mannose-binding protein gene influences AIDS progression but not HIV infection in exposed children. Aids 13:863-4

An P, Bleiber G, Duggal P, Nelson G, May M, Mangeat B, Alobwede I, Trono D, Vlahov D, Donfield S, Goedert JJ, Phair J, Buchbinder S, O'Brien SJ, Telenti A, Winkler CA (2004) APOBEC3G genetic variants and their influence on the progression to AIDS. J Virol 78:11070-6

An P, Martin MP, Nelson GW, Carrington M, Smith MW, Gong K, Vlahov D, O'Brien SJ, Winkler CA (2000) Influence of CCR5 promoter haplotypes on AIDS progression in African-Americans. Aids 14:2117-22

An P, Nelson GW, Wang L, Donfield S, Goedert JJ, Phair J, Vlahov D, Buchbinder S, Farrar WL, Modi W, O'Brien SJ, Winkler CA (2002) Modulating influence on HIV/AIDS by interacting RANTES gene variants. Proc Natl Acad Sci U S A 99:10002-7

An P, Vlahov D, Margolick JB, Phair J, O'Brien TR, Lautenberger J, O'Brien SJ, Winkler CA (2003) A tumor necrosis factor-alpha-inducible promoter variant of interferon-gamma accelerates CD4+ T cell depletion in human immunodeficiency virus-1-infected individuals. J Infect Dis 188:228-31

Anzala AO, Ball TB, Rostron T, O'Brien SJ, Plummer FA, Rowland-Jones SL (1998) CCR2-64I allele and genotype association with delayed AIDS progression in African women. University of Nairobi Collaboration for HIV Research. Lancet 351:1632-3

Ashton LJ, Stewart GJ, Biti R, Law M, Cooper DA, Kaldor JM (2002) Heterozygosity for CCR5-Delta32 but not CCR2b-64I protects against certain intracellular pathogens. HIV Med 3:91-6

Aucan C, Walley AJ, Greenwood BM, Hill AV (2002) Haptoglobin genotypes are not associated with resistance to severe malaria in The Gambia. Trans R Soc Trop Med Hyg 96:327-8

Aucan C, Walley AJ, Hennig BJ, Fitness J, Frodsham A, Zhang L, Kwiatkowski D, Hill AV (2003) Interferon-alpha receptor-1 (IFNAR1) variants are associated with protection against cerebral malaria in the Gambia. Genes Immun 4:275-82

Aucan C, Walley AJ, Hill AV (2004) Common apolipoprotein E polymorphisms and risk of clinical malaria in the Gambia. J Med Genet 41:21-4

Awomoyi AA, Charurat M, Marchant A, Miller EN, Blackwell JM, McAdam KP, Newport MJ (2005) Polymorphism in IL1B: IL1B-511 association with tuberculosis and decreased lipopolysaccharide-induced IL-1beta in IFN-gamma primed ex-vivo whole blood assay. J Endotoxin Res 11:281-6

Awomoyi AA, Marchant A, Howson JM, McAdam KP, Blackwell JM, Newport MJ (2002) Interleukin-10, polymorphism in SLC11A1 (formerly NRAMP1), and susceptibility to tuberculosis. J Infect Dis 186:1808-14.

Awomoyi AA, Nejentsev S, Richardson A, Hull J, Koch O, Podinovskaia M, Todd JA, McAdam KP, Blackwell JM, Kwiatkowski D, Newport MJ (2004) No association between interferon-gamma receptor-1 gene polymorphism and pulmonary tuberculosis in a Gambian population sample. Thorax 59:291-4

Balamurugan A, Sharma SK, Mehra NK (2004) Human leukocyte antigen class I supertypes influence susceptibility and severity of tuberculosis. J Infect Dis 189:805-11

Balfe P, Churcher Y, Penny M, Easterbrook PJ, Goodall RL, Galpin S, Gotch F, Daniels RS, McKeating JA (1998) Association between a defective CCR-5 gene and progression to disease in HIV infection. AIDS Res Hum Retroviruses 14:1229-34

Balotta C, Bagnarelli P, Violin M, Ridolfo AL, Zhou D, Berlusconi A, Corvasce S, Corbellino M, Clementi M, Clerici M, Moroni M, Galli M (1997) Homozygous delta 32 deletion of the CCR-5 chemokine receptor gene in an HIV-1-infected patient. Aids 11:F67-71

Barbier D, Demenais F, Lefait JF, David B, Blanc M, Hors J, Feingold N (1987) Susceptibility to human cutaneous leishmaniasis and HLA, Gm, Km markers. Tissue Antigens 30:63-7

Bayoumi RA, Bashir AH, Abdulhadi NH (1986) Resistance to falciparum malaria among adults in central Sudan. Am J Trop Med Hyg 35:45-55

Beiguelman B, Alves FP, Moura MM, Engracia V, Nunes AC, Heckmann MI, Ferreira RG, da Silva LH, Camargo EP, Krieger H (2003) The association of genetic markers and malaria infection in the Brazilian Western Amazonian region. Mem Inst Oswaldo Cruz 98:455-60

Bellamy R, Kwiatkowski D, Hill AV (1998a) Absence of an association between intercellular adhesion molecule 1, complement receptor 1 and interleukin 1 receptor antagonist gene polymorphisms and severe malaria in a West African population. Trans R Soc Trop Med Hyg 92:312-6

Bellamy R, Ruwende C, Corrah T, McAdam KP, Thursz M, Whittle HC, Hill AV (1999) Tuberculosis and chronic hepatitis B virus infection in Africans and variation in the vitamin D receptor gene. J Infect Dis 179:721-4.

Bellamy R, Ruwende C, Corrah T, McAdam KP, Whittle HC, Hill AV (1998b) Assessment of the interleukin 1 gene cluster and other candidate gene polymorphisms in host susceptibility to tuberculosis. Tuber Lung Dis 79:83-9

Bellamy R, Ruwende C, Corrah T, McAdam KP, Whittle HC, Hill AV (1998c) Variations in the NRAMP1 gene and susceptibility to tuberculosis in West Africans. N Engl J Med 338:640-4.

Bellamy R, Ruwende C, McAdam KP, Thursz M, Sumiya M, Summerfield J, Gilbert SC, Corrah T, Kwiatkowski D, Whittle HC, Hill AV (1998d) Mannose binding protein deficiency is not associated with malaria, hepatitis B carriage nor tuberculosis in Africans. Qjm 91:13-8.

Ben-Ali M, Barbouche MR, Bousnina S, Chabbou A, Dellagi K (2004) Toll-Like Receptor 2 Arg677Trp Polymorphism Is Associated with Susceptibility to Tuberculosis in Tunisian Patients. Clin Diagn Lab Immunol 11:625-6

Biasin M, Boasso A, Piacentini L, Trabattoni D, Magri G, Deshmuks R, Deshpande A, Clerici M (2003) IL-4 and CXCR4 upregulation in HIV-infected and uninfected individuals from Maharashtra-Mumbai. Aids 17:1563-5

Bienzle U, Eggelte TA, Adjei LA, Dietz E, Ehrhardt S, Cramer JP, Otchwemah RN, Mockenhaupt FP (2005) Limited influence of haptoglobin genotypes on severe malaria in Ghanaian children. Trop Med Int Health 10:668-71

Bird TG, Kaul R, Rostron T, Kimani J, Embree J, Dunn PP, Bwayo JJ, Plummer FA, Rowland-Jones SL, Dong T (2002) HLA typing in a Kenyan cohort identifies novel class I alleles that restrict cytotoxic T-cell responses to local HIV-1 clades. Aids 16:1899-904

Blackwell JM (2001) Genetics and genomics in infectious disease susceptibility. Trends Mol Med 7:521-6.

Blackwell JM, Black GF, Peacock CS, Miller EN, Sibthorpe D, Gnanandha D, Shaw JJ, Silveira F, Lins-Lainson Z, Ramos F, Collins A, Shaw MA (1997) Immunogenetics of leishmanial and mycobacterial infections: the Belem Family Study. Philos Trans R Soc Lond B Biol Sci 352:1331-45

Bleiber G, May M, Suarez C, Martinez R, Marzolini C, Egger M, Telenti A (2004) MDR1 genetic polymorphism does not modify either cell permissiveness to HIV-1 or disease progression before treatment. J Infect Dis 189:583-6

Bogner JR, Lutz B, Klein HG, Pollerer C, Troendle U, Goebel FD (2004) Association of highly active antiretroviral therapy failure with chemokine receptor 5 wild type. HIV Med 5:264-72

Boniotto M, Braida L, Pirulli D, Arraes L, Amoroso A, Crovella S (2003) MBL2 polymorphisms are involved in HIV-1 infection in Brazilian perinatally infected children. Aids 17:779-80

Bornman L, Campbell SJ, Fielding K, Bah B, Sillah J, Gustafson P, Manneh K, Lisse I, Allen A, Sirugo G, Sylla A, Aaby P, McAdam KP, Bah-Sow O, Bennett S, Lienhardt C, Hill AV (2004) Vitamin D receptor polymorphisms and susceptibility to tuberculosis in West Africa: a case-control and family study. J Infect Dis 190:1631-41

Bothamley GH, Beck JS, Schreuder GM, D'Amaro J, de Vries RR, Kardjito T, Ivanyi J (1989) Association of tuberculosis and M. tuberculosis-specific antibody levels with HLA. J Infect Dis 159:549-55.

Brahmajothi V, Pitchappan RM, Kakkanaiah VN, Sashidhar M, Rajaram K, Ramu S, Palanimurugan K, Paramasivan CN, Prabhakar R (1991) Association of pulmonary tuberculosis and HLA in south India. Tubercle 72:123-32.

Bream JH, Carrington M, O'Toole S, Dean M, Gerrard B, Shin HD, Kosack D, Modi W, Young HA, Smith MW (2000) Polymorphisms of the human IFNG gene noncoding regions. Immunogenetics 51:50-8

Burgner D, Usen S, Rockett K, Jallow M, Ackerman H, Cervino A, Pinder M, Kwiatkowski DP (2003) Nucleotide and haplotypic diversity of the NOS2A promoter region and its relationship to cerebral malaria. Hum Genet 112:379-86

Cabrera M, Shaw MA, Sharples C, Williams H, Castes M, Convit J, Blackwell JM (1995) Polymorphism in tumor necrosis factor genes associated with mucocutaneous leishmaniasis. J Exp Med 182:1259-64

Cameron PU, Mallal SA, French MA, Dawkins RL (1990) Major histocompatibility complex genes influence the outcome of HIV infection. Ancestral haplotypes with C4 null alleles explain diverse HLA associations. Hum Immunol 29:282-95

Cervino AC, Lakiss S, Sow O, Bellamy R, Beyers N, Hoal-van Helden E, van Helden P, McAdam KP, Hill AV (2002) Fine mapping of a putative tuberculosis-susceptibility locus on chromosome 15q11-13 in African families. Hum Mol Genet 11:1599-603

Cervino AC, Lakiss S, Sow O, Hill AV (2000) Allelic association between the NRAMP1 gene and susceptibility to tuberculosis in Guinea-Conakry. Ann Hum Genet 64:507-12.

Chandanayingyong D, Maranetra N, Bovornkitti S (1988) HLA antigen profiles in Thai tuberculosis patients. Asian Pac J Allergy Immunol 6:77-80

Chiewsilp P, Athkambhira S, Chirachariyavej T, Bhamarapravati N, Entwistle C (1979) The HLA antigens and leprosy in Thailand. Tissue Antigens 13:186-8

Correa PA, Gomez LM, Anaya JM (2004) [Polymorphism of TNF-alpha in autoimmunity and tuberculosis]. Biomedica 24 Supp 1:43-51

Correa PA, Gomez LM, Cadena J, Anaya JM (2005) Autoimmunity and tuberculosis. Opposite association with TNF polymorphism. J Rheumatol 32:219-24

Cox RA, Arnold DR, Cook D, Lundberg DI (1982) HLA phenotypes in Mexican Americans with tuberculosis. Am Rev Respir Dis 126:653-5.

Cox RA, Downs M, Neimes RE, Ognibene AJ, Yamashita TS, Ellner JJ (1988) Immunogenetic analysis of human tuberculosis. J Infect Dis 158:1302-8

de Messias IJ, Santamaria J, Brenden M, Reis A, Mauff G (1993) Association of C4B deficiency (C4B*Q0) with erythema nodosum in leprosy. Clin Exp Immunol 92:284-7.

de Roda Husman AM, Koot M, Cornelissen M, Keet IP, Brouwer M, Broersen SM, Bakker M, Roos MT, Prins M, de Wolf F, Coutinho RA, Miedema F, Goudsmit J, Schuitemaker H (1997) Association between CCR5 genotype and the clinical course of HIV-1 infection. Ann Intern Med 127:882-90

de Sorrentino AH, Marinic K, Motta P, Sorrentino A, Lopez R, Illiovich E (2000) HLA class I alleles associated with susceptibility or resistance to human immunodeficiency virus type 1 infection among a population in Chaco Province, Argentina. J Infect Dis 182:1523-6

Delgado JC, Baena A, Thim S, Goldfeld AE (2002) Ethnic-specific genetic associations with pulmonary tuberculosis. J Infect Dis 186:1463-8

Delgado JC, Leung JY, Baena A, Clavijo OP, Vittinghoff E, Buchbinder S, Wolinsky S, Addo M, Walker BD, Yunis EJ, Goldfeld AE (2003) The -1030/-862-linked TNF promoter single-nucleotide polymorphisms are associated with the inability to control HIV-1 viremia. Immunogenetics 55:497-501

Duan HF, Zhou XH, Ma Y, Li CY, Chen XY, Gao WW, Zheng SH (2003) [A study on the association of 3'UTR polymorphisms of NRAMP1 gene with susceptibility to tuberculosis in Hans]. Zhonghua Jie He He Hu Xi Za Zhi 26:286-9

Dubaniewicz A, Jamieson SE, Dubaniewicz-Wybieralska M, Fakiola M, Nancy Miller E, Blackwell JM (2005) Association between SLC11A1 (formerly NRAMP1) and the risk of sarcoidosis in Poland. Eur J Hum Genet 13:829-34

Dubaniewicz A, Lewko B, Moszkowska G, Zamorska B, Stepinski J (2000) Molecular subtypes of the HLA-DR antigens in pulmonary tuberculosis. Int J Infect Dis 4:129-33

Dubaniewicz A, Moszkowska G, Szczerkowska Z, Hoppe A (2003) Analysis of DQB1 allele frequencies in pulmonary tuberculosis: preliminary report. Thorax 58:890-1

Easterbrook PJ, Rostron T, Ives N, Troop M, Gazzard BG, Rowland-Jones SL (1999) Chemokine receptor polymorphisms and human immunodeficiency virus disease progression. J Infect Dis 180:1096-105

El Baghdadi J, Remus N, Benslimane A, El Annaz H, Chentoufi M, Abel L, Schurr E (2003) Variants of the human NRAMP1 gene and susceptibility to tuberculosis in Morocco. Int J Tuberc Lung Dis 7:599-602

El Sahly HM, Reich RA, Dou SJ, Musser JM, Graviss EA (2004) The effect of mannose binding lectin gene polymorphisms on susceptibility to tuberculosis in different ethnic groups. Scand J Infect Dis 36:106-8

Ferreira FR, Goulart LR, Silva HD, Goulart IM (2004) Susceptibility to leprosy may be conditioned by an interaction between the NRAMP1 promoter polymorphisms and the lepromin response. Int J Lepr Other Mycobact Dis 72:457-67

Fitness J, Floyd S, Warndorff DK, Sichali L, Malema S, Crampin AC, Fine PE, Hill AV (2004a) Large-scale candidate gene study of tuberculosis susceptibility in the Karonga district of northern Malawi. Am J Trop Med Hyg 71:341-9

Fitness J, Floyd S, Warndorff DK, Sichali L, Mwaungulu L, Crampin AC, Fine PE, Hill AV (2004b) Large-scale candidate gene study of leprosy susceptibility in the Karonga district of northern Malawi. Am J Trop Med Hyg 71:330-40

Flores-Villanueva PO, Ruiz-Morales JA, Song CH, Flores LM, Jo EK, Montano M, Barnes PF, Selman M, Granados J (2005) A functional promoter polymorphism in monocyte chemoattractant protein-1 is associated with increased susceptibility to pulmonary tuberculosis. J Exp Med

Flores-Villanueva PO, Yunis EJ, Delgado JC, Vittinghoff E, Buchbinder S, Leung JY, Uglialoro AM, Clavijo OP, Rosenberg ES, Kalams SA, Braun JD, Boswell SL, Walker BD, Goldfeld AE (2001) Control of HIV-1 viremia and protection from AIDS are associated with HLA-Bw4 homozygosity. Proc Natl Acad Sci U S A 98:5140-5

Floros J, Lin HM, Garcia A, Salazar MA, Guo X, DiAngelo S, Montano M, Luo J, Pardo A, Selman M (2000) Surfactant protein genetic marker alleles identify a subgroup of tuberculosis in a Mexican population. J Infect Dis 182:1473-8

Fraser DA, Bulat-Kardum L, Knezevic J, Babarovic P, Matakovic-Mileusnic N, Dellacasagrande J, Matanic D, Pavelic J, Beg-Zec Z, Dembic Z (2003) Interferon-gamma receptor-1 gene polymorphism in tuberculosis patients from Croatia. Scand J Immunol 57:480-4

Gadzhiev BO, Sergeev AS, Movsum-zade KM (1987) [Association of mutant alleles of serum cholinesterase with various multi-factorial and infectious diseases]. Genetika 23:559-64

Gao PS, Fujishima S, Mao XQ, Remus N, Kanda M, Enomoto T, Dake Y, Bottini N, Tabuchi M, Hasegawa N, Yamaguchi K, Tiemessen C, Hopkin JM, Shirakawa T, Kishi F (2000) Genetic variants of NRAMP1 and active tuberculosis in Japanese populations. International Tuberculosis Genetics Team. Clin Genet 58:74-6.

Gaudieri S, Nolan D, McKinnon E, Witt CS, Mallal S, Christiansen FT (2005) Associations between KIR epitope combinations expressed by HLA-B/-C haplotypes found in an HIV-1 infected study population may influence NK mediated immune responses. Mol Immunol 42:557-60

Goldfeld AE, Delgado JC, Thim S, Bozon MV, Uglialoro AM, Turbay D, Cohen C, Yunis EJ (1998) Association of an HLA-DQ allele with clinical tuberculosis. Jama 279:226-8.

Gorodezky C, Alaez C, Munguia A, Cruz R, Vazquez A, Camacho A, Flores O, Rodriguez M, Rodriguez O (2004) Molecular mechanisms of MHC linked susceptibility in leprosy: towards the development of synthetic vaccines. Tuberculosis (Edinb) 84:82-92

Greenwood CM, Fujiwara TM, Boothroyd LJ, Miller MA, Frappier D, Fanning EA, Schurr E, Morgan K (2000) Linkage of tuberculosis to chromosome 2q35 loci, including NRAMP1, in a large aboriginal Canadian family. Am J Hum Genet 67:405-16.

Gyan B, Goka B, Cvetkovic JT, Perlmann H, Lefvert AK, Akanmori B, Troye-Blomberg M (2002) Polymorphisms in interleukin-1beta and interleukin-1 receptor antagonist genes and malaria in Ghanaian children. Scand J Immunol 56:619-22

Gyan BA, Goka B, Cvetkovic JT, Kurtzhals JL, Adabayeri V, Perlmann H, Lefvert AK, Akanmori BD, Troye-Blomberg M (2004) Allelic polymorphisms in the repeat and promoter regions of the interleukin-4 gene and malaria severity in Ghanaian children. Clin Exp Immunol 138:145-50

Haas DW, Wu H, Li H, Bosch RJ, Lederman MM, Kuritzkes D, Landay A, Connick E, Benson C, Wilkinson GR, Kessler H, Kim RB (2003) MDR1 gene polymorphisms and phase 1 viral decay during HIV-1 infection: an adult AIDS Clinical Trials Group study. J Acquir Immune Defic Syndr 34:295-8

Hafez M, el-Salab S, el-Shennawy F, Bassiony MR (1985) HLA-antigens and tuberculosis in the Egyptian population. Tubercle 66:35-40.

Hatagima A, Opromolla DV, Ura S, Feitosa MF, Beiguelman B, Krieger H (2001) No evidence of linkage between Mitsuda reaction and the NRAMP1 locus. Int J Lepr Other Mycobact Dis 69:99-103.

Hawkins BR, Higgins DA, Chan SL, Lowrie DB, Mitchison DA, Girling DJ (1988) HLA typing in the Hong Kong Chest Service/British Medical Research Council study of factors associated with the breakdown to active tuberculosis of inactive pulmonary lesions. Am Rev Respir Dis 138:1616-21.

Hegazy AA, Abdel-Hamid IA, Ahmed el SF, Hammad SM, Hawas SA (2002) Leprosy in a high-prevalence Egyptian village: epidemiology and risk factors. Int J Dermatol 41:681-6.

Henao MI, Montes C, Paris SC, Garcia LF (2005) Cytokine gene polymorphisms in Colombian patients with different clinical presentations of tuberculosis. Tuberculosis (Edinb)

Hill AVS, et al (1996) Association of the TNF -238 promoter polymorphism with susceptibility to tuberculosis and malaria in Africa. Human Immunology. 47:118 -

Hoal EG, Lewis LA, Jamieson SE, Tanzer F, Rossouw M, Victor T, Hillerman R, Beyers N, Blackwell JM, Van Helden PD (2004) SLC11A1 (NRAMP1) but not SLC11A2 (NRAMP2) polymorphisms are associated with susceptibility to tuberculosis in a high-incidence community in South Africa. Int J Tuberc Lung Dis 8:1464-71

Hoal-Van Helden EG, Epstein J, Victor TC, Hon D, Lewis LA, Beyers N, Zurakowski D, Ezekowitz AB, Van Helden PD (1999) Mannose-binding protein B allele confers protection against tuberculous meningitis. Pediatr Res 45:459-64.

Ifergan I, Bernard NF, Bruneau J, Alary M, Tsoukas CM, Roger M (2002) Allele frequency of three functionally active polymorphisms of the MDR-1 gene in high-risk HIV-negative and HIV-positive Caucasians. Aids 16:2340-2

Izumi S, Sugiyama K, Matsumoto Y, Ohkawa S (1982) Analysis of the immunogenetic background of Japanese leprosy patients by the HLA system. Vox Sang 42:243-7

Jamieson SE, Miller EN, Black GF, Peacock CS, Cordell HJ, Howson JM, Shaw MA, Burgner D, Xu W, Lins-Lainson Z, Shaw JJ, Ramos F, Silveira F, Blackwell JM (2004) Evidence for a cluster of genes on chromosome 17q11-q21 controlling susceptibility to tuberculosis and leprosy in Brazilians. Genes Immun 5:46-57

Jazwinska EC, Serjeantson SW (1988) HLA-DR, -DQ DNA genotyping and T-cell receptor RFLPs in leprosy. Dis Markers 6:173-83

Joko S, Numaga J, Fujino Y, Islam SM, Masuda K, Maeda H (1998) Immunogenetics of episcleritis in leprosy. Jpn J Ophthalmol 42:431-6

Joko S, Numaga J, Kawashima H, Namisato M, Maeda H (2000) Human leukocyte antigens in forms of leprosy among Japanese patients. Int J Lepr Other Mycobact Dis 68:49-56.

Joko S, Numaga J, Maeda H (1999) Immunogenetics of uveitis in leprosy. Jpn J Ophthalmol 43:97-102

Kang TJ, Chae GT (2001) Detection of Toll-like receptor 2 (TLR2) mutation in the lepromatous leprosy patients. FEMS Immunol Med Microbiol 31:53-8.

Kasvosve I, Gomo ZA, Mvundura E, Moyo VM, Saungweme T, Khumalo H, Gordeuk VR, Boelaert JR, Delanghe JR, De Bacquer D, Gangaidzo IT (2000) Haptoglobin polymorphism and mortality in patients with tuberculosis. Int J Tuberc Lung Dis 4:771-5

Kaur G, Sachdeva G, Bhutani LK, Bamezai R (1997) Association of polymorphism at COL3A and CTLA4 loci on chromosome 2q31-33 with the clinical phenotype and in-vitro CMI status in healthy and leprosy subjects: a preliminary study. Hum Genet 100:43-50.

Kharakter Zh Z, Mazhak KD, Pavlenko AV (1990) [The role of genetically determined haptoglobin phenotypes in patients with destructive pulmonary tuberculosis]. Probl Tuberk:50-2

Khomenko AG, Litvinov VI, Chukanova VP, Pospelov LE (1990) Tuberculosis in patients with various HLA phenotypes. Tubercle 71:187-92

Kikuchi M, Looareesuwan S, Ubalee R, Tasanor O, Suzuki F, Wattanagoon Y, Na-Bangchang K, Kimura A, Aikawa M, Hirayama K (2001) Association of adhesion molecule PECAM-1/CD31 polymorphism with susceptibility to cerebral malaria in Thais. Parasitol Int 50:235-9

Kim SJ, Choi IH, Dahlberg S, Nisperos B, Kim JD, Hansen JA (1987) HLA and leprosy in Koreans. Tissue Antigens 29:146-53

Kocak M, Balci M, Pence B, Kundakci N (2002) Associations between human leukocyte antigens and leprosy in the Turkish population. Clin Exp Dermatol 27:235-9.

Koh WJ, Kwon OJ, Kim EJ, Lee KS, Ki CS, Kim JW (2005) NRAMP1 Gene Polymorphism and Susceptibility to Nontuberculous Mycobacterial Lung Diseases. Chest 128:94-101

Lee HW, Lee HS, Kim DK, Ko DS, Han SK, Shim YS, Yim JJ (2005) Lack of an association between interleukin-12 receptor beta1 polymorphisms and tuberculosis in Koreans. Respiration 72:365-8

Levee G, Schurr E, Pandey JP (1997) Tumor necrosis factor-alpha, interleukin-1-beta and immunoglobulin (GM and KM) polymorphisms in leprosy. A linkage study. Exp Clin Immunogenet 14:160-5

Li CM, Campbell SJ, Kumararatne DS, Bellamy R, Ruwende C, McAdam KP, Hill AV, Lammas DA (2002) Association of a polymorphism in the P2X7 gene with tuberculosis in a Gambian population. J Infect Dis 186:1458-62.

Liaw YS, Tsai-Wu JJ, Wu CH, Hung CC, Lee CN, Yang PC, Luh KT, Kuo SH (2002) Variations in the NRAMP1 gene and susceptibility of tuberculosis in Taiwanese. Int J Tuberc Lung Dis 6:454-60.

Lio D, Marino V, Serauto A, Gioia V, Scola L, Crivello A, Forte GI, Colonna-Romano G, Candore G, Caruso C (2002) Genotype frequencies of the +874T-->A single nucleotide polymorphism in the first intron of the interferon-gamma gene in a sample of Sicilian patients affected by tuberculosis. Eur J Immunogenet 29:371-4

Liu W, Cao WC, Zhang CY, Tian L, Wu XM, Habbema JD, Zhao QM, Zhang PH, Xin ZT, Li CZ, Yang H (2004) VDR and NRAMP1 gene polymorphisms in susceptibility to pulmonary tuberculosis among the Chinese Han population: a case-control study. Int J Tuberc Lung Dis 8:428-34

Liu W, Zhang CY, Tian L, Li CZ, Wu XM, Zhao QM, Zhang PH, Yang SM, Yang H, Cao WC (2003a) [A case-control study on natural-resistance-associated macrophage protein 1 gene polymorphisms and susceptibility to pulmonary tuberculosis]. Zhonghua Yu Fang Yi Xue Za Zhi 37:408-11

Liu W, Zhang CY, Wu XM, Tian L, Li CZ, Zhao QM, Zhang PH, Yang SM, Yang H, Zhang XT, Cao WC (2003b) [A case-control study on the vitamin D receptor gene polymorphisms and susceptibility to pulmonary tuberculosis]. Zhonghua Liu Xing Bing Xue Za Zhi 24:389-92

Lopez-Maderuelo D, Arnalich F, Serantes R, Gonzalez A, Codoceo R, Madero R, Vazquez JJ, Montiel C (2003) Interferon Gamma and Interleukin-10 Gene Polymorphisms in Pulmonary Tuberculosis. Am J Respir Crit Care Med 16:16

Luoni G, Verra F, Arca B, Sirima BS, Troye-Blomberg M, Coluzzi M, Kwiatkowski D, Modiano D (2001) Antimalarial antibody levels and IL4 polymorphism in the Fulani of West Africa. Genes Immun 2:411-4

Ma X, Dou S, Wright JA, Reich RA, Teeter LD, El Sahly HM, Awe RJ, Musser JM, Graviss EA (2002) 5' dinucleotide repeat polymorphism of NRAMP1 and susceptibility to tuberculosis among Caucasian patients in Houston, Texas. Int J Tuberc Lung Dis 6:818-23.

Ma X, Reich RA, Wright JA, Tooker HR, Teeter LD, Musser JM, Graviss EA (2003) Association between interleukin-8 gene alleles and human susceptibility to tuberculosis disease. J Infect Dis 188:349-55

Madan T, Saxena S, Murthy KJ, Muralidhar K, Sarma PU (2002) Association of polymorphisms in the collagen region of human SP-A1 and SP-A2 genes with pulmonary tuberculosis in Indian population. Clin Chem Lab Med 40:1002-8
Mahmoudzadeh-Niknam H, Khalili G, Fadavi P (2003) Allelic distribution of human leukocyte antigen in Iranian patients with pulmonary tuberculosis. Hum Immunol 64:124-9
Malhotra D, Darvishi K, Sood S, Sharma S, Grover C, Relhan V, Reddy BS, Bamezai RN (2005) IL-10 promoter single nucleotide polymorphisms are significantly associated with resistance to leprosy. Hum Genet 118:295-300
Malik S, Abel L, Tooker H, Poon A, Simkin L, Girard M, Adams GJ, Starke JR, Smith KC, Graviss EA, Musser JM, Schurr E (2005a) Alleles of the NRAMP1 gene are risk factors for pediatric tuberculosis disease. Proc Natl Acad Sci U S A 102:12183-8
Malik S, Greenwood CM, Eguale T, Kifle A, Beyene J, Habte A, Tadesse A, Gebrexabher H, Britton S, Schurr E (2005b) Variants of the SFTPA1 and SFTPA2 genes and susceptibility to tuberculosis in Ethiopia. Hum Genet:1-8
Mallal S, Nolan D, Witt C, Masel G, Martin AM, Moore C, Sayer D, Castley A, Mamotte C, Maxwell D, James I, Christiansen FT (2002) Association between presence of HLA-B*5701, HLA-DR7, and HLA-DQ3 and hypersensitivity to HIV-1 reverse-transcriptase inhibitor abacavir. Lancet 359:727-32
Matrakshin AG, Tsoi KN, Pospelov LE, Kapina MA, Kholod ON, Pushkina EI (1993) [A genotypic study of children ill with tuberculosis and of healthy BCG-revaccinated ones of Tuvinian nationality]. Probl Tuberk:25-7
Matte C, Lajoie J, Lacaille J, Zijenah LS, Ward BJ, Roger M (2004) Functionally active HLA-G polymorphisms are associated with the risk of heterosexual HIV-1 infection in African women. Aids 18:427-31
Matte C, Zijenah LS, Lacaille J, Ward B, Roger M (2002) Mother-to-child human leukocyte antigen G concordance: no impact on the risk of vertical transmission of HIV-1. Aids 16:2491-4
Mehra NK, Rajalingam R, Mitra DK, Taneja V, Giphart MJ (1995) Variants of HLA-DR2/DR51 group haplotypes and susceptibility to tuberculoid leprosy and pulmonary tuberculosis in Asian Indians. Int J Lepr Other Mycobact Dis 63:241-8.
Meisner SJ, Mucklow S, Warner G, Sow SO, Lienhardt C, Hill AV (2001) Association of NRAMP1 polymorphism with leprosy type but not susceptibility to leprosy per se in west Africans. Am J Trop Med Hyg 65:733-5.
Meyer L, Magierowska M, Hubert JB, Rouzioux C, Deveau C, Sanson F, Debre P, Delfraissy JF, Theodorou I (1997) Early protective effect of CCR-5 delta 32 heterozygosity on HIV-1 disease progression: relationship with viral load. The SEROCO Study Group. Aids 11:F73-8
Minang JT, Gyan BA, Anchang JK, Troye-Blomberg M, Perlmann H, Achidi EA (2004) Haptoglobin phenotypes and malaria infection in pregnant women at delivery in western Cameroon. Acta Trop 90:107-14
Mira MT, Alcais A, Di Pietrantonio T, Thuc NV, Phuong MC, Abel L, Schurr E (2003) Segregation of HLA/TNF region is linked to leprosy clinical spectrum in families displaying mixed leprosy subtypes. Genes Immun 4:67-73.
Mira MT, Alcais A, Nguyen VT, Moraes MO, Di Flumeri C, Vu HT, Mai CP, Nguyen TH, Nguyen NB, Pham XK, Sarno EN, Alter A, Montpetit A, Moraes ME, Moraes JR, Dore C, Gallant CJ, Lepage P, Verner A, Van De Vosse E, Hudson TJ, Abel L, Schurr E (2004) Susceptibility to leprosy is associated with PARK2 and PACRG. Nature 427:636-40

Mirsaeidi SM, Houshmand M, Tabarsi P, Banoei MM, Zargari L, Amiri M, Mansouri SD, Sanati MH, Masjedi MR (2005) Lack of association between interferon-gamma receptor-1 polymorphism and pulmonary TB in Iranian population sample. J Infect

Miyanaga K, Juji T, Maeda H, Nakajima S, Kobayashi S (1981) Tuberculoid leprosy and HLA in Japanese. Tissue Antigens 18:331-4

Mohamed HS, Ibrahim ME, Miller EN, Peacock CS, Khalil EA, Cordell HJ, Howson JM, El Hassan AM, Bereir RE, Blackwell JM (2003) Genetic susceptibility to visceral leishmaniasis in The Sudan: linkage and association with IL4 and IFNGR1. Genes Immun 4:351-5

Mohamed HS, Ibrahim ME, Miller EN, White JK, Cordell HJ, Howson JM, Peacock CS, Khalil EA, El Hassan AM, Blackwell JM (2004) SLC11A1 (formerly NRAMP1) and susceptibility to visceral leishmaniasis in The Sudan. Eur J Hum Genet 12:66-74

Mombo LE, Lu CY, Ossari S, Bedjabaga I, Sica L, Krishnamoorthy R, Lapoumeroulie C (2003) Mannose-binding lectin alleles in sub-Saharan Africans and relation with susceptibility to infections. Genes Immun 4:362-7

Moraes MO, Pacheco AG, Schonkeren JJ, Vanderborght PR, Nery JA, Santos AR, Moraes ME, Moraes JR, Ottenhoff TH, Sampaio EP, Huizinga TW, Sarno EN (2004) Interleukin-10 promoter single-nucleotide polymorphisms as markers for disease susceptibility and disease severity in leprosy. Genes Immun 5:592-5

Mulherin SA, O'Brien TR, Ioannidis JP, Goedert JJ, Buchbinder SP, Coutinho RA, Jamieson BD, Meyer L, Michael NL, Pantaleo G, Rizzardi GP, Schuitemaker H, Sheppard HW, Theodorou ID, Vlahov D, Rosenberg PS (2003) Effects of CCR5-Delta32 and CCR2-64I alleles on HIV-1 disease progression: the protection varies with duration of infection. Aids 17:377-87

Nakajima S, Kobayashi S, Nohara M, Sato S (1977) HLA antigen and susceptibility to leprosy. Int J Lepr Other Mycobact Dis 45:273-7

Nakayama EE, Meyer L, Iwamoto A, Persoz A, Nagai Y, Rouzioux C, Delfraissy JF, Debre P, McIlroy D, Theodorou I, Shioda T (2002) Protective effect of interleukin-4 -589T polymorphism on human immunodeficiency virus type 1 disease progression: relationship with virus load. J Infect Dis 185:1183-6

Newport MJ, Allen A, Awomoyi AA, Dunstan SJ, McKinney E, Marchant A, Sirugo G (2004) The toll-like receptor 4 Asp299Gly variant: no influence on LPS responsiveness or susceptibility to pulmonary tuberculosis in The Gambia. Tuberculosis (Edinb) 84:347-52

Newport MJ, Awomoyi AA, Blackwell JM (2003) Polymorphism in the interferon-gamma receptor-1 gene and susceptibility to pulmonary tuberculosis in The Gambia. Scand J Immunol 58:383-5

Nguyen L, Li M, Chaowanachan T, Hu DJ, Vanichseni S, Mock PA, van Griensven F, Martin M, Sangkum U, Choopanya K, Tappero JW, Lal RB, Yang C (2004) CCR5 promoter human haplogroups associated with HIV-1 disease progression in Thai injection drug users. Aids 18:1327-33

Nieto G, Barber Y, Rubio MC, Rubio M, Fibla J (2004) Association between AIDS disease progression rates and the Fok-I polymorphism of the VDR gene in a cohort of HIV-1 seropositive patients. J Steroid Biochem Mol Biol 89-90:199-207

Ogus AC, Yoldas B, Ozdemir T, Uguz A, Olcen S, Keser I, Coskun M, Cilli A, Yegin O (2004) The Arg753GLn polymorphism of the human toll-like receptor 2 gene in tuberculosis disease. Eur Respir J 23:219-23

Ohyama H, Ogata K, Takeuchi K, Namisato M, Fukutomi Y, Nishimura F, Naruishi H, Ohira T, Hashimoto K, Liu T, Suzuki M, Uemura Y, Matsushita S (2005) Polymorphism of the 5' flanking region of the IL-12 receptor beta2 gene partially determines the clinical types of leprosy through impaired transcriptional activity. J Clin Pathol 58:740-3

Pacheco E, Fonseca C, Montes C, Zabaleta J, Garcia LF, Arias MA (2004) CD14 gene promoter polymorphism in different clinical forms of tuberculosis. FEMS Immunol Med Microbiol 40:207-13

Papiha SS, Agarwal SS, White I (1983) Association between phosphoglucomutase (PGM1) and group-specific component (Gc) subtypes and tuberculosis. J Med Genet 20:220-2

Papiha SS, Singh BN, Lanchbury JS, Roberts DF, Parsad CE, Wentzel J, Murty KJ (1987) Association of HLA and other genetic markers in South Indian patients with pulmonary tuberculosis. Tubercle 68:159-67

Pasi KJ, Sabin CA, Jenkins PV, Devereux HL, Ononye C, Lee CA (2000) The effects of the 32-bp CCR-5 deletion on HIV transmission and HIV disease progression in individuals with haemophilia. Br J Haematol 111:136-42

Peacock CS, Sanjeevi CB, Shaw MA, Collins A, Campbell RD, March R, Silveira F, Costa J, Coste CH, Nascimento MD, Siddiqui R, Shaw JJ, Blackwell JM (2002) Genetic analysis of multicase families of visceral leishmaniasis in northeastern Brazil: no major role for class II or class III regions of HLA. Genes Immun 3:350-8

Pospelov LE, Matrakshin AG, Chernousova LN, Tsoi KN, Afanasjev KI, Rubtsova GA, Yeremeyev VV (1996) Association of various genetic markers with tuberculosis and other lung diseases in Tuvinian children. Tuber Lung Dis 77:77-80

Pospelova LE, Matrashkin AG, Larionova EE, Eremeev VV, Mes'ko EM (2005) [The association of tuberculosis with the specificities of the HLA gene DRB1 in different regions of Tuva]. Probl Tuberk Bolezn Legk:23-5

Price P, James I, Fernandez S, French MA (2004) Alleles of the gene encoding IL-1alpha may predict control of plasma viraemia in HIV-1 patients on highly active antiretroviral therapy. Aids 18:1495-501

Puzyrev VP, Freidin MB, Rudko AA, Strelis AK, Kolokolova OV (2002) [Polymorphisms of the candidate genes for genetic susceptibility to tuberculosis in the Slavic population of Siberia: a pilot study]. Mol Biol (Mosk) 36:788-91.

Rajalingam R, Mehra NK, Jain RC, Myneedu VP, Pande JN (1996) Polymerase chain reaction--based sequence-specific oligonucleotide hybridization analysis of HLA class II antigens in pulmonary tuberculosis: relevance to chemotherapy and disease severity. J Infect Dis 173:669-76.

Rajalingam R, Mehra NK, Singal DP (2000) Polymorphism in heat-shock protein 70-1 (HSP70-1) gene promoter region and susceptibility to tuberculoid leprosy and pulmonary tuberculosis in Asian Indians. Indian J Exp Biol 38:658-62.

Rajalingam R, Singal DP, Mehra NK (1997) Transporter associated with antigen-processing (TAP) genes and susceptibility to tuberculoid leprosy and pulmonary tuberculosis. Tissue Antigens 49:168-72.

Rani R, Fernandez-Vina MA, Zaheer SA, Beena KR, Stastny P (1993) Study of HLA class II alleles by PCR oligotyping in leprosy patients from north India. Tissue Antigens 42:133-7.

Ravikumar M, Dheenadhayalan V, Rajaram K, Lakshmi SS, Kumaran PP, Paramasivan CN, Balakrishnan K, Pitchappan RM (1999) Associations of HLA-DRB1, DQB1 and DPB1 alleles with pulmonary tuberculosis in south India. Tuber Lung Dis 79:309-17

Remus N, El Baghdadi J, Fieschi C, Feinberg J, Quintin T, Chentoufi M, Schurr E, Benslimane A, Casanova JL, Abel L (2004) Association of IL12RB1 polymorphisms with pulmonary tuberculosis in adults in Morocco. J Infect Dis 190:580-7

Reynard MP, Turner D, Junqueira-Kipnis AP, Ramos De Souza M, Moreno C, Navarrete CV (2003) Allele frequencies for an interferon-gamma microsatellite in a population of Brazilian leprosy patients. Eur J Immunogenet 30:149-51.

Roger M, Levee G, Chanteau S, Gicquel B, Schurr E (1997) No evidence for linkage between leprosy susceptibility and the human natural resistance-associated macrophage protein 1 (NRAMP1) gene in French Polynesia. Int J Lepr Other Mycobact Dis 65:197-202.

Rossouw M, Nel HJ, Cooke GS, van Helden PD, Hoal EG (2003) Association between tuberculosis and a polymorphic NFkappaB binding site in the interferon gamma gene. Lancet 361:1871-2

Roth DE, Soto G, Arenas F, Bautista CT, Ortiz J, Rodriguez R, Cabrera L, Gilman RH (2004) Association between vitamin D receptor gene polymorphisms and response to treatment of pulmonary tuberculosis. J Infect Dis 190:920-7

Roy S, Frodsham A, Saha B, Hazra SK, Mascie-Taylor CG, Hill AV (1999) Association of vitamin D receptor genotype with leprosy type. J Infect Dis 179:187-91.

Roy S, McGuire W, Mascie-Taylor CG, Saha B, Hazra SK, Hill AV, Kwiatkowski D (1997) Tumor necrosis factor promoter polymorphism and susceptibility to lepromatous leprosy. J Infect Dis 176:530-2.

Royo JL, Ruiz A, Borrego S, Rubio A, Sanchez B, Nunez-Roldan A, Lissen E, Antinolo G (2001) Fluorescence resonance energy transfer analysis of CCR-V64I and SDF1-3'a polymorphisms: prevalence in southern Spain hiv type 1+ cohort and noninfected population. AIDS Res Hum Retroviruses 17:663-6

Ruggiero G, Cosentini E, Zanzi D, Sanna V, Terrazzano G, Matarese G, Sanduzzi A, Perna F, Zappacosta S (2004) Allelic distribution of human leucocyte antigen in historical and recently diagnosed tuberculosis patients in Southern Italy. Immunology 111:318-22

Ruiz A, Royo JL, Rubio A, Borrego S, Leal M, Sanchez B, Nunez-Roldan A, Antinolo G (2001) Spectrofluorimetric analysis of CCR5-delta 32 allele using real-time polymerase chain reaction: prevalence in southern Spanish HIV(+) patients and noninfected population. AIDS Res Hum Retroviruses 17:191-3

Ryu S, Park YK, Bai GH, Kim SJ, Park SN, Kang S (2000) 3'UTR polymorphisms in the NRAMP1 gene are associated with susceptibility to tuberculosis in Koreans. Int J Tuberc Lung Dis 4:577-80.

Saah AJ, Hoover DR, Weng S, Carrington M, Mellors J, Rinaldo CR, Jr., Mann D, Apple R, Phair JP, Detels R, O'Brien S, Enger C, Johnson P, Kaslow RA (1998) Association of HLA profiles with early plasma viral load, CD4+ cell count and rate of progression to AIDS following acute HIV-1 infection. Multicenter AIDS Cohort Study. Aids 12:2107-13

Sanjeevi CB, Narayanan PR, Prabakar R, Charles N, Thomas BE, Balasubramaniam R, Olerup O (1992) No association or linkage with HLA-DR or -DQ genes in south Indians with pulmonary tuberculosis. Tuber Lung Dis 73:280-4

Santos AR, Suffys PN, Vanderborght PR, Moraes MO, Vieira LM, Cabello PH, Bakker AM, Matos HJ, Huizinga TW, Ottenhoff TH, Sampaio EP, Sarno EN (2002) Role of tumor necrosis factor-alpha and interleukin-10 promoter gene polymorphisms in leprosy. J Infect Dis 186:1687-91.

Sarno EN, Santos AR, Jardim MR, Suffys PN, Almeida AS, Nery JA, Vieira LM, Sampaio EP (2000) Pathogenesis of nerve damage in leprosy: genetic polymorphism regulates the production of TNF alpha. Lepr Rev 71 Suppl:S154-8; discussion S158-60.

Schauf V, Ryan S, Scollard D, Jonasson O, Brown A, Nelson K, Smith T, Vithayasai V (1985) Leprosy associated with HLA-DR2 and DQw1 in the population of northern Thailand. Tissue Antigens 26:243-7.

Scola L, Crivello A, Marino V, Gioia V, Serauto A, Candore G, Colonna-Romano G, Caruso C, Lio D (2003) IL-10 and TNF-alpha polymorphisms in a sample of Sicilian patients affected by tuberculosis: implication for ageing and life span expectancy. Mech Ageing Dev 124:569-72

Selvaraj P, Kurian SM, Chandra G, Reetha AM, Charles N, Narayanan PR (2004) Vitamin D receptor gene variants of BsmI, ApaI, TaqI, and FokI polymorphisms in spinal tuberculosis. Clin Genet 65:73-6

Selvaraj P, Kurian SM, Uma H, Reetha AM, Narayanan PR (2000a) Influence of non-MHC genes on lymphocyte response to Mycobacterium tuberculosis antigens & tuberculin reactive status in pulmonary tuberculosis. Indian J Med Res 112:86-92

Selvaraj P, Narayanan PR, Reetha AM (1999) Association of functional mutant homozygotes of the mannose binding protein gene with susceptibility to pulmonary tuberculosis in India. Tuber Lung Dis 79:221-7

Selvaraj P, Narayanan PR, Reetha AM (2000b) Association of vitamin D receptor genotypes with the susceptibility to pulmonary tuberculosis in female patients & resistance in female contacts. Indian J Med Res 111:172-9.

Selvaraj P, Sriram U, Mathan Kurian S, Reetha AM, Narayanan PR (2001) Tumour necrosis factor alpha (-238 and -308) and beta gene polymorphisms in pulmonary tuberculosis: haplotype analysis with HLA-A, B and DR genes. Tuberculosis 81:335-41

Selvaraj P, Uma H, Reetha AM, Kurian SM, Xavier T, Prabhakar R, Narayanan PR (1998) HLA antigen profile in pulmonary tuberculosis patients & their spouses. Indian J Med Res 107:155-8

Shankarkumar U (2004) HLA associations in leprosy patients from Mumbai, India. Lepr Rev 75:79-85

Shankarkumar U, Ghosh K, Badakere S, Mohanty D (2003) Novel HLA Class I Alleles Associated with Indian Leprosy Patients. J Biomed Biotechnol 2003:208-211

Shaw MA, Collins A, Peacock CS, Miller EN, Black GF, Sibthorpe D, Lins-Lainson Z, Shaw JJ, Ramos F, Silveira F, Blackwell JM (1997) Evidence that genetic susceptibility to Mycobacterium tuberculosis in a Brazilian population is under oligogenic control: linkage study of the candidate genes NRAMP1 and TNFA. Tuber Lung Dis 78:35-45

Shaw MA, Donaldson IJ, Collins A, Peacock CS, Lins-Lainson Z, Shaw JJ, Ramos F, Silveira F, Blackwell JM (2001) Association and linkage of leprosy phenotypes with HLA class II and tumour necrosis factor genes. Genes Immun 2:196-204.

Shi YP, Nahlen BL, Kariuki S, Urdahl KB, McElroy PD, Roberts JM, Lal AA (2001) Fcgamma receptor IIa (CD32) polymorphism is associated with protection of infants against high-density Plasmodium falciparum infection. VII. Asembo Bay Cohort Project. J Infect Dis 184:107-11

Singh SP, Mehra NK, Dingley HB, Pande JN, Vaidya MC (1983) Human leukocyte antigen (HLA)-linked control of susceptibility to pulmonary tuberculosis and association with HLA-DR types. J Infect Dis 148:676-81

Singh SP, Mehra NK, Dingley HB, Pande JN, Vaidya MC (1984) HLA haplotype segregation study in multiple case families of pulmonary tuberculosis. Tissue Antigens 23:84-6

Soborg C, Andersen AB, Madsen HO, Kok-Jensen A, Skinhoj P, Garred P (2002) Natural resistance-associated macrophage protein 1 polymorphisms are associated with microscopy-positive tuberculosis. J Infect Dis 186:517-21

Soborg C, Madsen HO, Andersen AB, Lillebaek T, Kok-Jensen A, Garred P (2003) Mannose-binding lectin polymorphisms in clinical tuberculosis. J Infect Dis 188:777-82

Soebono H, Giphart MJ, Schreuder GM, Klatser PR, de Vries RR (1997) Associations between HLA-DRB1 alleles and leprosy in an Indonesian population. Int J Lepr Other Mycobact Dis 65:190-6.

Sriram U, Selvaraj P, Kurian SM, Reetha AM, Narayanan PR (2001) HLA-DR2 subtypes & immune responses in pulmonary tuberculosis. Indian J Med Res 113:117-24

Stockton JC, Howson JM, Awomoyi AA, McAdam KP, Blackwell JM, Newport MJ (2004) Polymorphism in NOD2, Crohn's disease, and susceptibility to pulmonary tuberculosis. FEMS Immunol Med Microbiol 41:157-60

Teran-Escandon D, Teran-Ortiz L, Camarena-Olvera A, Gonzalez-Avila G, Vaca-Marin MA, Granados J, Selman M (1999) Human leukocyte antigen-associated susceptibility to pulmonary tuberculosis: molecular analysis of class II alleles by DNA amplification and oligonucleotide hybridization in Mexican patients. Chest 115:428-33.

Todd JR, West BC, McDonald JC (1990) Human leukocyte antigen and leprosy: study in northern Louisiana and review. Rev Infect Dis 12:63-74.

Tso HW, Ip WK, Chong WP, Tam CM, Chiang AK, Lau YL (2005) Association of interferon gamma and interleukin 10 genes with tuberculosis in Hong Kong Chinese. Genes Immun 6:358-63

Uko GP, Lu LY, Asuquo MA, Fici D, Mahan S, Awdeh Z, Udim ER, Ding W, Umana U, Adewole T, Fraser PA (1999) HLA-DRB1 leprogenic motifs in nigerian population groups. Clin Exp Immunol 118:56-62.

van Eden W, de Vries RR, D'Amaro J, Schreuder I, Leiker DL, van Rood JJ (1982) HLA-DR-associated genetic control of the type of leprosy in a population from surinam. Hum Immunol 4:343-50.

van Eden W, de Vries RR, Mehra NK, Vaidya MC, D'Amaro J, van Rood JJ (1980) HLA segregation of tuberculoid leprosy: confirmation of the DR2 marker. J Infect Dis 141:693-701

van Eden W, Gonzalez NM, de Vries RR, Convit J, van Rood JJ (1985) HLA-linked control of predisposition to lepromatous leprosy. J Infect Dis 151:9-14

Vanderborght PR, Matos HJ, Salles AM, Vasconcellos SE, Silva-Filho VF, Huizinga TW, Ottenhoff TH, Sampaio EP, Sarno EN, Santos AR, Moraes MO (2004) Single nucleotide polymorphisms (SNPs) at -238 and -308 positions in the TNFalpha promoter: clinical and bacteriological evaluation in leprosy. Int J Lepr Other Mycobact Dis 72:143-8

Vejbaesya S, Chierakul N, Luangtrakool K, Srinak D, Stephens HA (2002) Associations of HLA class II alleles with pulmonary tuberculosis in Thais. Eur J Immunogenet 29:431-4

Verra F, Luoni G, Calissano C, Troye-Blomberg M, Perlmann P, Perlmann H, Arca B, Sirima BS, Konate A, Coluzzi M, Kwiatkowski D, Modiano D (2004) IL4-589C/T polymorphism and IgE levels in severe malaria. Acta Trop 90:205-9

Wang J, Song C, Wang S (2001) [Association of HLA-DRB1 genes with pulmonary tuberculosis]. Zhonghua Jie He He Hu Xi Za Zhi 24:302-5.

Wang LM, Kimura A, Satoh M, Mineshita S (1999) HLA linked with leprosy in southern China: HLA-linked resistance alleles to leprosy. Int J Lepr Other Mycobact Dis 67:403-8.

Wibawa T, Soebono H, Matsuo M (2002) Association of a missense mutation of the laminin alpha2 gene with tuberculoid type of leprosy in Indonesian patients. Trop Med Int Health 7:631-6.

Wilkinson RJ, Llewelyn M, Toossi Z, Patel P, Pasvol G, Lalvani A, Wright D, Latif M, Davidson RN (2000) Influence of vitamin D deficiency and vitamin D receptor polymorphisms on tuberculosis among Gujarati Asians in west London: a case-control study. Lancet 355:618-21.

Xu KY, de Vries RR, Fei HM, van Leeuwen A, Chen RB, Ye GY (1985) HLA-linked control of predisposition to lepromatous leprosy. Int J Lepr Other Mycobact Dis 53:56-63

Zerva L, Cizman B, Mehra NK, Alahari SK, Murali R, Zmijewski CM, Kamoun M, Monos DS (1996) Arginine at positions 13 or 70-71 in pocket 4 of HLA-DRB1 alleles is associated with susceptibility to tuberculoid leprosy. J Exp Med 183:829-36.

Zhang W, Shao L, Weng X, Hu Z, Jin A, Chen S, Pang M, Chen ZW (2005) Variants of the natural resistance-associated macrophage protein 1 gene (NRAMP1) are associated with severe forms of pulmonary tuberculosis. Clin Infect Dis 40:1232-6

Zimmerman PA, Fitness J, Moulds JM, McNamara DT, Kasehagen LJ, Rowe JA, Hill AV (2003) CR1 Knops blood group alleles are not associated with severe malaria in the Gambia. Genes Immun 4:368-73
