# Supplementary material for: Genetic susceptibility to infectious diseases: big is beautiful, but will bigger be even better?
Source: Lancet Infect Dis. 2006 Oct;6(10):653–63. doi: 10.1016/S1473-3099(06)70601-6 (PMC2330096; doi:10.1016/S1473-3099(06)70601-6)
Supplement: Supplementary file 3 [file mmc3.pdf]

On-line Supplementary Table 3 - Malaria

**Papers Reporting Significant Linkage or Association**						
**Candidate Gene**	**Population**	**Phenotype**	**Sample Size**	**Reported Results**	**Year**	**Reference**
**MHC Class I Region:**						
Bw53	West African	Severe Malaria	?	?	1991	[Hill, 1991 #278]
B53	Gambian	Severe Malarial Anaenia	SMA = 193; Co = 371	OR = 0.32; p < 0.001	1999	[McGuire, 1999 #222]
B49	Indian	Complicated Severe Malaria	Ca = 171; Co = 101	OR = 13.88; p < 0.0001	2002	[Shankarkumar, 2002 #291]
B35	Malian	Malarial Parasite Type (cp26 & cp29)	Ca = 305	OR = 0.48; p = 0.0009	2005	[Young, 2005 #285]
B46	Thai	Severe vs Cerebral Malaria	SM = 322; CM = 218	Increased in CM; p = 0.005 nc	2005	[Hananantachai, 2005 #129]
B56	Thai	Mild vs Cerebral Malaria	MM = 404; CM = 218	Increased in MM; p = 0.032 nc	2005	[Hananantachai, 2005 #129]
**MHC Class II Region:**						
DRB1*1302 – DQB1*0501	West African	Severe Malaria	?	?	1991	[Hill, 1991 #278]
DR3, DR10, DR13	Senegalese	Cerebral Malaria	Ca = 46; Co = 220	RR > 2.84; p < 0.001	1998	[Ndiaye, 1998 #303]
DRB1*04 & DPB1*1701	Gabonese	Mild vs Severe Malaria	SM = 91; MM = 88	Increased in SM; p < 0.05	1999	[May, 1999 #256]
DQB1*0501	Gabonese	Reinfection in Mild vs Severe Malaria	MM = 88; SM = 91	MM OR = 0.3; p = 0.026	2001	[May, 2001 #252]
DRB1*0809	Indian	Complicated Severe Malaria	Ca = 171; Co = 101	OR = 13.88; p < 0.0001	2002	[Shankarkumar, 2002 #291]
DRB1*1001	Thai	Mild & Cerebral Malaria	MM = 388 vs CM = 204	Increased in MM p = 0.007 nc	2005	[Hananantachai, 2005 #129]
**MHC Class III Region:**						
TNF (-308)	Gambian	Cerebral Malaria	?	RR = 7.0	1994	[McGuire, 1994 #223]
TNF (-238A)	Gambian	Severe Malarial Anaemia	SMA = 193; Co = 371	OR = 2.5; p < 0.001	1999	[McGuire, 1999 #222]
TNF (-308)	Sri Lankan	Severe Malaria	SM = 35, Co = 84	OR = 2.65; p = 0.021	1999	[Wattavidanage, 1999 #266]
TNF (-376A)	Gambian	Cerebral Malaria	CM = 384; Co = 371	OR = 4.3; p = 0.0008	1999	[Knight, 1999 #243]
TNF (-376A)	Kenyan	Cerebral Malaria	CM = 257; Co = 311	OR = 4.6; p = 0.016	1999	[Knight, 1999 #243]
TNF (-308)	Kenyan	Pre-term birth due Malaria	1048 Infants	RR = 7.3; p = 0.002	2001	[Aidoo, 2001 #272]
TNF (-857C/T)	Myanmar (Karen Ethnic Group)	Cerebral vs Mild Malaria	CM = 22; MM = 106	OR = 124.86; p < 0.001	2001	[Ubalee, 2001 #219]
TNF (-857C/T)	Myanmar (Burmese)	Cerebral vs Mild Malaria	CM = 21; MM = 94	OR = 34.50; p < 0.001	2001	[Ubalee, 2001 #219]
TNF (-308A)	Gabonese	*P. falciparum* Reinfection	SM = 98; MM = 100	Increased Reinfection in SM; p = 0.05	2002	[Meyer, 2002 #255]
TNF (TNFd marker)	Burkina Faso	Mild Malaria	34 Pedigrees; 197 Ind	MLB-LOD 3.27; p = 5.44 x 10-5	2003	[Flori, 2003 #209]
**Other Candidates:**						
ABO Blood Group (A or O)	Indian (Madhya Pradesh)	*P. falciparum* vs *P. vivax* Infection	696 Malaria Patients	*P. falciparum* Decreased in A & O	1995	[Singh, 1995 #567]
ABO blood group (A)	Gabonese	Severe vs Mild Malaria	SM = 100; MM = 100	OR = 0.3; p < 0.01	1999	[Lell, 1999 #250]
ABO Blood Group (O)	Gabonese	*P. falciparum* Parasitemia	Ca = 300	Parasitemia Lower; p = 0.043	2000	[Migot-Nabias, 2000 #230]
ABO Blood Group (O)	Gabonese	Asyptomatic *P. falciparum*	Ca = 98; Co - 60	Increased in Cases; p = 0.05	2003	[Mombo, 2003 #229]
ABO Blood Group (A &/or B)	Brazilian (Amazonian)	Reported Number of Malarial Episodes	182 Ind	H = 4.054; p = 0.044	2003	[Beiguelman, 2003 #212]
ABO Blood Groups (O)	Sri Lankan	Severe vs Uncomplicated Malaria	SM = 80; UM = 163	Decreased in SM; p = 0.0003	2005	[Pathirana, 2005 #570]
ApoE (e2/e2)	Ghanaian	Malaria - Time to infection	110 new borns	X2 = 15.69; p = 0.008	2003	[Wozniak, 2003 #287]
ApoE (e3/e4)	Gambian	Cerebral & Severe Malarial Anaemia	CM & SM = 49; Co = 560	Increased in Cases; p = 0.006	2004	[Aucan, 2004 #228]
CD31 (125V/V, 563N/N)	Thai	Cerebral vs Severe Malaria	CM = 43; SM = 89	OR = 2.92; p < 0.01	2001	[Kikuchi, 2001 #221]
CD36 (1264T/G)	Kenyan plus Gambian	Cerebral Malaria	CM = 388, Co = 761	OR = 1.49; p = 0.04	2000	[Aitman, 2000 #268]
CD36 (1264T/G)	Kenyan plus Gambian	Malaria *per se*	Ca = 598, Co = 761	OR = 1.53; p = 0.01	2000	[Aitman, 2000 #268]
CD36 (T188G)	Kenyan	Severe Malaria	SM = 693, Co = 693	OR = 0.74; p = 0.036	2001	[Pain, 2001 #235]
CD36 (539delAC)	Thai	Cerebral vs Mild Malaria	CM = 107; MM = 203	Increased in CM; p = 0.040	2002	[Omi, 2002 #216]
CD36 (Intron3 (TG)12)	Thai	Cerebral vs Mild Malaria	CM = 108; MM = 203	OR = 0.59; p = 0.0069	2003	[Omi, 2003 #215]
CD40L (-726C)	Gambian (hemizygous males)	Severe, Cerebral & Malarial Anaemia	SM = 310; CM = 232; SMA = 81; Co = 122	OR < 0.55; p < 0.01	2002	[Sabeti, 2002 #233]
CR1 (3650 AG)	Papua New Guinea (Madang)	Severe Malaria	SM = 180; Co = 179	OR = 0.33; p = 0.005	2004	[Cockburn, 2004 #558]
CR1 (sl2/2)	Kenyan	Severe Malarial Anaemia	SMA = 137; Co - 137	OR = 0.17; p = 0.02	2005	[Thathy, 2005 #559]
Duffy Blood Group (FyFy)	African American	*Plasmodium vivax* Infection	17 Ind	100% Protection	1976	[Miller, 1976 #296]
Duffy Blood Group (FyFy)	Gambian	*P. vivax* Infection	1168 Ind Screened	100% Protection	1977	[Welch, 1977 #574]
Duffy Blood Group (FyFy)	Honduras	*P. vivax* Infection	Ca = 14; Co = 406	FyFy Confers Resistance	1978	[Spencer, 1978 #573]
Duffy Blood Group Fy (a-b-)	Brazilian (Amazonian)	Self Reported Number of Malarial Episodes	182 Ind	H = 4.632; p = 0.031	2003	[Beiguelman, 2003 #212]

On-line Supplementary Table 3 - Malaria

Esterase D (EsD)	Brazilian (Amazonian)	Self Reported Number of Malarial Episodes	182 Ind	H = 6.840; p = 0.033	2003	[Beiguelman, 2003 #212]
FcγRIIa (131R/R)	Kenyan	High Density *P. falciparum* Malaria	FM = 97; Con = 85	OR = 0.278; p= 0.0021	2001	[Shi, 2001 #241]
FcγRIIa (131H/H) & FcγRIIIb (NA2)	Thai	Cerebral vs Mild Malaria	CM = 107; MM = 202	OR = 1.85; p = 0.012	2002	[Omi, 2002 #213]
FcγRIIa (131H/H)	West African	Severe vs Mild Malaria	**?**	OR - 1.40; p = 0.03	2003	[Cooke, 2003 #224]
FcγRIIa (His/His131)	West Kenyan (females)	Placental Malaria (HIV+ve only)	Ca = 151; Co = 507	OR = 1.72; p = 0.016	2004	[Brouwer, 2004 #117]
G6PD A/-	African (females & males)	Severe Malaria	**?**	46-58% Reduction in Risk	1995	[Ruwende, 1995 #299]
G6PD (A/-)	Gabonese (females)	Mild Malaria	Ca = 82; Co = 76	Increased in Cases; p = 0.026	2000	[Migot-Nabias, 2000 #230]
G6PD (A-)	Gabonese (females)	Asymptomatic *P. falciparum*	Ca = 44; Co = 29	Reduced in Cases; p = 0.03	2003	[Mombo, 2003 #229]
G6PD	Brazilian (Amazonian females)	Asyptomatic *P. vivax*	182 Ind (? Female numbers)	X2 = 4.353; p = 0.037	2003	[Beiguelman, 2003 #212]
G6PD	Ugandan	Incidence of Malaria	Ca = 184; Co = 123	IRR = 1.63; p = 0.03	2004	[Parikh, 2004 #552]
Globin (-α/αα)	Ghanaian	Severe Malaria	Ca = 301 Co = 1093	OR = 0.74; p = 0.03	2004	[Mockenhaupt, 2004 #282]
Globin (-α/αα & -α/-α)	Kenyan	Severe and Fatal Malaria	SM = 655; Co = 648	OR < 0.73; p < 0.013	2005	[Williams, 2005 #284]
Gp91phox TA(11 & 16)	Gabonese	Severe vs Mild Malaria	SM = 92; MM = 91	Increased in MM; p = 0.026	2004	[Uhlemann, 2004 #251]
Haemoglobin AS	Indian (Kheda District)	*P. falciparum* Infection	Not given - Abstract only	Significantly Decreased in Cases	1992	[Pant, 1992 #569]
Haemoglobin AS	Gabonese	Malaria Infection Rate	163 Children	Inreased Multiple Infections; p = 0.01	1997	[Ntoumi, 1997 #275]
Haemoglobin AS	Gabonese	Severe vs Mild Malaria	SM = 100; MM = 100	RR = 0.61; p = 0.045	1998	[Kun, 1998 #254]
Haemoglobin AS	Gabonese	Severe vs Mild Malaria	SM = 100; MM = 100	OR = 2.3; p = 0.04	1999	[Lell, 1999 #250]
Haemoglobin C	Malian	Cerebral Malaria vs Uncompicated Malaria	CM = 34; UM = 391	OR = 0.14; p = 0.03	2000	[Agarwal, 2000 #269]
Haemoglobin AS	Gabonese	*P. falciparum* Parasitemia	Ca = 300	Increased Parasitemia; p = 0.031	2000	[Migot-Nabias, 2000 #230]
Haemoglobin C	Malian	Severe Malaria vs Uncomplicated Malaria	CM = 67; UM = 391	OR = 0.22; p = 0.01	2001	[Agarwal, 2000 #269]
Haemoglobin AC & CC	Burkina Faso	Falciparum Malaria	Ca = 835, Co = 3513	OR < 0.71; p < 0.0011	2001	[Modiano, 2001 #270]
Haemoglobin AS	Burkina Faso	Falciparum Malaria	Ca = 835, Co = 3513	OR = 0.27; p < 0.001	2001	[Modiano, 2001 #270]
Haemoglobin C	Burkina Faso	Mild Malaria	53 Pedigrees; 256 ind; 73 Cases	Z = -3.94; p = 0.00013	2004	[Rihet, 2004 #142]
Haemoglobin A	Burkina Faso	Mild Malaria	53 Pedigrees; 256 ind; 73 Cases	Z = +4.27; p = 0.00002	2004	[Rihet, 2004 #142]
Haemoglobin AS	Ghanaian	Severe Malaria	Ca = 290; Co = 290	OR = 0.20; p = 0.01	2004	[Mockenhaupt, 2004 #283]
Haptoglobin (Hp1-1)	Sudanese	Falciparum Malaria	FM = 345; Con = 208	Hp1-1 Increased in FM; p < 0.001	1998	[Elagib, 1998 #238]
Haptoglobin (Hp1-1)	Ghanaian	Severe Malaria	SM = 113; Co = 42	43% vs 7.1%	2000	[Quaye, 2000 #249]
Haptoglobin (Hp1-1)	Cameroon	*P. falciparum* Placental Infection	98 Ca	Increased Placental Infection; p = 0.001	2004	[Minang, 2004 #248]
Haptoglobin (Hp2-2)	Ghanaian	Severe Malaria	Ca = 290; Co = 290	OR = 1.76; p = 0.04	2005	[Bienzle, 2005 #115]
HO-1 (GT<28)	Myanmarese	Cerebral vs Uncomplicated Malaria	CM = 30; UM = 120	OR = 3.14; p < 0.008	2005	[Takeda, 2005 #590]
ICAM-1 (Kilifi)	Kenyan	Cerebral Malaria	CM = 157; Co = 287	RR = 2.23; p = 0.0042	1997	[Fernandez-Reyes, 1997 #239]
ICAM-1 (Kilifi)	Gabonese	Severe vs Mild Malaria	SM = 100; MM = 100	OR = 0.52; p = 0.012	1999	[Kun, 1999 #253]
ICAM-1 (Exon 6 G Allele)	Nigeria (Ibadan)	Severe Malaria	Ca = 69; Co = 53	3.6X Increased Risk	2005	[Amodu, 2005 #113]
IFNG (+2200C)	Gambian (Mandinka)	Severe Malaria	Ca = 305; Co = 459	OR = 2.29; p = 0.01	2005	[Koch, 2005 #245]
IFNG (-1616C)	Gambian (Mandinka)	Cerebral Malaria	Ca = 122, Co = 459	OR = 1.36; p = 0.05	2005	[Koch, 2005 #245]
IFNAR1 (17470 & L168V)	Gambian	Cerebral Malaria	CM = 319; Co = 554	OR < 0.69; p < 0.011	2003	[Aucan, 2003 #226]
IFNAR1 (17470 & L168V)	Gambian	Severe Malaria	SM = 528; MM = 554	OR < 0.76; p < 0.031	2003	[Aucan, 2003 #226]
IFNGR1 (-56T/C)	Gambian (Mandinka)	Cerebral Malaria	CM = 123; Co = 174	OR = 0.54; p = 0.016	2002	[Koch, 2002 #244]
IFNGR1 (-470 TT/del)	Gambian (Mandinka)	Severe Malaria	SM = 238; Co = 174	OR = 0.58; p = 0.017	2002	[Koch, 2002 #244]
IL13 (-1055C/T)	Thai	Severe Malaria vs Mild Malaria	SM = 164, MM = 197	OR = 0.51; p = 0.0032	2003	[Ohashi, 2003 #264]
IL1α	Gambian	Mild Malaria	**?**	p = 0.035	2004	[Walley, 2004 #225]
IL1β	Ghanaian	Parasitemia in Uncomplicated Malaria	UM = 107; AM = 102	Increased in UM; p = 0.01	2002	[Gyan, 2002 #246]
IL1β	Gambian	Severe Malaria	**?**	p = 0.03	2004	[Walley, 2004 #225]
IL12p40 (Prom polymorphism)	Tanzanian	Death due to Cerebral Malaria	Ca = 82; Co = 96	OR = 5.04; p = 0.013	2002	[Morahan, 2002 #280]
IL22 Haplotype 3 (AGTAT)	Gambian	Severe Malaria	Ca = 676; Co = 459	OR = 0.68; p = 0.004	2005	[Koch, 2005 #245]
IL22 Haplotype 4 (GGCTT)	Gambian	Severe Malaria	Ca = 676; Co = 459	OR = 1.44; p = 0.006	2005	[Koch, 2005 #245]
IL4 (-542T)	West African (Fulani)	Anti-Malarial IgG Levels	Ca = 159	Elevated IgG; p = 0.01	2001	[Luoni, 2001 #279]
IL4 (Intron 3 VNTR)	Ghanaian	Cerebral Malaria	CM = 112; Co = 134	OR = 8.7; p < 0.0001	2004	[Gyan, 2004 #247]
MBL (Codon 54 & 57)	Gabonese	Mild vs Severe Malaria	**??SM = 100; MM = 100**	Increased in SM; p = 0.04	1998	[Luty, 1998 #276]
MBL	Ghanaian	Parasitemia in Complicated Malaria	Ca = 214	Increased parasitemia p = 0.02	2003	[Garred, 2003 #281]
MIF (CAAT prom variant)	Zambian	Malarial Parasitemia	40 Inds	p = 0.04	2005	[Zhong, 2005 #294]

Comprehensive listing of candidate gene studies for malaria. Table maintained at http://www.cimr.cam.ac.uk/blackwell

On-line Supplementary Table 3 - Malaria

NOS2A (CCTTT<11)	Gambian	Fatal Malaria vs CM, SMA, MM or Co	= 60; CM = 230; SMA = 100; MM = 276; Co = 2	OR >1.9; p < 0.04	1998	[Burgner, 1998 #218]
NOS2A (-969G/C)	Gabonese	Severe vs Mild Malaria	SM = 100; MM = 100	RR = 0.67; p = 0.04	1998	[Kun, 1998 #254]
NOS2A (CCTTT>15)	Thai	Severe Malaria vs Mild Malaria	SM = 256; MM = 179	OR = 2.14; p = 0.0029	2002	[Ohashi, 2002 #262]
NOS2A (-1173C/T)	Kenyan	Severe Malarial Anaemia	SMA = 144; no SMA = 916	RR = 0.25; p = 0.0005	2002	[Hobbs, 2002 #258]
NOS2A (-1173C/T)	Tanzania	Falciparum Malaria	FM = 134; Con = 45	OR = 0.12; p = 0.0006	2002	[Hobbs, 2002 #258]
NOS2A (1659T)	Gambian	Cerebral Malaria	334 trios	X2 = 7.78; p = 0.009	2003	[Burgner, 2003 #217]
NOS2A (1659TT)	Gambian	Cerebral Malaria	CM = 246; Co = 259	OR = 1.79; p = 0.03	2003	[Burgner, 2003 #217]
NOS2A (CCTTT>13)	Ghanaian	Severe Malaria vs Healthy Controls	Ca = 290; Co = 290	OR = 1.7; p = 0.03	2004	[Cramer, 2004 #121]
NOS2A (CCTTT16)	Ghanaian	Severe Malaria vs Parasitaemia	Ca = 290; Co = 290	OR = 4.5; p = 0.07	2004	[Cramer, 2004 #121]
NOS2A (-954G/C)	Ugandan	Incidence of Malaria	Ca = 184; Co = 123	IRR = 0.69; p = 0.05	2004	[Parikh, 2004 #552]
Rhesus Blood Group (E_, ee)	Brazilian (Amazonian)	Self Reported Number of Malarial Episodes	182 Ind	H = 4.499; p = 0.034	2003	[Beiguelman, 2003 #212]
SLC4A1 +/- (AE1)	Papua New Guinea	Malarial Parasitemia	Ca = 202; Co = 303	p 0.013	1987	[Cattani, 1987 #289]
SLC4A1 +/- (AE1)	Malayan Aborigines	Malaria Infection	79 Ind	Protection p < 0.05	1992	[Foo, 1992 #297]
SLC4A1 +/- (AE1)	Papua New Guinea	Cerebral Malaria	Ca = 68; Co = 68	OR = 0.00; p = 0.031	1999	[Allen, 1999 #301]
TLR4 (Asp299Gly, Thr399Ile)	Ghanaian	Severe Malaria	Ca = 290; Co = 290	OR = 1.53 & 2.78; p < 0.049	2006	[Mockenhaupt, 2006 #555]
Transferrin (Ser570Pro)	Indian (Bastar District)	*P. falciparum* Infection	473 Ind Screened	Not Given - Abstract Only	1993	[Thakur, 1993 #572]

Papers Reporting No Significant Linkage or Association

Candidate Gene	Population	Phenotype	Sample Size	Reported Results	Year	Reference
**MHC Class I Region:**						
**MHC Class II Region:**						
**MHC Class III Region:**						
LTA (+252 & +723)	Sri Lankan	Severe or Uncomplicated Malaria	SM = 35, UM = 116; Co = 84	ns	1999	[Wattavidanage, 1999 #266]
TNF (-238G/A)	Gabonese	Mild Malaria	Ca = 82; Co = 76	p > 0.05	2000	[Migot-Nabias, 2000 #230]
TNF -308	Thai	Severity of Malaria	CM = 108; SM = 162; MM = 201	ns	2001	[Hananantachai, 2001 #260]
TNF	Thai	Severity of Malaria	CM = 43; SM = 89; UM = 78; Co = 71	ns	2001	[Kikuchi, 2001 #221]
TNF (-308A & -238A)	Gabonese	Asymptomatic *P. falciparum*	Ca = 98; Co - 60	ns	2003	[Mombo, 2003 #229]
TNF (-238, -308, -376)	Ugandan	Incidence of Malaria	Ca = 184; Co = 123	ns	2004	[Parikh, 2004 #552]
TAP1	Gabonese	Mild vs Cerebral Malaria	MM = 100; SM = 100	ns	2005	[Niesporek, 2005 #290]
PSMB9	Gabonese	Mild vs Cerebral Malaria	MM = 100; SM = 100	ns	2005	[Niesporek, 2005 #290]
**Other Candidates:**						
ABO blood group (A, B, O, AB)	Nigerian	*P. falciparum* Parasitemia	Ca = 325; Co = 356	ns	1982	[Kassim, 1982 #240]
ABO Blood Groups	Indian (Bastar District)	Malarial Antibodies	258 Ind Screened	ns	1992	[Thakur, 1992 #571]
ABO Blood Groups	Colombian	*P. falciparum* or *P. vivax* Infection	Not given - Abstract only	ns	1994	[Montoya, 1994 #563]
ABO Blood Groups	Indian (Madhya Pradesh)	Malaria *per se*	Ca = 696; Co = 1399	ns	1995	[Singh, 1995 #567]
ABO Blood Group (O)	Zimbabwean	Severity of Malaria	SM = 280; NSM = 209	ns	1998	[Fischer, 1998 #553]
ABO Blood Group (O)	Gabonese	Severe vs Mild Malaria	SM = 100; MM = 100	OR = 1.5; p = 0.21	1999	[Lell, 1999 #250]
ABO Blood Group (A)	Gabonese	P. falciparum Parasitemia	Ca = 300	ns	2000	[Migot-Nabias, 2000 #230]
ABO Blood Group (O)	Gabonese	Asymptomatic Malaria	9 Families (50 Sibs)	ns	2002	[Domarle, 2002 #231]
CD31 (Leu125Val)	Kenyan	Severe Malaria	**396 indivs**	ns	2001	[Casals-Pascual, 2001 #234]
CD31 (Leu125Val)	Papua New Guinea	Severe Malaria	**442 indivs**	ns	2001	[Casals-Pascual, 2001 #234]
CR1	Gambian	Severe Malaria	**>1200 indivs**	ns	1998	[Bellamy, 1998 #237]
CR1/CD35	Gambian	Severe Malaria	SM = 463, Co = 390	ns	2003	[Zimmerman, 2003 #274]
CR1 (McCa/b)	Kenyan	Cerebral Malaria	CM = 23; Co = 23 & CM = 70; Co = 70	ns	2005	[Thathy, 2005 #559]
Duffy	Indian (Ao Nagas)	Malaria *per se*	Ca = 234; Co = 384	ns	1991	[Kar, 1991 #564]
Duffy (Fy*A/Fy*Anull)	Papua New Guinea	Malaria Infection	912 Ind	ns	1999	[Zimmerman, 1999 #273]
Duffy (Fy)	Brazilian (Rondonia)	*P. vivax* Infection	Ca = 68; Co = 59	ns	2001	[Cavasini, 2001 #561]
FcγRIIIa (176F/V)	Thai	Malaria Severity	CM = 106; SM = 154; MM = 202	ns	2002	[Omi, 2002 #214]
G6PD	Rajasthan	*P. falciparum* or *P. vivax* Infection	Ca = 1405; Co = 8028	X2 = 0.1299; p > 0.05	1992	[Jain, 1992 #562]
G6PD	Gabonese	Severe vs Mild Malaria	SM = 100; MM = 100	p > 0.05	1999	[Lell, 1999 #250]

On-line Supplementary Table 3 - Malaria

G6PD	Gabonese	Asymptomatic Malaria	9 Families (50 Sibs)	ns	2002	[Domarle, 2002 #231]
Globin	Gabonese	Severe vs Mild Malaria	SM = 100; MM = 100	p > 0.05	1999	[Lell, 1999 #250]
GLO1 (Glyoxalase)	Brazilian (Amazonian)	Asymptomaic *P. vivax*	182 Ind	ns	2003	[Beiguelman, 2003 #212]
GYPC (Delta exon 3)	Papua New Guinea	*P. falciparum* or *P. vivax* Infection	325 Ind	ns	2001	[Patel, 2001 #236]
GYPC (Delta exon 3)	Papua New Guinea	Asymptomatic *P. falciparum* & *P. vivax*	**?**	ns	2004	[Patel, 2004 #288]
Haemoglobin AS	Gabonese	Asymptomatic Malaria	9 Families (50 Sibs)	ns	2002	[Domarle, 2002 #231]
Haemoglobin	Brazilian (Amazonian)	Asymptomatic *P. vivax*	182 Ind	ns	2003	[Beiguelman, 2003 #212]
Haemoglobin AS	Ugandan	Incidence of Malaria	Ca = 184; Co = 123	ns	2004	[Parikh, 2004 #552]
Haptoglobin	Indian (Bastar District)	Malarial Antibodies	473 Ind Screened	ns	1993	[Thakur, 1993 #572]
Haptoglobin	Gambian	Severe Malaria	**1183 indivs**	ns	2002	[Aucan, 2002 #227]
Haptoglobin	Brazilian (Amazonian)	Asypromatic P. vivax	182 Ind	ns	2003	[Beiguelman, 2003 #212]
ICAM-1	Gambian	Severe Malaria	**>1200 indivs**	ns	1998	[Bellamy, 1998 #237]
ICAM-1 (Kilifi)	Thai	Severe Malaria	**?**	ns	2001	[Ohashi, 2001 #261]
ICAM-1	Senegalese	11 Malaria Related Traits	31 Pedigrees (878 Ind)	No linkage & no association	2005	[Ndiaye, 2005 #302]
ICAM-1 (Kilifi)	Kenyan	Episodes of Malaria	455 Children	IRR > 0.89; p > 0.05	2005	[Jenkins, 2005 #556]
IFNAR2	Gambian	Severe Malaria	SM = 190; Co = 190	ns	2003	[Aucan, 2003 #226]
IFNGR1 (-56T/C)	Gabonese	Mild vs Severe Malaria	SM = 95; MM = 89	ns	2003	[Juliger, 2003 #277]
IFNGR2	Gambian	Severe Malaria	SM = 190; Co = 190	ns	2003	[Aucan, 2003 #226]
IL1B (-31C/T)	Thai	Cerebral Malaria vs Mild Malaria	CM = 109; MM = 203	ns	2005	[Ohashi, 2005 #265]
IL1RA (VNTR)	Thai	Cerebral Malaria vs Mild Malaria	CM = 109; MM = 203	ns	2005	[Ohashi, 2005 #265]
IL-10 (-1082G/A)	Thai	Severity of Malaria	MM = 203, SM = 164, CM = 109	ns	2002	[Ohashi, 2002 #263]
IL10RB	Gambian	Severe Malaria	SM = 190; Co = 190	ns	2003	[Aucan, 2003 #226]
IL1RA	Gambian	Severe Malaria	**>1200 indivs**	ns	1998	[Bellamy, 1998 #26]
IL1RA (Intron 2 VNTR)	Ghanaian	Malaria Severity	Ca = 461; Co = 111	ns	2002	[Gyan, 2002 #246]
IL12p40	Kenyan	Death due to Severe Malaria	SM = 693	ns	2002	[Morahan, 2002 #280]
IL3	Thai	Severe Malaria vs Mild Malaria	SM = 64, MM = 197	ns	2003	[Ohashi, 2003 #264]
IL4	Thai	Severe Malaria vs Mild Malaria	SM = 164, MM = 197	ns	2003	[Ohashi, 2003 #264]
IL4 (589T)	Burkina Faso	Severe Malaria vs Uncomplicated Malaria	SM = 300; UM = 280	ns	2004	[Verra, 2004 #271]
Kell (K) Antigen	Brazilian (Amazonian)	Asymptomatic *P. vivax*	182 Ind	ns	2003	[Beiguelman, 2003 #212]
MBL (Codon 52, 54 & 57)	Gambian	Mild, Severe or Cerebral Malaria	CM = 368; SM = 185; MM = 292; Co = 426	ns	1998	[Bellamy, 1998 #29]
MBL (Codon 54 & 57)	Gabonese	Mild Malaria	Ca = 82; Co = 76	ns	2000	[Migot-Nabias, 2000 #230]
MBL	Ghanaian	Severe, Cerebral or Uncomplicated Malaria	SM = 73; CM = 141; UM = 109	p = 0.70	2003	[Garred, 2003 #281]
MBL (Codon 54 & 57)	Gabonese	Asymptomatic *P. falciparum*	Ca = 98; Co - 60	ns	2003	[Mombo, 2003 #229]
MBL	Ghanaian	Malaria Infection	Ca = 147; Co = 81	p = 0.84	2003	[Garred, 2003 #281]
MNSs	Brazilian (Amazonian)	Asyptomatic *P. vivax*	182 Ind	ns	2003	[Beiguelman, 2003 #212]
NOS2A (-954G/C, CCTTT(n))	Tanzanian	Severity of Malaria	CM = 82; MM = 52; Co = 44	ns	1999	[Levesque, 1999 #257]
NOS2A (-954C)	Gabonese	Asymptomatic *P. falciparum*	Ca = 98; Co - 60	ns	2003	[Mombo, 2003 #229]
NOS2A (-954, -1173, CCTTT(n))	Ghanaian	Nitric oxide levels in Cerebral Malaria	Ca = 85; Co = 125	ns	2005	[Cramer, 2005 #122]
PGM1 (Phosphoglucomutase)	Brazilian (Amazonian)	Asyptomatic *P. vivax*	182 Ind	ns	2003	[Beiguelman, 2003 #212]
SLC4A1 +/- (AE1)	Papua New Guinea	Asymptomatic *P. falciparum* & *P. vivax*	**?**	ns	2004	[Patel, 2004 #288]
TLR2 (Arg677Trp, Arg753Gln)	Ghanaian	Severe Malria	Ca = 290; Co = 290	ns	2006	[Mockenhaupt, 2006 #555]
TLR9 (T1237C, T1486C)	Ghanaian	Severe Malria	Ca = 290; Co = 290	ns	2006	[Mockenhaupt, 2006 #555]
TNFR2	Thai	Severity of Malaria	CM = 108; SM =162; MM = 201	ns	2001	[Hananantachai, 2001 #260]
**PUBMED Search Term = Malaria AND susceptibility NOT drug; Field: Text Word, Limits: Humans**						
Ca = Cases						
Co = Controls						
Ind = Individuals						
ns = Not Significant						
OR = Odds Ratio						
RR = Relative Risk						

On-line Supplementary Table 3 - Malaria

$\chi2$ = Chi-Squared						
ZMLB = Z Score of the Maximum-Likelihood-Binomial						
LOD = Logarithm of the Odds						
MLB-LOD = Maximum-Likelihood-Binomial Logarithm of the Odds						
pc = Corrected p-Value						
nc = Not Corrected						
N/A = Not Available (Possibly Abstract Only Available)						
AM = Asymptomaic Malaria						
IRR = Incidence Rate Ratio						
H = Kruskal-Wallis Test Statistic						
Z =						
NSM = Non-Severe Malaria						
CM = Cerebral Malaria						
MM = Mild Malaria						
SM = Severe Malaria						
UM = Uncomplicated Malaria						
SMA = Severe Malarial Anaemia						

Bibliography for Webtable 3.

Agarwal A, Guindo A, Cissoko Y, Taylor JG, Coulibaly D, Kone A, Kayentao K, Djimde A, Plowe CV, Doumbo O, Wellems TE, Diallo D (2000) Hemoglobin C associated with protection from severe malaria in the Dogon of Mali, a West African population with a low prevalence of hemoglobin S. Blood 96:2358-63

Aidoo M, McElroy PD, Kolczak MS, Terlouw DJ, ter Kuile FO, Nahlen B, Lal AA, Udhayakumar V (2001) Tumor necrosis factor-alpha promoter variant 2 (TNF2) is associated with pre-term delivery, infant mortality, and malaria morbidity in western Kenya: Asembo Bay Cohort Project IX. Genet Epidemiol 21:201-11

Aitman TJ, Cooper LD, Norsworthy PJ, Wahid FN, Gray JK, Curtis BR, McKeigue PM, Kwiatkowski D, Greenwood BM, Snow RW, Hill AV, Scott J (2000) Malaria susceptibility and CD36 mutation. Nature 405:1015-6

Allen SJ, O'Donnell A, Alexander ND, Mgone CS, Peto TE, Clegg JB, Alpers MP, Weatherall DJ (1999) Prevention of cerebral malaria in children in Papua New Guinea by southeast Asian ovalocytosis band 3. Am J Trop Med Hyg 60:1056-60

Amodu OK, Gbadegesin RA, Ralph SA, Adeyemo AA, Brenchley PE, Ayoola OO, Orimadegun AE, Akinsola AK, Olumese PE, Omotade OO (2005) Plasmodium falciparum malaria in south-west Nigerian children: Is the polymorphism of ICAM-1 and E-selectin genes contributing to the clinical severity of malaria? Acta Trop

Aucan C, Walley AJ, Greenwood BM, Hill AV (2002) Haptoglobin genotypes are not associated with resistance to severe malaria in The Gambia. Trans R Soc Trop Med Hyg 96:327-8

Aucan C, Walley AJ, Hennig BJ, Fitness J, Frodsham A, Zhang L, Kwiatkowski D, Hill AV (2003) Interferon-alpha receptor-1 (IFNAR1) variants are associated with protection against cerebral malaria in the Gambia. Genes Immun 4:275-82

Aucan C, Walley AJ, Hill AV (2004) Common apolipoprotein E polymorphisms and risk of clinical malaria in the Gambia. J Med Genet 41:21-4

Beiguelman B, Alves FP, Moura MM, Engracia V, Nunes AC, Heckmann MI, Ferreira RG, da Silva LH, Camargo EP, Krieger H (2003) The association of genetic markers and malaria infection in the Brazilian Western Amazonian region. Mem Inst Oswaldo Cruz 98:455-60

Bellamy R, Kwiatkowski D, Hill AV (1998a) Absence of an association between intercellular adhesion molecule 1, complement receptor 1 and interleukin 1 receptor antagonist gene polymorphisms and severe malaria in a West African population. Trans R Soc Trop Med Hyg 92:312-6

Bellamy R, Ruwende C, Corrah T, McAdam KP, Whittle HC, Hill AV (1998b) Assessment of the interleukin 1 gene cluster and other candidate gene polymorphisms in host susceptibility to tuberculosis. Tuber Lung Dis 79:83-9

Bellamy R, Ruwende C, McAdam KP, Thursz M, Sumiya M, Summerfield J, Gilbert SC, Corrah T, Kwiatkowski D, Whittle HC, Hill AV (1998c) Mannose binding protein deficiency is not associated with malaria, hepatitis B carriage nor tuberculosis in Africans. Qjm 91:13-8.

Bienzle U, Eggelte TA, Adjei LA, Dietz E, Ehrhardt S, Cramer JP, Otchwemah RN, Mockenhaupt FP (2005) Limited influence of haptoglobin genotypes on severe malaria in Ghanaian children. Trop Med Int Health 10:668-71

Brouwer KC, Lal AA, Mirel LB, Otieno J, Ayisi J, Van Eijk AM, Lal RB, Steketee R, Nahlen BL, Shi YP (2004) Polymorphism of Fc receptor IIa for immunoglobulin G is associated with placental malaria in HIV-1-positive women in western Kenya. J Infect Dis 190:1192-8

Burgner D, Usen S, Rockett K, Jallow M, Ackerman H, Cervino A, Pinder M, Kwiatkowski DP (2003) Nucleotide and haplotypic diversity of the NOS2A promoter region and its relationship to cerebral malaria. Hum Genet 112:379-86

Burgner D, Xu W, Rockett K, Gravenor M, Charles IG, Hill AV, Kwiatkowski D (1998) Inducible nitric oxide synthase polymorphism and fatal cerebral malaria. Lancet 352:1193-4

Casals-Pascual C, Allen S, Allen A, Kai O, Lowe B, Pain A, Roberts DJ (2001) Short report: codon 125 polymorphism of CD31 and susceptibility to malaria. Am J Trop Med Hyg 65:736-7

Cattani JA, Gibson FD, Alpers MP, Crane GG (1987) Hereditary ovalocytosis and reduced susceptibility to malaria in Papua New Guinea. Trans R Soc Trop Med Hyg 81:705-9

Cavasini CE, Tarelho Pereira FJ, Ribeiro WL, Wunderlich G, Ferreira MU (2001) Duffy blood group genotypes among malaria patients in Rondonia, Western Brazilian Amazon. Rev Soc Bras Med Trop 34:591-5

Cockburn IA, Mackinnon MJ, O'Donnell A, Allen SJ, Moulds JM, Baisor M, Bockarie M, Reeder JC, Rowe JA (2004) A human complement receptor 1 polymorphism that reduces Plasmodium falciparum rosetting confers protection against severe malaria. Proc Natl Acad Sci U S A 101:272-7

Cooke GS, Aucan C, Walley AJ, Segal S, Greenwood BM, Kwiatkowski DP, Hill AV (2003) Association of Fcgamma receptor IIa (CD32) polymorphism with severe malaria in West Africa. Am J Trop Med Hyg 69:565-8

Cramer JP, Mockenhaupt FP, Ehrhardt S, Burkhardt J, Otchwemah RN, Dietz E, Gellert S, Bienzle U (2004) iNOS promoter variants and severe malaria in Ghanaian children. Trop Med Int Health 9:1074-80

Cramer JP, Nussler AK, Ehrhardt S, Burkhardt J, Otchwemah RN, Zanger P, Dietz E, Gellert S, Bienzle U, Mockenhaupt FP (2005) Age-dependent effect of plasma nitric oxide on parasite density in Ghanaian children with severe malaria. Trop Med Int Health 10:672-80

Domarle O, Migot-Nabias F, Pilkington H, Elissa N, Toure FS, Mayombo J, Cot M, Deloron P (2002) Family analysis of malaria infection in Dienga, Gabon. Am J Trop Med Hyg 66:124-9

Elagib AA, Kider AO, Akerstrom B, Elbashir MI (1998) Association of the haptoglobin phenotype (1-1) with falciparum malaria in Sudan. Trans R Soc Trop Med Hyg 92:309-11

Fernandez-Reyes D, Craig AG, Kyes SA, Peshu N, Snow RW, Berendt AR, Marsh K, Newbold CI (1997) A high frequency African coding polymorphism in the N-terminal domain of ICAM-1 predisposing to cerebral malaria in Kenya. Hum Mol Genet 6:1357-60

Fischer PR, Boone P (1998) Short report: severe malaria associated with blood group. Am J Trop Med Hyg 58:122-3

Flori L, Sawadogo S, Esnault C, Delahaye NF, Fumoux F, Rihet P (2003) Linkage of mild malaria to the major histocompatibility complex in families living in Burkina Faso. Hum Mol Genet 12:375-8

Foo LC, Rekhraj V, Chiang GL, Mak JW (1992) Ovalocytosis protects against severe malaria parasitemia in the Malayan aborigines. Am J Trop Med Hyg 47:271-5

Garred P, Nielsen MA, Kurtzhals JA, Malhotra R, Madsen HO, Goka BQ, Akanmori BD, Sim RB, Hviid L (2003) Mannose-binding lectin is a disease modifier in clinical malaria and may function as opsonin for Plasmodium falciparum-infected erythrocytes. Infect Immun 71:5245-53

Gyan B, Goka B, Cvetkovic JT, Perlmann H, Lefvert AK, Akanmori B, Troye-Blomberg M (2002) Polymorphisms in interleukin-1beta and interleukin-1 receptor antagonist genes and malaria in Ghanaian children. Scand J Immunol 56:619-22

Gyan BA, Goka B, Cvetkovic JT, Kurtzhals JL, Adabayeri V, Perlmann H, Lefvert AK, Akanmori BD, Troye-Blomberg M (2004) Allelic polymorphisms in the repeat and promoter regions of the interleukin-4 gene and malaria severity in Ghanaian children. Clin Exp Immunol 138:145-50

Hananantachai H, Patarapotikul J, Looareesuwan S, Ohashi J, Naka I, Tokunaga K (2001) Lack of association of -308A/G TNFA promoter and 196R/M TNFR2 polymorphisms with disease severity in Thai adult malaria patients. Am J Med Genet 102:391-2

Hananantachai H, Patarapotikul J, Ohashi J, Naka I, Looareesuwan S, Tokunaga K (2005) Polymorphisms of the HLA-B and HLA-DRB1 Genes in Thai Malaria Patients. Jpn J Infect Dis 58:25-8

Hill AV, Allsopp CE, Kwiatkowski D, Anstey NM, Twumasi P, Rowe PA, Bennett S, Brewster D, McMichael AJ, Greenwood BM (1991) Common west African HLA antigens are associated with protection from severe malaria. Nature 352:595-600

Hobbs MR, Udhayakumar V, Levesque MC, Booth J, Roberts JM, Tkachuk AN, Pole A, Coon H, Kariuki S, Nahlen BL, Mwaikambo ED, Lal AL, Granger DL, Anstey NM, Weinberg JB (2002) A new NOS2 promoter polymorphism associated with increased nitric oxide production and protection from severe malaria in Tanzanian and Kenyan children. Lancet 360:1468-75

Jain RC (1992) G-6PD deficiency in malaria endemic areas of Udaipur District in Rajasthan. J Assoc Physicians India 40:662-3

Jenkins NE, Mwangi TW, Kortok M, Marsh K, Craig AG, Williams TN (2005) A polymorphism of intercellular adhesion molecule-1 is associated with a reduced incidence of nonmalarial febrile illness in Kenyan children. Clin Infect Dis 41:1817-9

Juliger S, Bongartz M, Luty AJ, Kremsner PG, Kun JF (2003) Functional analysis of a promoter variant of the gene encoding the interferon-gamma receptor chain I. Immunogenetics 54:675-80

Kar S, Seth S, Seth PK (1991) Duffy blood groups and malaria in the Ao Nagas in Nagaland, India. Hum Hered 41:231-5

Kassim OO, Ejezie GC (1982) ABO blood groups in malaria and schistosomiasis haematobium. Acta Trop 39:179-84

Kikuchi M, Looareesuwan S, Ubalee R, Tasanor O, Suzuki F, Wattanagoon Y, Na-Bangchang K, Kimura A, Aikawa M, Hirayama K (2001) Association of adhesion molecule PECAM-1/CD31 polymorphism with susceptibility to cerebral malaria in Thais. Parasitol Int 50:235-9

Knight JC, Udalova I, Hill AV, Greenwood BM, Peshu N, Marsh K, Kwiatkowski D (1999) A polymorphism that affects OCT-1 binding to the TNF promoter region is associated with severe malaria. Nat Genet 22:145-50

Koch O, Awomoyi A, Usen S, Jallow M, Richardson A, Hull J, Pinder M, Newport M, Kwiatkowski D (2002) IFNGR1 gene promoter polymorphisms and susceptibility to cerebral malaria. J Infect Dis 185:1684-7

Koch O, Rockett K, Jallow M, Pinder M, Sisay-Joof F, Kwiatkowski D (2005) Investigation of malaria susceptibility determinants in the IFNG/IL26/IL22 genomic region. Genes Immun 6:312-8

Kun JF, Klabunde J, Lell B, Luckner D, Alpers M, May J, Meyer C, Kremsner PG (1999) Association of the ICAM-1Kilifi mutation with protection against severe malaria in Lambarene, Gabon. Am J Trop Med Hyg 61:776-9

Kun JF, Mordmuller B, Lell B, Lehman LG, Luckner D, Kremsner PG (1998) Polymorphism in promoter region of inducible nitric oxide synthase gene and protection against malaria. Lancet 351:265-6

Lell B, May J, Schmidt-Ott RJ, Lehman LG, Luckner D, Greve B, Matousek P, Schmid D, Herbich K, Mockenhaupt FP, Meyer CG, Bienzle U, Kremsner PG (1999) The role of red blood cell polymorphisms in resistance and susceptibility to malaria. Clin Infect Dis 28:794-9

Levesque MC, Hobbs MR, Anstey NM, Vaughn TN, Chancellor JA, Pole A, Perkins DJ, Misukonis MA, Chanock SJ, Granger DL, Weinberg JB (1999) Nitric oxide synthase type 2 promoter polymorphisms, nitric oxide production, and disease severity in Tanzanian children with malaria. J Infect Dis 180:1994-2002

Luoni G, Verra F, Arca B, Sirima BS, Troye-Blomberg M, Coluzzi M, Kwiatkowski D, Modiano D (2001) Antimalarial antibody levels and IL4 polymorphism in the Fulani of West Africa. Genes Immun 2:411-4

Luty AJ, Kun JF, Kremsner PG (1998) Mannose-binding lectin plasma levels and gene polymorphisms in Plasmodium falciparum malaria. J Infect Dis 178:1221-4

May J, Lell B, Luty AJ, Meyer CG, Kremsner PG (2001) HLA-DQB1*0501-restricted Th1 type immune responses to Plasmodium falciparum liver stage antigen 1 protect against malaria anemia and reinfections. J Infect Dis 183:168-72

May J, Meyer CG, Kun JF, Lell B, Luckner D, Dippmann AK, Bienzle U, Kremsner PG (1999) HLA class II factors associated with Plasmodium falciparum merozoite surface antigen allele families. J Infect Dis 179:1042-5

McGuire W, Hill AV, Allsopp CE, Greenwood BM, Kwiatkowski D (1994) Variation in the TNF-alpha promoter region associated with susceptibility to cerebral malaria. Nature 371:508-10

McGuire W, Knight JC, Hill AV, Allsopp CE, Greenwood BM, Kwiatkowski D (1999) Severe malarial anemia and cerebral malaria are associated with different tumor necrosis factor promoter alleles. J Infect Dis 179:287-90

Meyer CG, May J, Luty AJ, Lell B, Kremsner PG (2002) TNFalpha-308A associated with shorter intervals of Plasmodium falciparum reinfections. Tissue Antigens 59:287-92

Migot-Nabias F, Mombo LE, Luty AJ, Dubois B, Nabias R, Bisseye C, Millet P, Lu CY, Deloron P (2000) Human genetic factors related to susceptibility to mild malaria in Gabon. Genes Immun 1:435-41

Miller LH, Mason SJ, Clyde DF, McGinniss MH (1976) The resistance factor to Plasmodium vivax in blacks. The Duffy-blood-group genotype, FyFy. N Engl J Med 295:302-4

Minang JT, Gyan BA, Anchang JK, Troye-Blomberg M, Perlmann H, Achidi EA (2004) Haptoglobin phenotypes and malaria infection in pregnant women at delivery in western Cameroon. Acta Trop 90:107-14

Mockenhaupt FP, Cramer JP, Hamann L, Stegemann MS, Eckert J, Oh NR, Otchwemah RN, Dietz E, Ehrhardt S, Schroder NW, Bienzle U, Schumann RR (2006) Toll-like receptor (TLR) polymorphisms in African children: Common TLR-4 variants predispose to severe malaria. Proc Natl Acad Sci U S A 103:177-82

Mockenhaupt FP, Ehrhardt S, Cramer JP, Otchwemah RN, Anemana SD, Goltz K, Mylius F, Dietz E, Eggelte TA, Bienzle U (2004a) Hemoglobin C and resistance to severe malaria in Ghanaian children. J Infect Dis 190:1006-9

Mockenhaupt FP, Ehrhardt S, Gellert S, Otchwemah RN, Dietz E, Anemana SD, Bienzle U (2004b) Alpha(+)-thalassemia protects African children from severe malaria. Blood 104:2003-6

Modiano D, Luoni G, Sirima BS, Simpore J, Verra F, Konate A, Rastrelli E, Olivieri A, Calissano C, Paganotti GM, D'Urbano L, Sanou I, Sawadogo A, Modiano G, Coluzzi M (2001) Haemoglobin C protects against clinical Plasmodium falciparum malaria. Nature 414:305-8

Mombo LE, Ntoumi F, Bisseye C, Ossari S, Lu CY, Nagel RL, Krishnamoorthy R (2003) Human genetic polymorphisms and asymptomatic Plasmodium falciparum malaria in Gabonese schoolchildren. Am J Trop Med Hyg 68:186-90

Montoya F, Restrepo M, Montoya AE, Rojas W (1994) Blood groups and malaria. Rev Inst Med Trop Sao Paulo 36:33-8

Morahan G, Boutlis CS, Huang D, Pain A, Saunders JR, Hobbs MR, Granger DL, Weinberg JB, Peshu N, Mwaikambo ED, Marsh K, Roberts DJ, Anstey NM (2002) A promoter polymorphism in the gene encoding interleukin-12 p40 (IL12B) is associated with mortality from cerebral malaria and with reduced nitric oxide production. Genes Immun 3:414-8

Ndiaye M, Thiam A, Ndiaye R, Angel G, Seignot P, Roussilhon C, Sarthou JL, Dieye A (1998) [Susceptibility to neuro-malaria and HLA-DR alleles in Senegal]. Dakar Med 43:25-8

Ndiaye R, Sakuntabhai A, Casademont I, Rogier C, Tall A, Trape JF, Spiegel A, Dieye A, Julier C (2005) Genetic study of ICAM1 in clinical malaria in Senegal. Tissue Antigens 65:474-80

Niesporek S, Meyer CG, Kremsner PG, May J (2005) Polymorphisms of transporter associated with antigen processing type 1 (TAP1), proteasome subunit beta type 9 (PSMB9) and their common promoter in African children with different manifestations of malaria. Int J Immunogenet 32:7-11

Ntoumi F, Mercereau-Puijalon O, Ossari S, Luty A, Reltien J, Georges A, Millet P (1997) Plasmodium falciparum: sickle-cell trait is associated with higher prevalence of multiple infections in Gabonese children with asymptomatic infections. Exp Parasitol 87:39-46

Ohashi J, Naka I, Patarapotikul J, Hananantachai H, Looareesuwan S, Tokunaga K (2001) Absence of association between the allele coding methionine at position 29 in the N-terminal domain of ICAM-1 (ICAM-1(Kilifi)) and severe malaria in the northwest of Thailand. Jpn J Infect Dis 54:114-6

Ohashi J, Naka I, Patarapotikul J, Hananantachai H, Looareesuwan S, Tokunaga K (2002a) Lack of association between interleukin-10 gene promoter polymorphism, -1082G/A, and severe malaria in Thailand. Southeast Asian J Trop Med Public Health 33 Suppl 3:5-7

Ohashi J, Naka I, Patarapotikul J, Hananantachai H, Looareesuwan S, Tokunaga K (2002b) Significant association of longer forms of CCTTT Microsatellite repeat in the inducible nitric oxide synthase promoter with severe malaria in Thailand. J Infect Dis 186:578-81

Ohashi J, Naka I, Patarapotikul J, Hananantachai H, Looareesuwan S, Tokunaga K (2003) A single-nucleotide substitution from C to T at position -1055 in the IL-13 promoter is associated with protection from severe malaria in Thailand. Genes Immun 4:528-31

Ohashi J, Naka I, Patarapotikul J, Hananantachai H, Tangpukdee N, Looareesuwan S, Tokunaga K (2005) A functional polymorphism in the IL1B gene promoter, IL1B -31C>T, is not associated with cerebral malaria in Thailand. Malar J 4:38

Omi K, Ohashi J, Naka I, Patarapotikul J, Hananantachai H, Looareesuwan S, Tokunaga K (2002a) Polymorphisms of CD36 in Thai malaria patients. Southeast Asian J Trop Med Public Health 33 Suppl 3:1-4

Omi K, Ohashi J, Patarapotikul J, Hananantachai H, Naka I, Looareesuwan S, Tokunaga K (2002b) Absence of association between the Fc gamma receptor IIIA-176F/V polymorphism and the severity of malaria in Thai. Jpn J Infect Dis 55:167-9

Omi K, Ohashi J, Patarapotikul J, Hananantachai H, Naka I, Looareesuwan S, Tokunaga K (2002c) Fcgamma receptor IIA and IIIB polymorphisms are associated with susceptibility to cerebral malaria. Parasitol Int 51:361-6

Omi K, Ohashi J, Patarapotikul J, Hananantachai H, Naka I, Looareesuwan S, Tokunaga K (2003) CD36 polymorphism is associated with protection from cerebral malaria. Am J Hum Genet 72:364-74

Pain A, Urban BC, Kai O, Casals-Pascual C, Shafi J, Marsh K, Roberts DJ (2001) A non-sense mutation in Cd36 gene is associated with protection from severe malaria. Lancet 357:1502-3

Pant CS, Gupta DK, Sharma RC, Gautam AS, Bhatt RM (1992) Frequency of ABO blood groups, sickle-cell haemoglobin, G-6-PD deficiency and their relation with malaria in scheduled castes and scheduled tribes of Kheda District, Gujarat. Indian J Malariol 29:235-9

Parikh S, Dorsey G, Rosenthal PJ (2004) Host polymorphisms and the incidence of malaria in Ugandan children. Am J Trop Med Hyg 71:750-3

Patel SS, King CL, Mgone CS, Kazura JW, Zimmerman PA (2004) Glycophorin C (Gerbich antigen blood group) and band 3 polymorphisms in two malaria holoendemic regions of Papua New Guinea. Am J Hematol 75:1-5

Patel SS, Mehlotra RK, Kastens W, Mgone CS, Kazura JW, Zimmerman PA (2001) The association of the glycophorin C exon 3 deletion with ovalocytosis and malaria susceptibility in the Wosera, Papua New Guinea. Blood 98:3489-91

Pathirana SL, Alles HK, Bandara S, Phone-Kyaw M, Perera MK, Wickremasinghe AR, Mendis KN, Handunnetti SM (2005) ABO-blood-group types and protection against severe, Plasmodium falciparum malaria. Ann Trop Med Parasitol 99:119-24

Quaye IK, Ekuban FA, Goka BQ, Adabayeri V, Kurtzhals JA, Gyan B, Ankrah NA, Hviid L, Akanmori BD (2000) Haptoglobin 1-1 is associated with susceptibility to severe Plasmodium falciparum malaria. Trans R Soc Trop Med Hyg 94:216-9

Rihet P, Flori L, Tall F, Traore AS, Fumoux F (2004) Hemoglobin C is associated with reduced Plasmodium falciparum parasitemia and low risk of mild malaria attack. Hum Mol Genet 13:1-6

Ruwende C, Khoo SC, Snow RW, Yates SN, Kwiatkowski D, Gupta S, Warn P, Allsopp CE, Gilbert SC, Peschu N, et al. (1995) Natural selection of hemi- and heterozygotes for G6PD deficiency in Africa by resistance to severe malaria. Nature 376:246-9

Sabeti P, Usen S, Farhadian S, Jallow M, Doherty T, Newport M, Pinder M, Ward R, Kwiatkowski D (2002) CD40L association with protection from severe malaria. Genes Immun 3:286-91

Shankarkumar U, Devaraj JP, Ghosh K, Karnad D, Anand K, Mohanty D (2002) HLA associations in P. falciparum malaria patients from Mumbai, western India. Indian J Malariol 39:76-82

Shi YP, Nahlen BL, Kariuki S, Urdahl KB, McElroy PD, Roberts JM, Lal AA (2001) Fcgamma receptor IIa (CD32) polymorphism is associated with protection of infants against high-density Plasmodium falciparum infection. VII. Asembo Bay Cohort Project. J Infect Dis 184:107-11

Singh N, Shukla MM, Uniyal VP, Sharma VP (1995) ABO blood groups among malaria cases from district Mandla, Madhya Pradesh. Indian J Malariol 32:59-63

Spencer HC, Miller LH, Collins WE, Knud-Hansen C, McGinnis MH, Shiroishi T, Lobos RA, Feldman RA (1978) The Duffy blood group and resistance to Plasmodium vivax in Honduras. Am J Trop Med Hyg 27:664-70

Takeda M, Kikuchi M, Ubalee R, Na-Bangchang K, Ruangweerayut R, Shibahara S, Imai S, Hirayama K (2005) Microsatellite polymorphism in the heme oxygenase-1 gene promoter is associated with susceptibility to cerebral malaria in Myanmar. Jpn J Infect Dis 58:268-71

Thakur A, Verma IC (1992) Malaria and ABO blood groups. Indian J Malariol 29:241-4

Thakur A, Verma IC (1993) Serum protein polymorphisms and malaria in Madya Pradesh, India. Southeast Asian J Trop Med Public Health 24:235-8

Thathy V, Moulds JM, Guyah B, Otieno W, Stoute JA (2005) Complement receptor 1 polymorphisms associated with resistance to severe malaria in Kenya. Malar J 4:54

Ubalee R, Suzuki F, Kikuchi M, Tasanor O, Wattanagoon Y, Ruangweerayut R, Na-Bangchang K, Karbwang J, Kimura A, Itoh K, Kanda T, Hirayama K (2001) Strong association of a tumor necrosis factor-alpha promoter allele with cerebral malaria in Myanmar. Tissue Antigens 58:407-10

Uhlemann AC, Szlezak NA, Vonthein R, Tomiuk J, Emmer SA, Lell B, Kremsner PG, Kun JF (2004) DNA phasing by TA dinucleotide microsatellite length determines in vitro and in vivo expression of the gp91phox subunit of NADPH oxidase and mediates protection against severe malaria. J Infect Dis 189:2227-34

Verra F, Luoni G, Calissano C, Troye-Blomberg M, Perlmann P, Perlmann H, Arca B, Sirima BS, Konate A, Coluzzi M, Kwiatkowski D, Modiano D (2004) IL4-589C/T polymorphism and IgE levels in severe malaria. Acta Trop 90:205-9

Walley AJ, Aucan C, Kwiatkowski D, Hill AV (2004) Interleukin-1 gene cluster polymorphisms and susceptibility to clinical malaria in a Gambian case-control study. Eur J Hum Genet 12:132-8

Wattavidanage J, Carter R, Perera KL, Munasingha A, Bandara S, McGuinness D, Wickramasinghe AR, Alles HK, Mendis KN, Premawansa S (1999) TNFalpha*2 marks high risk of severe disease during Plasmodium falciparum malaria and other infections in Sri Lankans. Clin Exp Immunol 115:350-5

Welch SG, McGregor IA, Williams K (1977) The Duffy blood group and malaria prevalence in Gambian West Africans. Trans R Soc Trop Med Hyg 71:295-6
Williams TN, Wambua S, Uyoga S, Macharia A, Mwacharo JK, Newton CR, Maitland K (2005) Both heterozygous and homozygous alpha+ thalassemias protect against severe and fatal Plasmodium falciparum malaria on the coast of Kenya. Blood 106:368-71
Wozniak MA, Faragher EB, Todd JA, Koram KA, Riley EM, Itzhaki RF (2003) Does apolipoprotein E polymorphism influence susceptibility to malaria? J Med Genet 40:348-51
Young K, Frodsham A, Doumbo OK, Gupta S, Dolo A, Hu JT, Robson KJ, Crisanti A, Hill AV, Gilbert SC (2005) Inverse associations of human leukocyte antigen and malaria parasite types in two West African populations. Infect Immun 73:953-5
Zhong XB, Leng L, Beitin A, Chen R, McDonald C, Hsiao B, Jenison RD, Kang I, Park SH, Lee A, Gregersen P, Thuma P, Bray-Ward P, Ward DC, Bucala R (2005) Simultaneous detection of microsatellite repeats and SNPs in the macrophage migration inhibitory factor (MIF) gene by thin-film biosensor chips and application to rural field studies. Nucleic Acids Res 33:e121
Zimmerman PA, Fitness J, Moulds JM, McNamara DT, Kasehagen LJ, Rowe JA, Hill AV (2003) CR1 Knops blood group alleles are not associated with severe malaria in the Gambia. Genes Immun 4:368-73
Zimmerman PA, Woolley I, Masinde GL, Miller SM, McNamara DT, Hazlett F, Mgone CS, Alpers MP, Genton B, Boatin BA, Kazura JW (1999) Emergence of FY*A(null) in a Plasmodium vivax-endemic region of Papua New Guinea. Proc Natl Acad Sci U S A 96:13973-7
