# Supplementary material for: Genetic susceptibility to infectious diseases: big is beautiful, but will bigger be even better?
Source: Lancet Infect Dis. 2006 Oct;6(10):653–63. doi: 10.1016/S1473-3099(06)70601-6 (PMC2330096; doi:10.1016/S1473-3099(06)70601-6)
Supplement: Supplementary file 4 [file mmc4.pdf]

On-line Supplementary Table 4 - Leishmaniasis

Papers Reporting Significant Linkage or Association						
**Candidate Gene**	**Population**	**Phenotype**	**Sample Size**	**Reported Results**	**Year**	**Reference**
**MHC Class I Region:**						
Cw7	SE Asian Hmong	Cutaneous Leishmaniasis	?	p = 0.01	1987	[Barbier, 1987 #200]
A28; Bw22	Venezuelan	Localised Cutaneous Leishmaniasis	24 Families	p = 0.0018; 0.0122	1991	[Lara, 1991 #190]
Bw22	Venezuelan	Localised Cutaneous Leishmaniasis	Ca = 26; Co = 26	RR = 12.5; p = 0.048	1991	[Lara, 1991 #190]
A11; B5; B7	Egyptian	Diffuse Cutaneous Leishmaniasis	Ca = 27; Co = ?	N/A	1993	[el-Mogy, 1993 #196]
A25	Iranian	Visceral Leishmaniasis	Ca = 52; Co = 222	RR = 13.27; p = 0.004	1995	[Faghiri, 1995 #202]
**MHC Class II Region:**						
DQw8	Venezuelan	Localised Cutaneous Leishmaniasis	24 Families	p = 0.0364	1991	[Lara, 1991 #190]
DQw3	Venezuelan	Localised Cutaneous Leishmaniasis	Ca = 26; Co = 26	RR = 4.25; p = 0.036	1991	[Lara, 1991 #190]
DR2	Brazilian	Mucocutaneous Leishmaniasis	Ca = 43; Co = 111	RR = 0.07; p = 0.004	1991	[Petzl-Erler, 1991 #201]
DQw3	Brazilian	Mucocutaneous Leishmaniasis	Ca = 43; Co = 111	RR = 4.2; p = 0.006	1991	[Petzl-Erler, 1991 #201]
DR2; DR7/DRw9	Venezuelan	Cutaneous Leishmaniasis	Ca = 49; Co = 43	p < 0.05	1995	[Cabrera, 1995 #203]
DRB1*15; *16	Tunisian	Visceral Leishmaniasis	Ca = 156; Co = 154	RR = 0.54; p = 0.04 (p_c > 0.05)	2001	[Meddeb-Garnaoui, 2001 #207]
DQB1*0201	Tunisian	Visceral Leishmaniasis	Ca = 156; Co = 154	RR = 0.46; p = 0.03 (pc > 0.05)	2001	[Meddeb-Garnaoui, 2001 #207]
HLA-DR msat	Brazilian	Visceral Leishmaniasis	751 Individuals	p = 0.02	2002	[Karplus, 2002 #204]
DRB1*0407; DPA1*0401; DPB1*0101	Mexican Mestizos	Localised Cutaneous Leishmaniasis	Ca = 65; Co = 100	OR = 2.92, 10.07, 5.99	2004	[Olivo-Diaz, 2004 #199]
DPB1*0401; DR2	Mexican Mestizos	Localised Cutaneous Leishmaniasis	Ca = 65; Co = 100	OR = 0.38; 0.14	2004	[Olivo-Diaz, 2004 #199]
**MHC Class III Region:**						
LTA	Venezuelan	Mucocutaneous Leishmaniasis	Ca = 25; Co = 43	RR = 7.5; p < 0.001	1995	[Cabrera, 1995 #203]
TNF (-308)	Venezuelan	Mucocutaneous Leishmaniasis	Ca = 25; Co = 43	RR = 3.5; p < 0.05	1995	[Cabrera, 1995 #203]
TNF (msat/-308 haplotype)	Brazilian	DTH +ve Asymptomatic *L. chagasi*	59 transmissions	p = 0.0265	2002	[Karplus, 2002 #204]
TNF -308	Brazilian	DTH +ve Asymptomatic *L. chagasi*	36 Transmissions	p = 0.0006	2002	[Karplus, 2002 #204]
**SLC11A1 (formerly NRAMP1):**						
SLC11A1 ($GT_{(n)}$)	Sudanese	Visceral Leishmaniasis	37 families (172 ind)	LOD = 1.32; p = 0.007	2003	[Bucheton, 2003 #208]
SLC11A1 (469+14G/C)	Sudanese	Visceral Leishmaniasis	59 families (312 ind)	χ^2 = 9.03; p = 0.0027	2004	[Mohamed, 2004 #205]
**Other Candidates:**						
IFNGR1 (INT 6 m/sat, alleles 10 & 11)	Sudanese	PKDL	26 families (54 aff ind)	OR = 3.0 & OR = 2.18; p < 0.03	2003	[Mohamed, 2003 #206]
IFNGR1 (INT 6 m/sat, allele 12)	Sudanese	PKDL	26 families (54 aff ind)	OR = 0.15; p = 0.014	2003	[Mohamed, 2003 #206]
IL4 (IL4RP1; IL4RP2)	Sudanese	Visceral Leishmaniasis + PKDL	53 families (123 aff ind)	OR = 2.5 + OR = 1.68; p < 0.003	2003	[Mohamed, 2003 #206]

Comprehensive listing of candidate gene studies for leishmaniasis. Table maintained at http://www.cimr.cam.ac.uk/blackwell

On-line Supplementary Table 4 - Leishmaniasis

Papers Reporting No Significant Linkage or Association						
**Candidate Gene**	**Population**	**Phenotype**	**Sample Size**	**Reported Results**	**Year**	**Reference**
**MHC Class I Region:**						
HLA -A; -B	Eastern Indian	Visceral Leishmaniasis	Ca = 51; Co = 46	p = 0.21; p = 0.22	1997	[Singh, 1997 #192]
**MHC Class II Region:**						
DR	Eastern Indian	Visceral Leishmaniasis	Ca = 51; Co = 46	p = 0.40	1997	[Singh, 1997 #192]
DQB1; DQA1; DRB1	Brazilian	Visceral Leishmaniasis	87 families (638 ind)	ns	2002	[Peacock, 2002 #191]
**MHC Class III Region:**						
TNF (-308)	Tunisian	Visceral Leishmaniasis	Ca = 156; Co = 154	ns	2001	[Meddeb-Garnaoui, 2001 #207]
LTA	Tunisian	Visceral Leishmaniasis	Ca = 156; Co = 154	ns	2001	[Meddeb-Garnaoui, 2001 #207]
HSP70-2 (PstI)	Tunisian	Visceral Leishmaniasis	Ca = 156; Co = 154	ns	2001	[Meddeb-Garnaoui, 2001 #207]
HSP70-hom (NcoI)	Tunisian	Visceral Leishmaniasis	Ca = 156; Co = 154	ns	2001	[Meddeb-Garnaoui, 2001 #207]
LTA	Brazilian	Visceral Leishmaniasis	87 families (638 ind)	ns	2002	[Peacock, 2002 #191]
TNF (-308; -238; TNFα)	Brazilian	Visceral Leishmaniasis	87 families (638 ind)	ns	2002	[Peacock, 2002 #191]
**SLC11A1 (formerly NRAMP1):**						
SLC11A1	Brazilian	Visceral Leishmaniasis	46 families (206 ind)	ns	1997	[Blackwell, 1997 #193]
SLC11A1 ($GT_{(N)}$; 1465-85G/S)	Ethiopian	Localised Cutaneous Leishmaniasis	57 Individuals	ns	1998	[Maasho, 1998 #195]
**Other Candidates:**						
ABO & Rhesus	French Guiana	Cutaneous Leishmaniasis	Ca = 96; Co = 1945	ns	1989	[Esterre, 1989 #198]
IFNG (JAP Intronic m/sat)	Sudanese	Visceral Leishmaniasis	37 families (172 ind)	ns	2003	[Bucheton, 2003 #208]
IFNGR1	Sudanese	Visceral Leishmaniasis	53 families (123 aff ind)	ns	2003	[Mohamed, 2003 #206]
IFNGR1 (FA1 Intronic m/sat)	Sudanese	Visceral Leishmaniasis	37 families (172 ind)	ns	2003	[Bucheton, 2003 #208]
IL9	Sudanese	Visceral Leishmaniasis + PKDL	59 families (312 ind)	ns	2003	[Mohamed, 2003 #206]
**PUBMED Search Term = leishmaniasis AND susceptibility NOT drug; Field: Text Word, Limits: Humans**						
Ca = Cases						
Co = Controls						
Ind = Individuals						
ns = Not Significant						
OR = Odds Ratio						
RR = Relative Risk						
χ^2 = Chi-Squared						
ZMLB = Z Score of the Maximum-Likelihood-Binomial						
LOD = Logarithm of the Odds						
pc = Corrected p-Value						
N/A = Not Available (Possibly Abstract Only Available)						
PKDL = Post Kala Azar Dermal Leishmaniasis						
DTH = Delayed Type Hypersensitivity						

Bibliography for Webtable 4.

Barbier D, Demenais F, Lefait JF, David B, Blanc M, Hors J, Feingold N (1987) Susceptibility to human cutaneous leishmaniasis and HLA, Gm, Km markers. Tissue Antigens 30:63-7

Blackwell JM, Black GF, Peacock CS, Miller EN, Sibthorpe D, Gnanandha D, Shaw JJ, Silveira F, Lins-Lainson Z, Ramos F, Collins A, Shaw MA (1997) Immunogenetics of leishmanial and mycobacterial infections: the Belem Family Study. Philos Trans R Soc Lond B Biol Sci 352:1331-45

Bucheton B, Abel L, Kheir MM, Mirgani A, El-Safi SH, Chevillard C, Dessein A (2003) Genetic control of visceral leishmaniasis in a Sudanese population: candidate gene testing indicates a linkage to the NRAMP1 region. Genes Immun 4:104-9

Cabrera M, Shaw MA, Sharples C, Williams H, Castes M, Convit J, Blackwell JM (1995) Polymorphism in tumor necrosis factor genes associated with mucocutaneous leishmaniasis. J Exp Med 182:1259-64

el-Mogy MH, Abdel-Hamid IA, Abdel-Razic MM, Rizk RA, Romia SA (1993) Histocompatibility antigens in Egyptians with cutaneous leishmaniasis: a preliminary study. J Dermatol Sci 5:89-91

Esterre P, Dedet JP (1989) The relationship of blood-group type to American cutaneous leishmaniasis. Ann Trop Med Parasitol 83:345-8

Faghiri Z, Tabei SZ, Taheri F (1995) Study of the association of HLA class I antigens with kala-azar. Hum Hered 45:258-61

Karplus TM, Jeronimo SM, Chang H, Helms BK, Burns TL, Murray JC, Mitchell AA, Pugh EW, Braz RF, Bezerra FL, Wilson ME (2002) Association between the tumor necrosis factor locus and the clinical outcome of Leishmania chagasi infection. Infect Immun 70:6919-25

Lara ML, Layrisse Z, Scorza JV, Garcia E, Stoikow Z, Granados J, Bias W (1991) Immunogenetics of human American cutaneous leishmaniasis. Study of HLA haplotypes in 24 families from Venezuela. Hum Immunol 30:129-35

Maasho K, Sanchez F, Schurr E, Hailu A, Akuffo H (1998) Indications of the protective role of natural killer cells in human cutaneous leishmaniasis in an area of endemicity. Infect Immun 66:2698-704

Meddeb-Garnaoui A, Gritli S, Garbouj S, Ben Fadhel M, El Kares R, Mansour L, Kaabi B, Chouchane L, Ben Salah A, Dellagi K (2001) Association analysis of HLA-class II and class III gene polymorphisms in the susceptibility to mediterranean visceral leishmaniasis. Hum Immunol 62:509-17

Mohamed HS, Ibrahim ME, Miller EN, Peacock CS, Khalil EA, Cordell HJ, Howson JM, El Hassan AM, Bereir RE, Blackwell JM (2003) Genetic susceptibility to visceral leishmaniasis in The Sudan: linkage and association with IL4 and IFNGR1. Genes Immun 4:351-5

Mohamed HS, Ibrahim ME, Miller EN, White JK, Cordell HJ, Howson JM, Peacock CS, Khalil EA, El Hassan AM, Blackwell JM (2004) SLC11A1 (formerly NRAMP1) and susceptibility to visceral leishmaniasis in The Sudan. Eur J Hum Genet 12:66-74

Olivo-Diaz A, Debaz H, Alaez C, Islas VJ, Perez-Perez H, Hobart O, Gorodezky C (2004) Role of HLA class II alleles in susceptibility to and protection from localized cutaneous leishmaniasis. Hum Immunol 65:255-61

Peacock CS, Sanjeevi CB, Shaw MA, Collins A, Campbell RD, March R, Silveira F, Costa J, Coste CH, Nascimento MD, Siddiqui R, Shaw JJ, Blackwell JM (2002) Genetic analysis of multicase families of visceral leishmaniasis in northeastern Brazil: no major role for class II or class III regions of HLA. Genes Immun 3:350-8

Petzl-Erler ML, Belich MP, Queiroz-Telles F (1991) Association of mucosal leishmaniasis with HLA. Hum Immunol 32:254-60

Singh N, Sundar S, Williams F, Curran MD, Rastogi A, Agrawal S, Middleton D (1997) Molecular typing of HLA class I and class II antigens in Indian kala-azar patients. Trop Med Int Health 2:468-71
